# An annotated checklist of the vascular plants of Aberdare Ranges Forest, a part of Eastern Afromontane Biodiversity Hotspot

**DOI:** 10.3897/phytokeys.149.48042

**Published:** 2020-06-03

**Authors:** Solomon Kipkoech, David Kimutai Melly, Benjamin Watuma Muema, Neng Wei, Peris Kamau, Paul Muigai Kirika, Qingfeng Wang, Guangwan Hu

**Affiliations:** 1 CAS Key Laboratory of Plant Germplasm Enhancement and Specialty Agriculture, Wuhan Botanical Garden, Chinese Academy of Sciences, Wuhan 430074, Hubei, China National Museums of Kenya Nairobi Kenya; 2 University of Chinese Academy of Sciences. Beijing 100049, China Wuhan Botanical Garden, Chinese Academy of Sciences Wuhan China; 3 Sino-Africa Joint Research Center, Chinese Academy of Sciences, Wuhan 430074, Hubei, China University of Chinese Academy of Sciences Beijing China; 4 East African Herbarium, National Museums of Kenya, P.O. Box 45166 00100, Nairobi, Kenya Sino-Africa Joint Research Center, Chinese Academy of Sciences Wuhan China

**Keywords:** Checklist, conservation, floristic surveys, plant diversity, world flora

## Abstract

The Aberdare Ranges Forest, located in the Central highlands of Kenya, is an isolated volcanic mountain in the East African Rift Valley with unique flora. Despite its refugial importance to rare and endemic plant species, the diversity of plants in the Aberdare Ranges Forest remains poorly understood. The checklist presented here is a collation of data obtained from multiple floristic surveys and from herbarium specimen collections from the forest. A total of 1260 vascular plants taxa representing 136 families, 613 genera, 67 subspecies and 63 varieties are documented. The ferns comprised 84 species, lycophytes seven, gymnosperms six and angiosperms were 1163 taxa. This represents 17.9% of the Kenyan taxa, 1.7% of the African taxa and 0.3% of all the vascular plants known in the world. A total of 18 taxa were endemic and 14 taxa were found to be threatened globally. The life form, voucher specimen(s), habitat and distribution range of each taxon and a brief analysis of taxa diversity is presented in this checklist. This is the first comprehensive inventory of vascular plants in the entire Aberdare Ranges, providing a solid basis for more sustainable management and improved conservation of this montane forest. The checklist is also an important contribution to the world checklist of plants required by the Global Strategy for Plant Conservation.

## Introduction

Tropical montane forests have been classified as biodiversity hotspots (Clark et al. 1999; Myers et al. 2000; Murphy and Bowman 2012) and they represent the Earth’s most biologically rich, yet threatened areas that need to be prioritised for conservation and other biodiversity investments (Brooks et al. 2002; CEPF 2012). The Eastern Afromontane Biodiversity Hotspot (EABH) is amongst the eight known hotspots in Africa with globally significant diversity and endemism (Myers et al. 2000; CEPF 2012). This region stretches over a curving arc of widely scattered, but biogeographically similar mountains, including the East African mountains, that have attracted the interest of plants scientists due to the complex processes shaping the species richness and the accumulation of local plant diversity (Burgess et al. 2007; Chen et al. 2015; Liu et al. 2016). The East African mountains are of relatively young geological age and share similar tropical climates, but they tend to be isolated from each other (Liu et al. 2016).

Floristic explorations, with subsequent inventorying and digitisation of plants species in many parts of the world, are far from complete (Schmitt et al. 2010). The species-rich biodiversity regions in tropical Africa remain poorly sampled to date, underlining the need for thorough explorations and documentation especially with the current unprecedented rate of species extinction (Christenhusz and Byng 2016; Stropp et al. 2016; Sosef et al. 2017). Indeed, the partial and uneven species-occurrence data have created a taxonomic impediment that have substantially impacted the effective conservation and sustainable utilisation of biodiversity (Callmander et al. 2005; Küper et al. 2006). In order to halt the continuing loss of plant diversity, the Convention on Biological Diversity (CBD), through the Global Strategy for Plant Conservation (GSPC), has over the past two decades advocated for intense exploration and documentation of plants species with the aim of achieving a complete world checklist of flora in the near future (COP 2002; Paton et al. 2008; Joppa et al. 2013; Sharrock et al. 2014).

In Kenya, floristic inventorying of the highly fragmented forests (Peltorinne 2004) has been intensified in the recent past. In particular, major forest ecosystems with significant socio-economic importance have been documentend, i.e. Mount Elgon (Tweedie 1976), Taita Hills (Beentje 1988; Thijs et al. 2014), Bura Tana River (Gachathi et al. 1994), Mount Nyiru (Bytebier and Bussmann 2000), Shimba Hills (Luke 2005), Kakamega Forest (Fischer et al. 2010), Nandi Forest (Girma et al. 2015), Mount Kenya (Zhou 2017) and Cherangani Hills (Mbuni et al. 2019). In the Aberdare Ranges (AR) Forest, previous floristic studies have either focused on a single or a few selected taxa (Wimbush 1937, 1945; Agnew 1985; Chuah-Petiot 1997) or on partial regions of the mountain (Oliver and Hooker 1885; Fries and Fries 1948; Hedberg 1951; Dale and Greenway 1961; Schmitt 1991). Thus, an exhaustive inventory of plants’ taxa in the entire montane forest is lacking.

The AR Forest is the only East African mountain situated at the Equator and flanks the Gregory Rift Valley to the east (Muiruri 1978; Bennun and Njoroge 1999). The rugged terrain, deep ravines, undulating hills and permanent streams coupled with tropical climates and broad elevation gradient provides diverse microhabitats for unique and diverse vegetation communities (Schmitt 1991). Despite the enormous socio-economic values attached to the AR ecosystem (Lambrechts et al. 2003; Louppe et al. 2009; Ark and Group 2011), the future of biodiversity in this forest is increasingly threatened by adverse land-use changes and exploitation of natural resources within the forest (KWS 2010; Kipkoech et al. 2019). Significant patches of indigenous forest have been cleared to pave way for subsistence agriculture or degraded through unsustainable extraction of forest products (Butynski 1999; Lambrechts et al. 2003).

The goal of this study was to provide a broad checklist of vascular plants in the entire AR Forest. Knowledge of plant diversity in the AR is critically important in designing appropriate conservation and management interventions to curb further biodiversity loss in this ecosystem. Specific objectives were to (i) document the vascular plants of the entire AR Forest, (ii) document the endemic and threatened vascular plants in the AR Forest and (iii) document the life forms and habitats of all the vascular plants in the AR Forest.

## Materials and methods

### Study site

**Figure 1.**
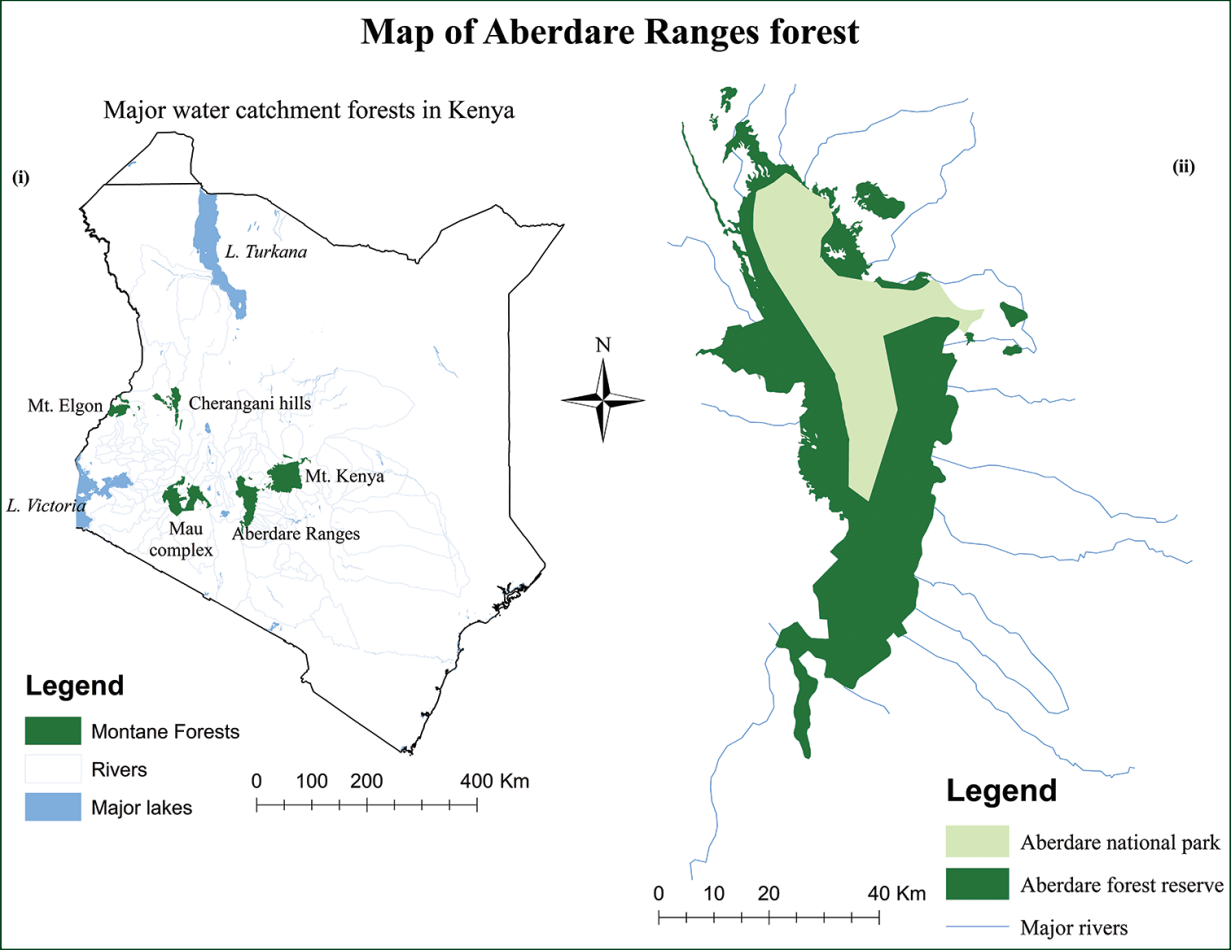
The locality of Aberdare Ranges Forest. (i) Five major water catchment forests in Kenya (ii) Two sections of the Aberdare Ranges Forest with numerous watersheds.

The Aberdare Mountain, formerly known as Nyandarua Range, is the third highest mountain in Kenya, located at the equator and forms part of the easternmost wall of the Gregory Rift Valley (Muiruri 1978; Lambrechts et al. 2003; Georgiadis et al. 2007). The AR, together with Mount Kenya, constitute the Central Highlands of Kenya. It extends approximately 120 km southwards from the equator through Nyeri, Nyandarua, Muranga and Kiambu Counties to the Kikuyu escarpment, between 36°30'E, 0°05'S and 36°55'E, 0°45'S (Schmitt 1991; KFS 2010) (Fig. 1). The altitude ranges between 1800–4001 m a.s.l. The two highest peaks, Oldonyo Lesatima (4,001 m a.s.l.) on the northern side and Il Kinangop (3,906 m a.s.l.) on the southern side of the AR, are co-joined by a flat ramp slightly tilted to the east above 3000 m elevation (Bennun and Njoroge 1999). The AR covers an area of 2,162 km^2^ with a boundary perimeter of 565 km (Butynski 1999).

The general topography of the AR is diverse and comprises numerous undulating hills formed through volcanism and faulting of the earth’s surface from the early Tertiary to the Pleistocene periods (Peltorinne 2004). On the eastern side, the slopes are gradual, while on the western side, the slopes drop dramatically to Kinangop plateau and finally to the Gregory Rift Valley. The AR Forest is governed by two institutions, Kenya Wildlife Service (KWS), which manages the Aberdare National Park with an area of ca. 76,700 ha and the Kenya Forest Service (KFS) which governs the Aberdare Forest Reserve measuring ca. 139,500 ha. The National Park encompasses the summit of the AR above 3000 m a.s.l. and a narrow salient extending to the east to 1900 m a.s.l. (Chuah-Petiot 1997; Butynski 1999). The regions below and around the Park constitute the Forest Reserve, composed of three major forest blocks; Aberdare block, Kikuyu Escarpment block and Kipipiri Forest block (Butynski 1999; KWS 2010).

The AR harbours a tropical montane forest characterised by discontinuous vegetation structures along altitudinal and climatic gradients (Hedberg 1951; Lovett 1996). Four broad vegetation zones have been described, including montane humid forest at lower altitudes, sub-montane forest at mid elevations, sub-alpine vegetation at the moorlands and dry xeromorphic evergreen forest at the northern parts of the forest (Butynski 1999). The climate in the AR Forest is influenced by the Inter-Tropical Convergence Zone north and south during its annual cycle, producing a bimodal rainfall distribution (CEPF 2012). Long rainfall periods are experienced from March to May, while short rainfall periods are received from October to November. Annual rainfall varies with altitude and exposure to the dominant winds from the Indian Ocean, ranging from 700 mm on the drier north-western slopes to 3000 mm on the wetter south-eastern slopes (Schmitt 1991; Chuah-Petiot 1997; Lambrechts et al. 2003). The average temperature in the AR decreases with increasing altitude. The mean daily temperature varies between 10.3° to 25.8 °C, with the lowest temperatures experienced between July and August (KFS 2010; CEPF 2012).

### Floristic surveys and checklist collation

A botanical team from the National Museums of Kenya (NMK) and Sino-Africa Joint Research Center (SAJOREC) carried out field investigations from 2016 to 2019. Floristic surveys were done during both wet and dry seasons to capture a wide range of phenological cycles of taxa, especially flowering and fruiting. General walk-over surveys were used in specimen collection and habitat characterisation (Gonçalves and Goyder 2016 and references therein). Field investigations targeted pristine areas not surveyed before, as well as thorough re-surveys of previously investigated areas. Plant specimens were collected in quadruplicates and each specimen was tagged with labels containing its scientific name, collection date, location and herbarium specimen number. Fertile voucher specimens with either flower, fruit or both were collected and identified with the names of taxa recorded in the field and confirmation done at the East African herbarium (EA). Standard botanical references were used in identification of specimens, i.e. Flora of Tropical East Africa (FTEA 1952–2012), Blundell (1992), Agnew and Agnew (1994), Beentje et al. (1994) and Agnew (2013). Specimens were preserved by pressing and drying. A pair of each of the taxon collected was deposited in the EA herbarium in Nairobi and another in the Wuhan Botanical Garden herbarium (HIB) in China. Herbarium acronyms follow Thiers (2020 onward: http://sweetgum.nybg.org/science/ih/).

Vascular plants specimens, previously collected for varied purposes from the AR and deposited in the EA, were compiled with our collections to develop a checklist of vascular plants. Habitat(s) and relative distribution range for each taxon were determined using our collections, herbarium specimens in the EA and published bibliographies. The broadest altitude ranges, that is between the minimum and maximum altitude a taxon is known to occur, were searched and recorded. Thence, the distribution ranges of plants’ taxa were not restricted to the AR as some taxa had wide distribution ranges extending to the sea level. Life forms of taxa collected were categorised as herbs (plants less than 50 cm high or less than 100 cm, but annual and without persistent woody stems), shrubs (plants between 50 cm to 5 m high with woody stems branching at or near the ground), climbers (plants with twining herbaceous or woody stems) and trees (plants taller than 5 m with a clear main trunk) (Schmitt 1991; Beentje 2016). Standard bibliographies, for example, (FTEA 1952–2012), Blundell (1992), Beentje et al. (1994) and Agnew (2013), were also used to define life forms, particularly from herbarium specimens. Endemic taxa were obtained by searching all the vascular plants recorded, including existing endemics cited in literature, in an on-line occurrence database in the Global Biodiversity Information Facility (GBIF) (https://www.gbif.org). Moreover, the conservation status of all the vascular plants recorded were assessed in the IUCN Red List of Threatened Species (https://www.iucnredlist.org) and categorised as Critically Endangered (CR), Endangered (EN), Vulnerable (VU) and Near Threatened (NT). The current taxonomic circumscription of each taxon recorded was checked in the Tropicos database (http://www.tropicos.org/), African Plant Database (http://www.ville-ge.ch/musinfo/bd/cjb/africa/recherche.php?langue=an) and the Catalogue of Life, 2019 Annual Checklist (http://www.catalogueoflife.org/). Finally, the recorded plants’ taxa were grouped into their respective classes and families and presented alphabetically.

## Results

### Taxa diversity

**Figure 2.**
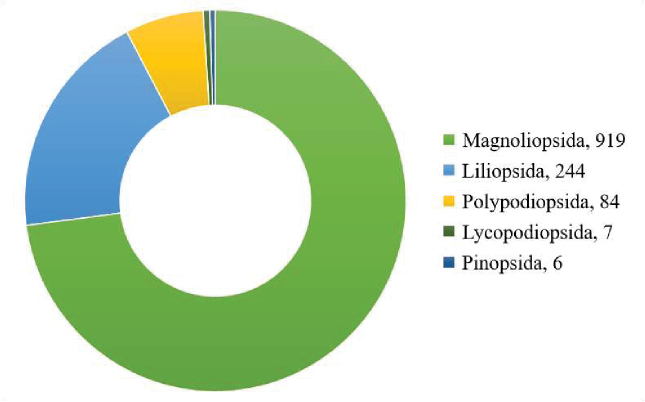
The classes of vascular plants taxa in Aberdare Ranges Forest.

A total of 1260 vascular plants representing 136 families, 613 genera, 67 subspecies and 63 varieties were recorded, which represented indigenous, naturalised, exotic or introduced plants in the AR. Ferns and fern-allies were 91 in total, with 1169 taxa of seed plants. The most diverse class was Magnoliopsida (72.9% of the total species recorded), followed by Liliopsida (19.4%), then Polypodiopsida (6.6%), Lycopodiopsida (0.6%) and the least diverse was Pinopsida (0.5%) of the total taxa recorded (Fig. 2). The top taxa-rich families were Asteraceae (11.1%), Poaceae (8.2%), Fabaceae (6.6%) and Lamiaceae (3.9%) of the total vascular plants recorded (Table 1). The most diverse genera were *Cyperus* (20), *Helichrysum* (19), *Senecio* (17), *Asplenium* (17), *Crotalaria* (15) and *Solanum* (15), while other genera had less than 14 taxa (Table 1).

**Table 1.** The 10 largest families and genera of vascular plants in Aberdare Ranges Forest.

**Family**	**Taxa**	**Genera**	**Genus**	**Taxa**	**Family**
Asteraceae	141	58	* Cyperus *	20	Cyperaceae
Poaceae	103	48	* Helichrysum *	19	Asteraceae
Fabaceae	83	32	* Senecio *	17	Asteraceae
Lamiaceae	49	18	* Asplenium *	17	Aspleniaceae
Cyperaceae	46	8	* Crotalaria *	15	Fabaceae
Rubiaceae	37	20	* Solanum *	15	Solanaceae
Orchidaceae	34	15	* Carex *	14	Cyperaceae
Euphorbiaceae	33	16	* Euphorbia *	12	Euphorbiaceae
Brassicaceae	29	18	* Plectranthus *	12	Lamiaceae
Apiaceae	27	16	* Alchemilla *	12	Rosaceae
			* Galium *	10	Rubiaceae

A total of 54 exotic plants’ taxa from varied origins were recorded (Appendix I). The majority of the exotic taxa were from Australia (14), followed by South America (12), Mexico (six), Europe (five), North America (three), while the other regions had less than three taxa. Taxa in the genus *Eucalyptus* were all from Australia. Herbs were the majority with 24 taxa, followed by trees 18 then shrubs and climbers with 11 and three taxa, respectively.

### Plants life forms

Herbs were the commonest life form with 723 taxa, followed by shrubs with 148, trees with 137 taxa, while other intermediate life forms had fewer taxa (Table 2). Epiphytes accounted for 2.9% while aquatic herbs were 0.5% of total taxa recorded.

**Table 2.** The life forms of vascular plants in Aberdare Ranges Forest.

Life form	Taxa	Percentage (%)
Herbs	723	57.38
Shrubs	148	11.74
Trees	137	10.87
Woody herbs or subshrubs	84	6.67
Herbaceous climbers	58	4.6
Epiphytes	36	2.86
Shrubs or small trees	35	2.78
Woody climbers or liana	33	2.62
Aquatic herbs	6	0.48

### Endemic and threatened taxa

Our checklist contains a total of six endemic taxa in the AR Forest (Appendix II). However, there are additional 12 taxa that are endemic in both the AR Forest and Mt. Kenya Forest. The two montane forests are in close proximity and form the Central Highlands of Kenya with enormous socio-economic value (Muiruri 1978; Schmitt 1991). The majority of endemic taxa were in the family Asteraceae (nine) and Apiaceae (three) (Appendix II). The herbs (nine taxa) were the dominant life form amongst endemic plants, followed by shrubs (six), then woody herbs (two taxa) and a single tree.

A total of 13 taxa were found to be threatened or near-threatened globally in the AR Forest (Appendix III). The taxa-rich families were Asteraceae (four) and Cyperaceae (three taxa). The majority of the threatened taxa were herbs (nine), then trees (three) and the remaining were shrubs (two taxa).

## Discussion

The AR Forest is a significant regional centre of plant diversity. With a total of 1,260 taxa recorded, it represents 17.9% of the total 7,004 vascular plants in Kenya, 10.2% of the 12,317 vascular plants in East Africa, 1.7% of the estimated 74,000 taxa in Africa and 0.3% of the estimated world flora of 422,127 taxa (Govaerts 2001; MEWNR 2015). Globally, lycophytes were 0.5% of the total 1,290 lycophytes, ferns were 0.8% of the total 10,560 ferns, gymnosperms were 0.6% of the total 1,079 gymnosperms, while angiosperms were 0.4% of the total 295,383 angiosperms in the world (Christenhusz and Byng 2016). The ferns correspond to 12.9% of the 706 ferns recorded in Africa, gymnosperms represent 13.6% of 44 African species and angiosperms corresponded to 3.6% of 32,424 and 2.3% of 50,136 angiosperms in Tropical Africa and Sub-Saharan Africa, respectively (Klopper et al. 2007; MEWNR 2015). However, there could be changes in this richness as new taxa are being described, other taxa are being synonymised, exotic or invasive taxa are being introduced, while other taxa become extinct (Pimm and Joppa 2015; Christenhusz and Byng 2016).

The taxa-rich families in the AR, Asteraceae, Fabaceae and Poaceae, were consistent with studies done in other regions in Kenya, such as Kakamega Forest (Fischer et al. 2010), North and South Nandi Forests (Girma et al. 2015), Mount Kenya Forest (Zhou 2017) and Cherangani Hills Forest (Mbuni et al. 2019). These families are the most diverse and widespread in Kenyan vegetation, especially in upland forests. They are also amongst the largest families of plants in Sub-Saharan Africa, Tropical Africa and in the world flora (Klopper et al. 2007; Christenhusz and Byng 2016). Herbs were the dominant life form in the AR which supports the widely reported observation of the herbaceous layer as the most diverse community in forest ecosystems (Albrecht et al. 1997; Bhattarai and Vetaas 2003; Whigham 2004; Zhao and Fang 2006; Lee et al. 2013; Trigas et al. 2013). The herbaceous layer, as an understorey community, plays a fundamental role in carbon dynamics and energy flow, nutrient cycling and influences seedling growth for the overstorey community (Roberts 2004; Whigham 2004; Gilliam 2007). Thus, the high richness of herbaceous taxa in the AR promotes the self-productivity and viability of this Afromontane ecosystem.

Endemic taxa in the Central Highlands of Kenya, comprising the AR and Mt. Kenya Forests, represent 3.1% of the total 577 endemics in Kenya and 0.8% of the total 2,350 endemics in the EABH (CEPF 2012; MEWNR 2015; Zhou et al. 2017). This signifies the critical role the AR plays in harbouring restricted taxa in unique habitats, particularly at higher elevations. Ancient isolation of the East African mountains after their formation (Chorowicz 2005) and the harsh climatic conditions associated with high elevations are thought to have facilitated taxa adaptation and speciation, resulting in the high endemic richness that was observed in this study (Lovett and Friis 1994; Lovett et al. 2000, 2005). The threatened taxa represent 1.1% of the total vascular plants in the AR and 3.4% of the total 356 threatened plants’ taxa known in Kenya (MEWNR 2015). This further exemplifies the regional and global importance of this forest, underlining the need of efficient conservation strategies. Plant species such as *Warbugia ugandensis* Sprague, *Lactuca inermis* Forssk, *Carissa spinarum* L., *Prunus africana* (Hook. Fil.) Kalkm and others, are traditionally utilised by the local community as remedies for various illnesses (Kokwaro 2009; Kamau et al. 2016a, b). With the rising popularity of herbal therapy in Kenya (Kokwaro 2009), such medicinal plants are over-exploited, hence the urgent need for close monitoring and protection to conserve their already shrinking populations. In addition, *Pinus radiata* D. Don, *Prunus africana* (Hook.f.) Kalkm and *Croton alienus* Pax are faced with logging and charcoal burning threats, thus, they should also be prioritised in conservation planning (Muiruri 1978; Lambrechts et al. 2003; KFS 2010). On the other hand, species such as *Ficus sur* Forssk., *Ficus thonningii* Blume and *Indigofera erecta* Thunb are considered sacred and they are strictly conserved by the local community living around the AR Forest (Muiruri 1978; Schmitt 1991; KWS 2010).

## Conclusions

The AR Forest harbours a notably diverse flora with endemic taxa. This study provides a floristic checklist with up-to-date nomenclature that will underpin future research into the diversity and conservation of the AR ecosystem. Varied environmental aspects can be researched within the AR, particularly with its mosaic of microhabitats, broad elevation gradient and associated temperature and rainfall variations within a short span of land. Our study provides the baseline data for such studies. In general, the checklist is a major step towards building a holistic biodiversity knowledge of the AR ecosystem.

## Checklist

An annotated checklist of the vascular plants of the AR Forest is presented below. Vascular plants are grouped into five classes and arranged alphabetically in their respective families. Families in Lycopodiopsida and Polypodiopsida are arranged, based on the Pteridophyte Phylogeny Group I system (PPG I 2016), Pinopsida is arranged according to Christenhusz et al. (2011), while Liliopsida and Magnoliopsida are arranged, based on the APG IV 2016 system (Chase et al. 2016). Taxa preceded by (^E^) are endemic while those with (*) are exotic and/or naturalised in the AR Forest. For each taxon recorded, full authority is given, life form, brief notes on habitat and distribution range, voucher specimen number and the herbarium where it was deposited. The broadest altitude range of each taxon is indicated in metres (m) which mostly extends beyond the elevation of the AR Forest. EA refers to the East African herbarium in Nairobi, Kenya, while HIB refers to Wuhan Botanical Garden herbarium in Wuhan, China. The collectors are abbreviated as follows: SK means Solomon Kipkoech, SAJIT refers to Sino-Africa Joint Investigation Team, FOKP means Flora of Kenya Project, KEFRI refers to Kenya Forestry Research Institute and EANHS stands for East Africa Natural History Society.

### Part 1. Lycopodiopsida

**F1. Lycopodiaceae**

***Austrolycopodium aberdaricum* (Chiov.) Holub** – Life form: Herb. Habitat: Upper parts of montane forest, 3000 m. Voucher: Balbo 475 (EA).

***Lycopodium clavatum* L.** – Life form: Herb. Habitat: Moist montane forest, 1500–3050 m. Vouchers: Mutangah 2 (EA), SK 0148 (EA, HIB).

***Phlegmariurus dacrydioides* (Baker) A.R.Field & Bostock** – Life form: Herb. Habitat: Woodland and riverine forest, 1550–2700 m. Voucher: Faden 69/1113 (EA).

***Phlegmariurus saururus* (Lam.) B.Øllg.** – Life form: Herb. Habitat: Near streams and damp sites in moorland, 2200–4400 m. Voucher: Hedberg 1627 (EA).

***Phlegmariurus verticillatus* (L.f.) A.R.Field & Testo** – Life form: Herb. Habitat: Moist woodland and wet forest, 950–2300 m. Vouchers: Someren s.n., Balbo 819 (EA).

**F2. Selaginellaceae**

**Selaginella goudotiana var. abyssinica (Spring) Bizzarri** – Life form: Herb. Habitat: Near waterfalls and riverbanks in evergreen forest, 750–2450 m. Vouchers: Ng’weno 15129, Kuchar 12753 (EA).

***Selaginella kraussiana* (Kunze) A.Braun** – Life form: Herb. Habitat: Moist forest, 1100–3350 m. Voucher: Lind and Agnew 5013 (EA).

### Part 2. Polypodiopsida

**F3. Aspleniaceae**

***Asplenium abyssinicum* Fée** – Life form: Herb. Habitat: often epiphytic in moist forest and damp sites in moorland, 1350–3150 m. Voucher: Kamau 364 (EA).

***Asplenium actiniopteroides* Peter** – Life form: Herb. Habitat: Rocky sites in upland forest, 2500–4250 m. Voucher: Someren 1054 (EA).

***Asplenium adamsii* Alston** – Life form: Herb. Habitat: Wet rocky sites in moorland and heath zone, 2400–3400 m. Voucher: Polhill 12026 (EA).

***Asplenium aethiopicum* (Burm.f.) Bech.** – Life form: Herb. Habitat: Epiphytic in moist forest and wooded grassland, 1150–3700 m. Voucher: Mutanga 13 (EA).

***Asplenium boltonii* Hook. ex Schelpe** – Life form: Herb. Habitat: Epiphytic in moist forest, 1200–2750 m. Voucher: Robertson et al. 3895 (EA).

***Asplenium bugoiense* Hieron.** – Life form: Herb. Habitat: Upland moist forest and along streams, 1650–2700 m. Voucher: Faden et al. 74/1346 (EA).

***Asplenium elliottii* C.H.Wright** – Life form: Herb. Habitat: Moist montane forest, 1050–3000 m. Voucher: Kuchar 12407 (EA).

***Asplenium erectum* Bory ex Willd.** – Life form: Herb. Habitat: Epiphytic in upland forest floors, 1300–2750 m. Voucher: Faden 69/004 (EA).

***Asplenium friesiorum* C.Chr.** – Life form: Herb. Habitat: Epiphytic in moist forest, swamps and along streams, 1100–3000 m. Voucher: Mutanga 10 (EA).

***Asplenium linckii* Kuhn** – Life form: Herb. Habitat: Shady places in moist forest, 1600–2700 m. Voucher: Faden 74/1317 (EA).

***Asplenium loxoscaphoides* Baker** – Life form: Herb. Habitat: Epiphytic in moist montane forest and *Hagenia* woodlands, 1850–3650 m. Voucher: Kuchar 5218 (EA).

***Asplenium monanthes* L.** – Life form: Herb. Habitat: Moist forest and bamboo thickets, 1950–3400 m. Voucher: Kamau 366 (EA).

***Asplenium praegracile* Rosenst.** – Life form: Herb. Habitat: Moist montane forest and bamboo zone, 2400–3100 m. Voucher: Faden et al. 71/880 (EA).

***Asplenium rutifolium* (P.J.Bergius) Kunze** – Life form: Herb. Habitat: Epiphytic in moist forest and riverine forest, 750–2300 m. Voucher: Faden 69/2075 (EA).

***Asplenium sandersonii* Hook.** – Life form: Herb. Habitat: Epiphytic in moist montane forest and forest margins, 680–3100 m. Voucher: Faden 69/012 (EA).

***Asplenium theciferum* (Kunth) Mett.** – Life form: Herb. Habitat: Epiphytic in moist forest and bush thickets, 850–2900 m. Voucher: Kibui 50 (EA).

***Asplenium uhligii* Hieron.** – Life form: Herb. Habitat: Epiphytic in upland woodland, 2400–4200 m. Voucher: Kuchar 13077 (EA).

**F4. Athyriaceae**

***Athyrium newtonii* Baker** – Life form: Herb. Habitat: Upland rocky forest margins, 1150–3500 m. Voucher: Tweedie 1882 (EA).

***Athyrium scandicinum* (Willd.) C.Presl** – Life form: Herb. Habitat: Moist forest and bamboo zone, 1150–3500 m. Voucher: Faden 70/63 (EA).

***Deparia boryana* (Willd.) M.Kato** – Life form: Herb. Habitat: Upland moist forest, 1460–2550 m. Voucher: FOKP 1983 (EA, HIB).

**F5. Blechnaceae**

***Blechnum australe* L.** – Life form: Herb. Habitat: Mixed bamboo forest and wet grassland, 1500–2500 m. Vouchers: Cameron 18, Gilbert 6315 (EA).

***Blechnum tabulare* (Thunb.) Kuhn** – Life form: Herb. Habitat: Mixed bamboo forest and wet grassland, 1600–2600 m. Voucher: Gilbert 6337 (EA).

**F6. Cyatheaceae**

***Cyathea manniana* Hook.** – Life form: Tree. Habitat: Moist forest and along streams, 1500–2500 m. Voucher: Napper 721 (EA).

**F7. Cystopteridaceae**

***Cystopteris fragilis* (L.) Bernh.** – Life form: Herb. Habitat: Damp rocky sites in evergreen forest, 1700–3600 m. Voucher: Fries & Fries 762 (EA).

**F8. Dennstaedtiaceae**

***Blotiella glabra* (Bory) R.M.Tryon** – Life form: Herb. Habitat: Moist forest, 1350–3000 m. Voucher: Faden 71/202 (EA).

***Hypolepis goetzei* Reimers** Life form: – Herb. Habitat: Moist forest, 2100–3050 m. Voucher: Faden 71/886 (EA).

***Hypolepis sparsisora* (Schrad.) Kuhn** – Life form: Herb. Habitat: Moist forest, 900–2800 m. Voucher: Verdcourt 3989 (EA).

***Pteridium aquilinum* (L.) Kuhn** – Life form: Herb. Habitat: Wooded grassland and forest margins, 2640–2850 m. Vouchers: Otieno 12609, Kuchar 7827 (EA).

**F9. Dryopteridaceae**

**Arachniodes webbiana var. foliosa (C.Chr.) Gibby, Rasbach, Reichst., Widén & Viane** – Life form: Herb. Habitat: Moist forest and streams banks, 1380–2600 m. Vouchers: Gardner 967, Kamau 471 (EA).

***Dryopteris antarctica* (Baker) C.Chr.** – Life form: Herb. Habitat: Montane grassland and moorland, 2500–3320 m. Voucher: Faden 69/803 (EA).

***Dryopteris fadenii* Pic.Serm.** – Life form: Herb. Habitat: Moist forest and riverine forest, 1700–2500 m. Voucher: Faden et al. 69/900 (EA).

***Dryopteris lewalleana* Pic.Serm.** – Life form: Herb. Habitat: Moist forest and riverine forest, 1400–2300 m. Voucher: Faden 70/55 (EA).

***Dryopteris manniana* (Hook.) C.Chr.** – Life form: Herb. Habitat: Moist forest, 1450–2250 m. Voucher: Faden et al. 69/289 (EA).

***Elaphoglossum angulatum* (Blume) T.Moore** – Life form: Herb. Habitat: Epiphytic in moist montane forest, 2470–2750 m. Voucher: Faden 71/201 (EA).

***Elaphoglossum aubertii* (Desv.) T.Moore** – Life form: Herb. Habitat: Epiphytic in moist montane forest, 1400–2800 m. Voucher: Someren 387 (EA).

***Elaphoglossum conforme* (Sw.) Schott** – Life form: Herb. Habitat: Moist montane forest, 2100–3000 m. Voucher: Balbo 769 (EA).

***Elaphoglossum deckenii* (Kuhn) C.Chr.** – Life form: Herb. Habitat: Epiphytic in moist montane forest, 2100–3500 m. Voucher: Hedberg 1635 (EA).

***Elaphoglossum hybridum* (Bory) Brack.** – Life form: Herb. Habitat: Moist forest and damp places in the moorland, 1800–3600 m. Voucher: Faden et al. 74/1340 (EA).

***Elaphoglossum lastii* (Baker) C.Chr.** – Life form: Herb. Habitat: Epiphytic in moist montane forest, 1500–2600 m. Voucher: Faden et al. 74/1324 (EA).

*Elaphoglossum piloselloides (C.Presl) T.Moore – Life form: Exotic herb. Habitat: Moist montane forest, 900–2450 m. Voucher: Peter 41097 (EA).

**Elaphoglossum spatulatum var. uluguruense (Reimers) Schelpe** – Life form: Herb. Habitat: Moist forest and along streams, 900–2450 m. Voucher: Faden et al. 71/74 (EA).

***Elaphoglossum subcinnamomeum* (Christ) Hieron.** – Life form: Herb. Habitat: Upper parts of wet montane forest, 2800–3600 m. Voucher: Kenya Exploration Society 157 (EA).

***Megalastrum lanuginosa* (Willd. ex Kaulf.) Holttum** – Life form: Herb. Habitat: Moist forest near streams, 1400–2400 m. Voucher: Faden et al. 71/282 (EA).

***Nothoperanema squamisetum* (Hook.) Ching** – Life form: Herb. Habitat: Moist forest, 1850–2950 m. Voucher: Molesworth Allen 3638 (EA).

***Polystichum sinense* (Christ) Christ** – Life form: Herb. Habitat: Upland moist forest, 1920–4100 m. Voucher: Mwangangi 987 (EA).

***Polystichum transvaalense* N.C.Anthony** – Life form: Herb. Habitat: Moist forest, 1350–2700 m. Voucher: Andrew 4461 (EA).

***Polystichum volkensii* (Hieron.) C.Chr.** – Life form: Herb. Habitat: Moist montane forest and *Hagenia* forest, 2800–3600 m. Voucher: Rabb et al. 7 (EA).

***Polystichum wilsonii* Christ** – Life form: Herb. Habitat: Shaded grounds in moist forest, 2320–3650 m. Voucher: Someren 1053 (EA).

**F10. Equisetaceae**

***Equisetum ramosissimum* Desf**. – Life form: Herb. Habitat: Along streams and rivers, 150–2100 m. Voucher: Greenway 13100 (EA).

**F11. Hymenophyllaceae**

***Crepidomanes melanotrichum* (Schltdl.) J.P.Roux** – Life form: Herb. Habitat: Shades in moist forest, 750–2650 m. Voucher: Beentje 2950 (EA).

***Crepidomanes ramitrichum* (Faden) Beentje** – Life form: Herb. Habitat: Upland moist forest and rocky waterfalls, 2300–2600 m. Voucher: Faden & Grumbley 72/338 (EA).

***Didymoglossum erosum* (Willd.) J.P.Roux** – Life form: Herb. Habitat: Shady sites in moist forest, 0–2400 m. Voucher: Faden et al. 74/1336 (EA).

**Hymenophyllum capillare var. alternialatum (Pic.Serm.) Faden** – Life form: herb. Habitat: Epiphytic in moist montane forest, 1650–3480 m. Voucher: Agnew et al. 5620 (EA).

**Hymenophyllum polyanthos var. kuhnii (C.Chr.) Schelpe** – Life form: Herb. Habitat: Epiphytic in moist montane forest, 1400–3000 m. Voucher: Faden et al. 71/199 (EA).

***Hymenophyllum tunbrigense* (L.) Sm.** – Life form: Herb. Habitat: Epiphytic in upland moist forest, 1900–2700 m. Voucher: Faden et at. 74/1338 (EA).

***Polyphlebium borbonicum* (Bosch) Ebihara & Dubuisson** – Life form: Herb. Habitat: Moist forest and stream banks, 1400–2600 m. Voucher: Faden et al. 71/196 (EA).

**F12. Marsileaceae**

***Marsilea minuta* L.** – Life form: Herb. Habitat: Aquatic in shallow water pools, edges of streams and seasonal swampy grassland, 0–1950 m. Voucher: Perkins 11502 (EA).

**F13. Ophioglossaceae**

**Ophioglossum vulgatum subsp. africanum Pocock ex J.E.Burrows** – Life form: Herb. Habitat: Montane grassland, 1000–3250 m. Vouchers: Dale 1321, Faden & Faden 71/890 (EA).

**F14. Polypodiaceae**

***Drynaria volkensii* Hieron.** – Life form: Herb. Habitat: Riverine forest and woodland, 1600–2300 m. Voucher: Kerfoot 2767 (EA).

***Grammitis cryptophlebia* (Baker) Copel.** – Life form: Herb. Habitat: Epiphytic in moist montane forest, 1900–2150 m. Voucher: Faden 71/280 (EA).

***Lepisorus excavatus* (Bory ex Willd.) Ching** – Life form: Herb. Habitat: Montane forest, 1150–3490 m. Voucher: Kamau 373 (EA).

***Lepisorus schraderi* (Mett.) Ching** – Life form: Herb. Habitat: Upland forest, 1200–2450 m. Voucher: Faden 74/904 (EA).

***Loxogramme abyssinica* (Baker) M.G.Price** – Life form: Herb. Habitat: Epiphytic in moist forest, 900–2900 m. Voucher: Kirika et al. 70 (EA).

***Melpomene flabelliformis* (Poir.) A.R.Sm. & R.C.Moran** – Life form: Herb. Habitat: Epiphytic in bushland, upland forest and bamboo zone, 1000–4200 m. Voucher: Fries 1325 (EA).

***Pleopeltis macrocarpa* (Bory ex Willd.) Kaulf.** – Life form: Herb. Habitat: Upper parts of montane forest, 1000–3600 m. Voucher: Vorontsova 55 (EA).

**F15. Pteridaceae**

***Adiantum poiretii* Wikstr.** – Life form: Herb. Habitat: Montane forest, 1000–2700 m. Voucher: Luke 1106 (EA).

***Adiantum raddianum* C.Presl** – Life form: Herb. Habitat: Wet rocky sites in montane forest, 1000–2700 m. Vouchers: Faden 68/765 & 68/849 (EA).

***Aleuritopteris farinosa* (Forsk.) Fée** – Life form: Herb. Habitat: Swampy places in montane forest, 1460–3600 m. Voucher: Kamau 531 (EA).

***Cheilanthes bergiana* Schltdl.** – Life form: Herb. Habitat: Moist forest, 1300–2300 m. Voucher: Kamau 101 (EA).

***Cheilanthes quadripinnata* (Forssk) Kuhn** – Life form: Herb. Habitat: Rocky grounds in moist forest, 1275–2750 m. Voucher: Maas Geesteranus 5043 (EA).

***Coniogramme africana* Hieron.** – Life form: Herb. Habitat: Moist forest and along streams, 1000–2250 m. Voucher: Bytebier 3229 (EA).

***Oeosporangium viride* (Forssk.) Fraser-Jenk. & Pariyar** – Life form: Herb. Habitat: Moist montane forest and shades in bushlands, 650–2250 m. Voucher: Faden 012/2003 (EA).

***Pteris catoptera* Kunze** – Life form: Herb. Habitat: Wet or dry forest, 1000–3050 m. Voucher: Zogg 2547 (EA).

***Pteris dentata* Forssk.** – Life form: Herb. Habitat: Wet or dry forest, 1000–3000 m. Voucher: Kuchar 5203 (EA).

**F16. Tectariaceae**

***Arthropteris monocarpa* (Cordem.) C.Chr.** – Life form: Herb. Habitat: Epiphytic in moist forest and riverine forest, 1250–2450 m. Voucher: Gillett and Holttum 20096 (EA).

***Tectaria gemmifera* (Fée) Alston** – Life form: Herb. Habitat: Moist forest, 600–2550 m. Voucher: Strange 117 (EA).

**F17. Thelypteridaceae**

***Amauropelta oppositiformis* (C.Chr.) Holttum** – Life form: Herb. Habitat: Wet or swampy sites in evergreen forest, 1200–3000 m. Voucher: Bytebier 222 (EA).

***Christella dentata* (Forssk.) Brownsey & Jermy** – Life form: Herb. Habitat: Along streams and damp places in forest, 45–2200 m. Voucher: Luke 148 (EA).

***Pneumatopteris unita* (Kunze) Holttum** – Life form: Herb. Habitat: Moist montane forest and bamboo thicket, 1450–2500 m. Voucher: Kamau 466 (EA).

***Pseudocyclosorus pulcher* (Bory ex Willd.) Holttum** – Life form: Herb. Habitat: Riverine forest and swampy sites in forest, 750–2250 m. Voucher: Faden 68/988 (EA).

***Phegopteris cruciata* (Willd.) Mett. ex Kuhn** – Life form: Herb. Habitat: Moist evergreen forest and stream banks, 1450–2350 m. Voucher: Faden & Evans 69/891 (EA).

**Stegnogramma pozoi var. petiolata (Ching) Sledge** – Life form: Herb. Habitat: Wet montane forest, 2050–3350 m. Vouchers: Bytebier et al. 48, Faden & Faden 69/898 (EA).

### Part 3. Pinopsida

**F18. Cupressaceae**

*Cupressus lusitanica Mill. – Life form: Exotic tree. Habitat: Cultivated, 2600–2640 m. Voucher: Dyson 526 (EA).

***Juniperus procera* Hochst. ex Endl.** – Life form: Tree. Habitat: Upland dry evergreen forest, 1050–3250 m. Voucher: SK 0106 (EA, HIB).

**F19. Pinaceae**

*Pinus patula Schiede ex Schltdl & Cham. – Life form: Exotic tree. Habitat: Cultivated, common in moist or dry forest, 1700–3000 m. Voucher: Althof s.n. (EA).

***Pinus radiata* D.Don** – Life form: Tree. Habitat: Moist or dry forest, 1700–3000 m. Voucher: Dillon 4 (EA).

**F20. Podocarpaceae**

***Afrocarpus falcatus* (Thunb.) C.N.Page** – Life form: Tree. Habitat: Dry evergreen forest, 1250–2700 m. Voucher: SK 0123 (EA, HIB).

***Podocarpus latifolius* (Thunb.) R.Br. ex Mirb.** – Life form: Tree. Habitat: Dry evergreen forest, 1500–3350 m. Voucher: SK 0102 (EA, HIB).

### Part 4. Liliopsida

**F21. Alismataceae**

***Alisma plantago-aquatica* L.** – Life form: Herb. Habitat: Marshes and stream banks, 900–2340 m. Voucher: Lubai 14 (EA).

**F22. Amaryllidaceae**

***Scadoxus multiflorus* (Martyn) Raf.** – Life form: Herb. Habitat: Moist montane forest, 0–2700 m. Voucher: Mungai 1/83 (EA).

**F23. Araceae**

***Arisaema mildbraedii* Engl.** – Life form: Herb. Habitat: Wet and shaded places in montane forest, 1400–2620 m. Voucher: SK 0219 (EA, HIB).

***Culcasia falcifolia* Engl.** – Life form: Herbaceous climber. Habitat: Moist forest, 500–2100 m. Voucher: Luke 14154 (EA).

***Lemna minor* L.** – Life form: Herb. Habitat: Surface of water pools and slow running streams, 0–1800 m. Voucher: Verdcourt 718b (EA).

***Zantedeschia pentlandii* (R.Whyte ex W.Watson) Wittm.** – Life form: Herb. Habitat: Along streams and swamps, 1400–1800 m. Voucher: SK 0255 (EA, HIB).

**F24. Arecaceae**

***Phoenix reclinata* Jacq.** – Life form: Tree. Habitat: Open rocky slopes in rainforest, 0–3000 m. Voucher: Napier 5377 (EA).

**F25. Asparagaceae**

***Anthericum angustifolium* Hochst. ex A.Rich.** – Life form: Herb. Habitat: Upland grassland, 1800–2850 m. Voucher: Leaky 8547 (EA).

***Asparagus africanus* Lam.** – Life form: Woody climber. Habitat: Forest margins and wooded grassland, 0–3500 m. Voucher: SK 0191(EA, HIB).

***Asparagus aridicola* Sebsebe** – Life form: Woody climber. Habitat: Wooded grassland and bush thickets, 10–2750 m. Voucher: Luke 10850 (EA).

***Asparagus asparagoides* (L.) Druce** – Life form: Herbaceous climber. Habitat: Moist forest and forest margins, 1100–3000 m. Voucher: Someren 1156 (EA).

***Asparagus falcatus* L.** – Life form: Herbaceous climber. Habitat: Bush thickets and forest margins, 10–2750 m. Voucher: Zogg et al. 10/255 (EA).

***Asparagus natalensis* (Baker) J.-P.Lebrun & Stork** – Life form: Woody climber. Habitat: Dry forest and forest margins, 900–2700 m. Voucher: Luke 18171 (EA).

***Asparagus racemosus* Willd.** – Life form: Woody climber. Habitat: Forest margins and wooded grassland, 1160–2900 m. Voucher: SK 0215 (EA, HIB).

***Asparagus setaceus* (Kunth) Jessop** – Life form: Woody climber. Habitat: Forest margins, 1740–2300 m. Voucher: Verdcourt 3628 (EA).

***Chlorophytum comosum* (Thunb.) Jacques** – Life form: Herb. Habitat: Undergrowth in rainforest, 20–2450 m. Voucher: SK 0168 (EA, HIB).

***Chlorophytum polystachys* Baker** – Life form: Herb. Habitat: Open woodland, 150–2900 m. Voucher: Hooper and Townsend 1651 (EA).

***Dracaena afromontana* Mildbr.** – Life form: Tree. Habitat: Upland moist forest, 1600–2700 m. Voucher: SK 0143 (EA, HIB).

***Dracaena ellenbeckiana* Engl.** – Life form: Tree. Habitat: Rocky slopes in moist forest, 1050–2100 m. Voucher: Perdue and Kibuwa 8259 (EA).

***Dracaena steudneri* Engl.** – Life form: Tree. Habitat: Moist forest margins, 850–2300 m. Voucher: Perdue and Kibuwa 8023 (EA).

***Ornithogalum gracillimum* R.E.Fr.** – Life form: Herb. Habitat: Wet grounds in grassland and swamps, 1800–2700 m. Voucher: Polhill 406 (EA).

***Sansevieria parva* N.E.Br.** – Life form: Herb. Habitat: Dry forest and rocky sites in bushland, 1600–2200 m. Voucher: Hansen 770 (EA).

***Sansevieria Perrotii* Warb.** – Life form: Herb. Habitat: Wooded grassland, 550–1950 m. Voucher: Someren 8505 (EA).

**F26. Colchicaceae**

***Androcymbium striatum* Hochst. ex A.Rich.** – Life form: Herb. Habitat: Upland grassland and bushland, 1500–3400 m. Voucher: Rayner 66 (EA).

**Wurmbea tenuis subsp. hamiltonii (Wendelbo) B.Nord.** – Life form: Herb. Habitat: Upland grassland, 2130–2750 m. Vouchers: Chandler 2415, Rayner 66 (EA).

**F27. Commelinaceae**

***Aneilema leiocaule* K.Schum.** – Life form: Herb. Habitat: Moist forest mostly in shades, 1000–2740 m. Voucher: Dyson 570 (EA).

***Commelina africana* L.** – Life form: Herb. Habitat: Grassland and woodland, 300–2980 m. Voucher: Kerfoot 619 (EA).

***Commelina imberbis* Ehrenb. ex Hassk.** – Life form: Herb. Habitat: Grassland and bushland, 1000–2910 m. Voucher: SK 0166 (EA, HIB).

***Commelina latifolia* Hochst. ex A.Rich.** – Life form: Herb. Habitat: Upland grassland, 1250–2270 m. Voucher: Kenya Forest excursion 26 (EA).

***Commelina reptans* Brenan** – Life form: Herb. Habitat: Moist grassland, 1200–2550 m. Voucher: Faden 71/889 (EA).

***Floscopa glomerata* (Willd. ex Schult. & Schult.f.) Hassk.** – Life form: Herb. Habitat: Swampy grassland and along streams, 900–2200 m. Voucher: Bally 13225 (EA).

***Murdannia clarkeana* Brenan** – Life form: Herb. Habitat: Swampy grassland, 1500–1850 m. Voucher: Hooper et al. 1695 (EA).

***Murdannia simplex* (Vahl) Brenan** – Life form: Herb. Habitat: Grassland and bushland, 30–2200 m. Voucher: Verdcourt 561 (EA).

**F28. Cyperaceae**

***Bulbostylis glaberrima* Kük.** – Life form: Herb. Habitat: Damp sites in moorland, 3000–3600 m. Voucher: Fries and Fries 2394 (EA).

***Carex bequaertii* De Wild.** – Life form: Herb. Habitat: Moist montane forest and bamboo thickets, 1950–3800 m. Voucher: Verdcourt 1769 (EA).

***Carex chlorosaccus* C.B.Clarke** – Life form: Herb. Habitat: Moist forest and riparian forest, 1300–3300 m. Voucher: Napper 715 (EA).

***Carex conferta* Hochst. ex A.Rich.** – Life form: Herb. Habitat: Upland moist forest and moorland, 2200–3650 m. Voucher: Robertson 7373(EA).

***Carex elgonensis* Nelmes** – Life form: Herb. Habitat: Bamboo thicket margins and afro-alpine stream banks, 2400–3650 m. Voucher: Hedberg 854 (EA).

***Carex johnstonii* Boeckeler** – Life form: Herb. Habitat: Upper parts of montane forest and bamboo zone, 2200–3300 m. Voucher: Musili et al. 422 (EA).

***Carex lycurus* K.Schum.** – Life form: Herb. Habitat: Stream banks in grassland and woodland, 1500–3350 m. Voucher: Verdcourt 1770 (EA).

***Carex monostachya* A.Rich.** – Life form: Herb. Habitat: Upper regions of bamboo zone and moorland, 2700–4500 m. Voucher: Musili et al 439 (EA).

***Carex peregrina* Link** – Life form: Herb. Habitat: Moist montane forest, 2300–3440 m. Voucher: Muasya et al. 050 (EA).

***Carex phragmitoides* Kük.** – Life form: Herb. Habitat: Bogs and marshes in montane forest, 2500–3100 m. Voucher: Taylor 1354 (EA).

**^E^Carex runssoroensis var. aberdarensis Kük.** – Life form: Herb. Habitat: Rocky grounds in moorland, 3000–4400 m. Vouchers: Hedberg 4327, Muasya et al. 048 (EA).

***Carex simensis* Hochst. ex A.Rich.** – Life form: Herb. Habitat: Swampy places in upland grassland and moorland, 1850–3900 m. Voucher: Brich 61/13 (EA).

***Carex vallis-rosetto* K.Schum.** – Life form: Herb. Habitat: Moist sites in forest and forest edges, 1000–3300 m. Voucher: Luke 15347 (EA).

***Cyperus afroalpinus* Lye** – Life form: Herb. Habitat: Open sites in montane forest and bamboo thickets, 1000–3000 m. Voucher: Haines 1969 (EA).

***Cyperus ajax* C.B.Clarke** – Life form: Herb. Habitat: Roadsides in upland forest and bush thickets, 950–2600 m. Voucher: Napper 1826 (EA).

***Cyperus aterrimus* Hochst. ex Steud.** – Life form: Herb. Habitat: Damp sites in upland montane forest, 1000–3350 m. Voucher: Brown 358 (EA).

***Cyperus cyperoides* (L.) Kuntze** – Life form: Herb. Habitat: Roadsides and forest clearings, 600–2400 m. Voucher: Kibui 43 (EA).

***Cyperus denudatus* L.f.** – Life form: Herb. Habitat: Damp grassland and riversides, 0–2000 m. Voucher: Kuchar 7820 (EA).

***Cyperus dereilema* Steud.** – Life form: Herb. Habitat: Moist montane forest and bamboo zone, 2100–3050 m. Voucher: Luke 15351 (EA).

***Cyperus dichroostachyus* Hochst. ex A.Rich.** – Life form: Herb. Habitat: Moist forest, 1200–2750 m. Voucher: Robertson 7372 (EA).

***Cyperus esculentus* L.** – Life form: Herb. Habitat: Swamps and wet grassland, 0–2200 m. Voucher: Faden 68/854 (EA).

***Cyperus karisimbiensis* (Cherm.) Kük.** – Life form: Herb. Habitat: Woodland, 1850–3000 m. Voucher: Fries and Fries 1037 (EA).

***Cyperus kerstenii* Boeckeler** – Life form: Herb. Habitat: Montane grassland and moorland, 2400–3600 m. Voucher: Beentje et al. 62 (EA).

***Cyperus papyrus* L.** – Life form: Herb. Habitat: Moist forest and wet grassy slopes, 1800–3120 m. Voucher: Wood 779 (EA).

***Cyperus rigidifolius* Steud.** – Life form: Herb. Habitat: Seasonally wet grassland and bushland, 1700–2800 m. Voucher: Robertson 7399 (EA).

***Cyperus rotundus* L.** – Life form: Herb. Habitat: Wet grassland, 0–2200 m. Voucher: Robertson 7368 (EA).

***Cyperus tomaiophyllus* K.Schum.** – Life form: Herb. Habitat: Moist forest and damp grassy slopes, 1800–3120 m. Voucher: Kuchar 12533 (EA).

***Eleocharis marginulata* Hochst. ex Steud.** – Life form: Herb. Habitat: Swampy grassland, 1500–2600 m. Voucher: Robertson 7369 (EA).

**Fimbristylis complanata subsp. keniaeensis (Kük.) Lye.** – Life form: Herb. Habitat: Swampy grassland, 1500–2700 m. Vouchers: Faden 67/771, Kabuye 378 (EA).

***Fimbristylis ovata* (Burm.f.) J.Kern** – Life form: Herb. Habitat: Wet wooded grassland, 0–2200 m. Voucher: Verdcourt 3253 (EA).

***Fuirena pubescens* (Poir) Kunth** – Life form: Herb. Habitat: Seasonally wet grassland, 850–2300 m. Voucher: Musili et al. 191(EA).

***Isolepis costata* Hochst. ex A.Rich.** – Life form: Herb. Habitat: Moist montane forest and stream banks, 1700–3500 m. Voucher: Taylor 1274 (EA).

***Isolepis fluitans* (L.) R.Br.** – Life form: Herb. Habitat: Bogs in moorland, 1200–3700 m. Voucher: Okwaro 34 (EA).

***Isolepis sepulcralis* Steud.** – Life form: Herb. Habitat: Upland wet grassland, 1800–2300 m. Voucher: Bogdan 1514a (EA).

***Isolepis setaceae* (L.) R.Br.** – Life form: Herb. Habitat: Wet montane grassland, 2400–3800 m. Voucher: Kuchar 8278 (EA).

**Kyllinga brevifolia var. lurida (Kük.) Beentje** – Life form: Herb. Habitat: Montane grassland and forest clearings, 1300–3300 m. Voucher: Hansen 754 (EA).

***Cyperus erecta* (Schumach.) Mattf. & Kük.** – Life form: Herb. Habitat: Wet depressions and swamps, 0–2000 m. Voucher: Musili et al. 244 (EA).

***Kyllinga odorata* Vahl.** – Life form: Herb. Habitat: Upland forest margins and woodland, 1300–3300 m. Voucher: Robertson 7370 (EA).

***Cyperus aethiops* Welw. ex Ridl.** – Life form: Herb. Habitat: Wet or swampy grassland, 900–2200 m. Voucher: Agnew et al. 8608 (EA).

***Cyperus elegantulus* Steud.** – Life form: Herb. Habitat: Wet grassland and moist forest margins, 1100–3050 m. Voucher: Napper 1488 (EA).

**Cyperus mundii var. uniceps (C.B.Clarke) Kük.** – Life form: Herb. Habitat: Wet grassland and swampy forest, 200–2300 m. Voucher: Verdcourt 427 (EA).

***Cyperus nigricans* Steud.** – Life form: Herb. Habitat: Marshy grounds and bogs in upland forest, 1700–3600 m. Voucher: Kuchar 9559 (EA).

***Cyperus nitidus* Lam.** – Life form: Herb. Habitat: Wetland and swamps edges, 1000–2150 m. Voucher: Bogdan 2894 (EA).

***Carex uhligii* K.Schum ex C.B.Clarke** – Life form: Herb. Habitat: Open sites in moist forest and upland grassland, 1050–2800 m. Voucher: Hansen 850 (EA).

***Carex spartea* Wahlenb.** – Life form: Herb. Habitat: Moist forest edges and wet upland grassland, 1650–2800 m. Voucher: Polhill 436 (EA).

**F29. Eriocaulaceae**

***Eriocaulon mesanthemoides* Ruhland** – Life form: Herb. Habitat: Stream-sides in montane grassland and moorland, 2400–3100 m. Voucher: Agnew et al. 8157 (EA).

***Eriocaulon schimperi* Körn. ex Ruhland** – Life form: Herb. Habitat: Wet montane grassland, 2000–3500 m. Voucher: Wood 773 (EA).

***Eriocaulon volkensii* Engl.** – Life form: Herb. Habitat: Montane grassland and moorland, 2500–3900 m. Voucher: Agnew 7226 (EA).

**F30. Hydrocharitaceae**

***Egeria densa* Planch.** – Life form: Herb. Habitat: Still or slow flowing freshwater, 360–2400 m. Voucher: Powys 373 (EA).

**F31. Hypoxidaceae**

***Hypoxis angustifolia* Lam.** – Life form: Herb. Habitat: Open woodland and forest margins, 0–3000 m. Voucher: Verdcourt 1965 (EA).

**Hypoxis kilimanjarica subsp. prostrata Ellen Holt & Staubo** – Life form: Herb. Habitat: Montane grassland, 2900–3500 m. Vouchers: Mabberlay 372, Beentje 2611 (EA).

**F32. Iridaceae**

***Aristea alata* Baker** – Life form: Herb. Habitat: Moist forest and forest edges, 1800–3500 m. Voucher: Mbale et al. 850 (EA).

***Aristea angolensis* Baker** – Life form: Herb. Habitat: Wet sites in grassland, 1750–2700 m. Voucher: Battiscombe 831 (EA).

***Dierama cupuliflorum* Klatt** – Life form: Herb. Habitat: Montane grassland, 2000–3900 m. Vouchers: Kuchar 12699, Beentje 2414 (EA).

***Gladiolus dalenii* Van Geel** – Life form: Herb. Habitat: Grassland with scattered trees, 300–3600 m. Voucher: Bally 8255B (EA).

***Gladiolus watsonioides* Baker** – Life form: Herb. Habitat: Forest clearings in montane forest, 2000–3800 m. Voucher: Lind 2901 (EA).

***Hesperantha petitiana* (A.Rich.) Baker** – Life form: Herb. Habitat: Rocky cliffs at subalpine grassland, 1800–3100 m. Voucher: Napier 1254 (EA).

***Romulea congoensis* Bég.** – Life form: Herb. Habitat: Montane grassland, 3300–4200 m. Voucher: Stephenson 383 (EA).

***Romulea fischeri* Pax** – Life form: Herb. Habitat: Upland grassland, 2150–4200 m. Voucher: Dyson 435 (EA).

**F33. Juncaceae**

***Juncus bufonius* L.** – Life form: Herb. Habitat: Wet places in upland grassland, 2400–2800 m. Voucher: Musili et al. 441 (EA).

**Juncus dregeanus subsp. bachitii (Hochst. ex Steud.) Hedberg.** – Life form: Herb. Habitat: Along streams in upland forest and moorland, 2100–3400 m. Vouchers: Hedberg 4328, Kuchar 12445 (EA).

***Juncus effusus* L.** – Life form: Herb. Habitat: Moist forest and streams banks, 1500–3300 m. Voucher: Kuchar 10330 (EA).

***Juncus oxycarpus* E.Mey ex Kunth** – Life form: Herb. Habitat: Marshy sites in grassland, 1500–3080 m. Voucher: Handa 007 (EA).

***Luzula abyssinica* Parl.** – Life form: Herb. Habitat: Damp sites in upper montane forest and moorland, 2000–4550 m. Voucher: Hedberg 1541 (EA).

***Luzula johnstonii* Buchenau** – Life form: Herb. Habitat: Shaded grounds in rainforest, 2400–4200 m. Voucher: Agnew et al. 7088 (EA).

**F34. Orchidaceae**

***Aerangis confusa* J.Stewart** – Life form: Herb. Habitat: Epiphytic in shaded trunks and branches in upland forest, 1600–2500 m. Voucher: Bally 8461 (EA).

***Aerangis thomsonii* (Rolfe) Schltr.** – Life form: Herb. Habitat: Epiphytic in shaded trunks and branches in upland forest, 1600–2600 m. Voucher: SK 0260 (EA, HIB).

***Angraecum chamaeanthus* Schltr.** – Life form: Herb. Habitat: Epiphytic in upland montane forest, 1600–2400 m. Voucher: Smart 27 (EA).

***Angraecum conchiferum* Lindl.** – Life form: Herb. Habitat: Epiphytic in montane forest, 1250–2400 m. Voucher: Archer 20/9/1959 (EA).

***Angraecum humile* Summerh.** – Life form: Herb. Habitat: Epiphytic in moist montane forest and riparian forest, 1650–2500 m. Voucher: Someren 413 (EA).

***Angraecum sacciferum* Lindl.** – Life form: Herb. Habitat: Epiphytic in upland moist forest, 900–2200 m. Voucher: Bytebier 505 (EA).

***Bulbophyllum sandersonii* (Hook.f.) Rchb.f.** – Life form: Herb. Habitat: Epiphytic in shades in forest, 200–2700 m. Voucher: Cameron 14863 (EA).

***Calanthe sylvatica* (Thouars) Lindl.** – Life form: Herb. Habitat: Moist forest floors and near streams, 900–3000 m. Voucher: Gardner 1411 (EA).

***Cynorkis anacamptoides* Kraenzl.** – Life form: Herb. Habitat: Upland grassland and moorland, 1050–3350 m. Voucher: Verdcourt 434 (EA).

***Cyrtorchis arcuata* (Lindl.) Schltr.** – Life form: Herb. Habitat: Epiphytic in woodland and open forest, 0–3300 m. Vouchers: Gardner 2554, Padwa 59 (EA).

***Diaphananthe rohrii* (Rchb.f.) Summerh.** – Life form: Herb. Habitat: Epiphytic in montane forest, 2100–3000 m. Voucher: Smart 22 (EA).

**Disa fragrans subsp. deckenii (Rchb.f.) H.P.Linder** – Life form: Herb. Habitat: Open sites and edges of montane forest up to the moorland, 2350–3700 m. Vouchers: Kokwaro 1929, Agnew et al. 8186 (EA).

***Disa stairsii* Kraenzl.** – Life form: Herb. Habitat: Upland grassy swamps and damp sites in moorland, 2100–3750 m. Voucher: Kokwaro 3249 (EA).

***Disperis dicerochila* Summerh.** – Life form: Herb. Habitat: Upland moist forest, 1650–2600 m. Voucher: Williams 15379 (EA).

***Disperis kilimanjarica* Rendle** – Life form: Herb. Habitat: Shaded grounds in evergreen forest, 2100–3000 m. Voucher: SK 0133 (EA, HIB).

***Epipactis africana* Rendle** – Life form: Herb. Habitat: Montane evergreen forest and bamboo thickets, 2330–3750 m. Voucher: Verdcourt et al. 3022 (EA).

***Habenaria attenuata* Hook.f.** – Life form: Herb. Habitat: Upland grassland and moorland, 2700–4000 m. Voucher: Davis 7A (EA).

***Habenaria decorata* Hochst. ex A.Rich.** – Life form: Herb. Habitat: Rocky sites in moorland, 2200–3300 m. Voucher: Ward s.n (EA).

***Habenaria keniensis* Summerh.** – Life form: Herb. Habitat: Upland rainforest, 1950–2950 m. Voucher: Belcher 151/54 (EA).

***Habenaria macrantha* Hochst. ex A.Rich.** – Life form: Herb. Habitat: Upland grassland and moorland, 2550–3000 m. Voucher: Dale 2842 (EA).

***Habenaria petitiana* (A.Rich) T.Durand & Schinz** – Life form: Herb. Habitat: Forest edges and bushland, 1500–3300 m. Voucher: William 15380 (EA).

***Habenaria schimperiana* Hochst. ex A.Rich.** – Life form: Herb. Habitat: Swamps and wet grassland, 1250–2800 m. Voucher: Dale 2876 (EA).

***Habenaria tweedieae* Summerh.** – Life form: Herb. Habitat: In grass on rocky hills, 1950–2600 m. Voucher: Cunningham 44 (EA).

***Habenaria vaginata* A.Rich.** – Life form: Herb. Habitat: Wet sites in grassland, 1300–3000 m. Voucher: Dale 2354 (EA).

***Holothrix brongniartiana* Rchb.f.** – Life form: Herb. Habitat: Upland grassland, 1200–3500 m. Voucher: Williams 12362 (EA).

***Liparis deistelii* Schltr.** – Life form: Herb. Habitat: Epiphytic on fallen trees in shades near rivers, 1700–2750 m. Voucher: Dale 2861 (EA).

**Polystachya caespitifica subsp. latilabris (Summerh.) P.J.Cribb & Podz.** – Life form: Herb. Habitat: Epiphytic in montane forest, 1800–2200 m. Voucher: Lucs et al. 274 (EA).

***Polystachya cultriformis* (Thouars) Lindl. ex Spreng** – Life form: Herb. Habitat: Moist montane forest, 1800–2200 m. Vouchers: SK 0140, SK 00227 (EA, HIB).

***Polystachya heckmanniana* Kraenzl.** – Life form: Herb. Habitat: Upland moist forest, 1200–2000 m. Voucher: Turner 3231 (EA).

***Polystachya transvaalensis* Schltr.** – Life form: Herb. Habitat: Moist forest, 1200–2900 m. Vouchers: Turner 3239 & 2385 (EA).

***Satyrium crassicaule* Rendle** – Life form: Herb. Habitat: Wet grassland and swamps, 1000–3150 m. Voucher: Bytebier 500 (EA).

***Satyrium macrophyllum* Lindl.** – Life form: Herb. Habitat: Upland wet grassland, 1200–3150 m. Voucher: Dale 75 (EA).

***Satyrium schimperi* Hochst. ex A.Rich.** – Life form: Herb. Habitat: Upland grassland, 2100–3200 m. Voucher: Pierce 2696 (EA).

***Satyrium volkensii* Schltr.** – Life form: Herb. Habitat: Upland grassland and bushland, 1050–2400 m. Voucher: Piers 143/51 (EA).

**F35. Poaceae**

***Acritochaete volkensii* Pilg.** – Life form: Herb. Habitat: Shaded grounds in upland forest and bamboo thicket, 2300–3300 m. Voucher: Agnew et al. 8180 (EA).

***Agrostis gracilifolia* C.E.Hubb.** – Life form: Herb. Habitat: Wet places in upland grassland and moorland, 2800–3980 m. Voucher: Beentje 3269(EA).

***Agrostis keniensis* Pilg.** – Life form: Herb. Habitat: Along streams and moist forest margins, 2200–3000 m. Voucher: Turner 1569 (EA).

***Agrostis kilimandscharica* Mez** – Life form: Herb. Habitat: Upland forest clearings and margins up to bamboo zone, 2000–4000 m. Voucher: Agnew et al. 8134 (EA).

***Agrostis producta* Pilg.** – Life form: Herb. Habitat: Upland grassland and moorland, 2400–4000 m. Voucher: Robertson 2162 (EA).

***Agrostis trachyphylla* Pilg.** – Life form: Herb. Habitat: Wet places in upland grassland and moorland, 3500–4900 m. Voucher: AfroAlp II team 1063 (EA).

***Agrostis volkensii* Stapf** – Life form: Herb. Habitat: Upland grassland and moorland, 3000–3900 m. Voucher: AfroAlp II team 0974 (EA).

***Aira caryophyllea* L.** – Life form: Herb. Habitat: Rocky soils in upland grassland and moorland, 2000–4500 m. Voucher: Rauh 407 (EA).

***Andropogon abyssinicus* R.Br. ex Fresen.** – Life form: Herb. Habitat: Upland grassland, 200–3100 m. Voucher: Kuchar 12501 (EA).

***Andropogon amethystinus* Steud.** – Life form: Herb. Habitat: Open or clearings in montane forest and moorland, 1400–4000 m. Voucher: Grant 1238 (EA).

***Andropogon chrysostachyus* Steud.** – Life form: Herb. Habitat: Moist upland grassland and evergreen forest, 2100–3300 m. Voucher: Napper 745 (EA).

***Andropogon distachyos* L.** – Life form: Herb. Habitat: Upland dry grassland, 1700–3000 m. Voucher: Kuchar 12321 (EA).

***Andropogon lima* (Hack.) Stapf** – Life form: Herb. Habitat: Montane grassland and dry moorland, 2400–4000 m. Voucher: Rauh 522 (EA).

***Anthoxanthum nivale* K.Schum.** – Life form: Herb. Habitat: Upland moist grassland and moorland, 2500–3980 m. Voucher: Kuchar 12739(EA).

***Aristida adoensis* Hochst. ex A.Rich.** – Life form: Herb. Habitat: Deciduous bushland, 1300–2300 m. Voucher: Robertson 332 (EA).

***Aristida junciformis* Trin. & Rupr.** – Life form: Herb. Habitat: Dry rocky hilltops, 400–2100 m. Voucher: Bogdan 4163 (EA).

***Avena fatua* L.** – Life form: Herb. Habitat: Upland grassland, 2100–2400 m. Voucher: Ghosh 4 (EA).

***Avena sterilis* L.** – Life form: Herb. Habitat: Upland grassland, 2600–2600 m. Voucher: Someren 13/12/1956 (EA).

***Bothriochloa insculpta* (A.Rich.) A.Camus** – Life form: Herb. Habitat: Grassland, 0–2250 m. Voucher: Hansen 800 (EA).

***Brachypodium flexum* Nees** – Life form: Herb. Habitat: Moist forest and bamboo thickets, 2000–3000 m. Voucher: Kuchar and Msafiri 5422 (EA).

***Briza maxima* L.** – Life form: Herb. Habitat: Roadsides in forest, 2400–2700 m. Voucher: Edwards 2833/6 (EA).

***Bromus catharticus* Vahl** – Life form: Herb. Habitat: Disturbed grounds and roadsides, 2300–2700 m. Vouchers: Greenway 10399 & 9574 (EA).

***Bromus diandrus* Roth** – Life form: Herb. Habitat: Disturbed grounds and roadsides, 2300–3000 m. Voucher: Muchiri 580 (EA).

***Bromus leptoclados* Nees** – Life form: Herb. Habitat: Upland grassland and forest clearings, 2300–4300 m. Voucher: Bogdan 2646 (EA).

***Calamagrostis epigejos* (L.) Roth** – Life form: Herb. Habitat: Upland grassland and forest clearings, 2000–3000 m. Voucher: Kokwaro 3340 (EA).

***Calamagrostis hedbergii* Melderis** – Life form: Herb. Habitat: Rocky moorland, 3500–4250 m. Voucher: Hedberg 1810 (EA).

***Chloris virgata* Sw.** – Life form: Herb. Habitat: Scattered-tree grassland and bushland, 10–2120 m. Voucher: Robertson 2218 (EA).

***Coelachne friesiorum* C.E.Hubb.** – Life form: Herb. Habitat: Moist montane forest, 3000–3000 m. Voucher: Fries 2407 (EA).

***Colpodium hedbergii* (Melderis) Tzvelev** – Life form: Herb. Habitat: Moorland, 3580–4000 m. Voucher: Hanid 157 (EA).

***Cymbopogon nardus* (L.) Rendle** – Life form: Herb. Habitat: Upland grassland, 1000–3000 m. Voucher: Waiganjo 39 (EA).

***Cynodon dactylon* (L.) Pers.** – Life form: Herb. Habitat: Roadsides in grassland, 0–2000 m. Voucher: Mbevi 194 (EA).

***Deschampsia cespitosa* (L.) P.Beauv.** – Life form: Herb. Habitat: Damp places in moorland, 2900–4000 m. Voucher: Polhill 12035 (EA).

***Deschampsia flexuosa* (L.) Trin.** – Life form: Herb. Habitat: Upland grassland and moorland, 2600–3920 m. Voucher: Agnew et al. 8135A (EA).

***Digitaria abyssinica* (A.Rich.) Stapf** – Life form: Herb. Habitat: Ruderal sites in upland grassland, 0–3000 m. Voucher: Turner 1564 (EA).

***Digitaria diagonalis* (Nees) Stapf** – Life form: Herb. Habitat: Grassland, 0–2640 m. Voucher: Mbale 1024 (EA).

***Digitaria gazensis* Rendle** – Life form: Herb. Habitat: Upland grassland, 1200–2600 m. Voucher: Barney 1031 (EA).

***Digitaria longiflora* (Retz.) Pers.** – Life form: Herb. Habitat: Deciduous bushland, 0–2300 m. Voucher: Robertson 2131 (EA).

***Digitaria thouaresiana* (Flüggé) A.Camus** – Life form: Herb. Habitat: Moist forest, 0–2400 m. Voucher: Kerfoot 1359 (EA).

***Digitaria velutina* (Forssk.) P.Beauv.** – Life form: Herb. Habitat: Roadsides and other disturbed places, 0–2300 m. Voucher: Robertson 7337 (EA).

***Ehrharta erecta* Lam.** – Life form: Herb. Habitat: Upland forest glades and margins, 1500–2700 m. Voucher: Robertson 7350 (EA).

***Eleusine jaegeri* Pilg.** – Life form: Herb. Habitat: Upland grassland and open sites in forest, 1800–3300 m. Voucher: Turner 1573 (EA).

***^E^Eragrostis amanda* Clayton** – Life form: Herb. Habitat: Glades in bamboo thickets, 2400–2900 m. Voucher: Dyson 442 (EA).

***Eragrostis chalarothyrsos* C.E.Hubb.** – Life form: Herb. Habitat: Swampy grassland, 1100–2500 m. Voucher: Lepelley 5 (EA).

***Eragrostis olivacea* K.Schum.** – Life form: Herb. Habitat: Rocky sites in grassland, 1300–3300 m. Voucher: Napper 1699 (EA).

***Eragrostis patula* (Kunth) Steud.** – Life form: Herb. Habitat: Roadsides and disturbed sites, 0–2800 m. Voucher: Verdcourt 3642 (EA).

***Eragrostis schweinfurthii* Chiov.** – Life form: Herb. Habitat: Upland evergreen forest, 1300–3000 m. Voucher: Beentje 3234 (EA).

***Eragrostis tef* (Zucc.) Trotter** – Life form: Herb. Habitat: Roadsides and disturbed areas, 750–2500 m. Voucher: Frazer 195 (EA).

***Exotheca abyssinica* (Hochst. ex A.Rich.) Andersson** – Life form: Herb. Habitat: Upland grassland and moorland, 2000–4000 m. Voucher: Coe 771 (EA).

***Festuca africana* (Hack.) Clayton** – Life form: Herb. Habitat: Shaded sites in upland forest and bamboo thickets, 2000–3000 m. Voucher: Bogdan 2832 (EA).

***Festuca arundinacea* Schreb.** – Life form: Herb. Habitat: Along stream banks in upland forests, 2300–3090 m. Voucher: Bogdan 4760 (EA).

***Festuca camusiana* St.-Yves** – Life form: Herb. Habitat: Upland forest and bamboo thicket, 2100–3500 m. Voucher: Kerfoot 1428 (EA).

***Festuca costata* Nees** – Life form: Herb. Habitat: Upland grassland, 2400–3000 m. Voucher: Schelpe 2684 (EA).

***Festuca mekiste* Clayton** – Life form: Herb. Habitat: Forest edges and open sites in upland forest, 2300–3480 m. Voucher: Bogdan 3274 (EA).

***Festuca pilgeri* St.-Yves** – Life form: Herb. Habitat: Moorland, 2700–4250 m. Voucher: Agnew and Timberlake 11165 (EA).

***Festuca simensis* Hochst. ex A.Rich.** – Life form: Herb. Habitat: Shaded places in upland forest, 2000–3300 m. Voucher: Robertson 3935 (EA).

***Harpachne schimperi* A.Rich.** – Life form: Herb. Habitat: Grassland and open bushland, 500–3000 m. Voucher: Faden et al. 74/613 (EA).

***Helictotrichon elongatum* (Hochst. ex A.Rich.) C.E.Hubb.** – Life form: Herb. Habitat: Upland forest edges and grassland, 1800–3800 m. Voucher: Kuchar and Msafiri 5407 (EA).

***Helictotrichon milanjianum* (Rendle) C.E.Hubb.** – Life form: Herb. Habitat: Moist shaded places in montane forest and bamboo thicket, 2300–3500 m. Voucher: Kerfoot 1424 (EA).

***Helictotrichon umbrosum* (Hochst. ex Steud.) C.E.Hubb.** – Life form: Herb. Habitat: Upland grassland and margins of bamboo thickets, 1850–4000 m. Voucher: Peacock 58 (EA).

***Hyparrhenia mobukensis* (Chiov.) Chiov.** – Life form: Herb. Habitat: Margins of montane evergreen forest and bamboo forest, 2500–3300 m. Voucher: Kerfoot 1353 (EA).

***Hyparrhenia tamba* (Hochst. ex Steud.) Andersson ex Stapf** – Life form: Herb. Habitat: Upland grassland, 2000–3300 m. Voucher: Kerfoot 431 (EA).

***Hyparrhenia umbrosa* (Hochst.) Andersson ex Clayton** – Life form: Herb. Habitat: Roadsides in lower montane forest, 1500–2300 m. Voucher: Glover et al. 1424 (EA).

***Koeleria capensis* Nees** – Life form: Herb. Habitat: Upland grassland and moorland, 1800–5300 m. Voucher: Grout 1247 (EA).

***Leersia denudata* Launert** – Life form: Herb. Habitat: Swampy grassland, 1500–2300 m. Voucher: Faden 74/671(EA).

***Lolium temulentum* L.** – Life form: Herb. Habitat: Grazed pasture in moist forest, 1900–2300 m. Voucher: Lundin 5097 (EA).

***Oplismenus hirtellus* (L.) P.Beauv.** – Life form: Herb. Habitat: Shaded grounds in forest, 0–2500 m. Voucher: Nattrass 716 (EA).

***Oplismenus undulatifolius* (Ard.) Roem & Schult.** – Life form: Herb. Habitat: Shaded grounds in forest, 1400–2500 m. Voucher: Bogdan 378 (EA).

***Panicum calvum* Stapf** – Life form: Herb. Habitat: Shades in forest and forest margins, 1000–3000 m. Voucher: Davidse 7050 (EA).

***Panicum hymeniochilum* Nees** – Life form: Herb. Habitat: River banks and swamps, 700–3120 m. Voucher: Kabuye & Wood 100 (EA).

***Panicum monticola* Hook.f.** – Life form: Herb. Habitat: Shaded forest floors, 600–2600 m. Voucher: Napper et al. 1719 (EA).

***Panicum pusillum* Hook.f.** – Life form: Herb. Habitat: Montane grassland or bushland, 1300–3300 m. Voucher: Bogdan 4881 (EA).

***Panicum subalbidum* Kunth** – Life form: Herb. Habitat: Along rivers and swamps, 200–3400 m. Voucher: Kuchar 7835 (EA).

*Paspalum notatum Flüggé – Life form: Exotic herb. Habitat: Grassland, 500–2100 m. Voucher: Hindorf 693 (EA).

***Pennisetum clandestinum* Hochst. ex Chiov.** – Life form: Herb. Habitat: Upland grassland, 1400–3300 m. Voucher: Mungai 65 (EA).

***Pennisetum hohenackeri* Hochst. ex Steud.** – Life form: Herb. Habitat: Upland grassland, 1100–2400 m. Voucher: Robertson 7322 (EA).

***Pennisetum riparium* Hochst. ex A.Rich.** – Life form: Herb. Habitat: Swamps, 1400–2700 m. Voucher: Bogdan 3514 (EA).

***Pennisetum sphacelatum* (Nees) T.Durand & Schinz** – Life form: Herb. Habitat: Upland grassland and evergreen forests, 1500–3200 m. Voucher: Bally 1177 (EA).

***Pennisetum thunbergii* Kunth** – Life form: Herb. Habitat: Upland pasture and roadsides in moist forest, 1500–3500 m. Voucher: Kerfoot 1422 (EA).

***Pennisetum trachyphyllum* Pilg.** – Life form: Herb. Habitat: Moist forest often along paths and glades, 1000–2500 m. Voucher: Napper 1718 (EA).

***Pentameris borussica* (K.Schum.) Galley & H.P.Linder** – Life form: Herb. Habitat: Upland grassland and moorland, 3000–4680 m. Voucher: Kuchar 12524 (EA).

***Pentameris pictigluma* (Steud.) Galley & H.P.Linder** – Life form: Herb. Habitat: Upland grassland and moorland, 2600–4500 m. Voucher: Hedberg 1538 (EA).

***Phalaris arundinacea* L.** – Life form: Herb. Habitat: Stream banks and swamps margins, 1950–3000 m. Voucher: Verdcourt 876 (EA).

***Poa annua* L.** – Life form: Herb. Habitat: Disturbed grounds in upland forest, 2000–3550 m. Voucher: Vorontsova 767 (EA).

***Poa leptoclada* Hochst. ex A.Rich.** – Life form: Herb. Habitat: Upland forest margins and grassland, 1800–4750 m. Voucher: Vorontsova 768 (EA).

***Poa schimperiana* Hochst. ex A.Rich.** – Life form: Herb. Habitat: Upland grassland and moorland, 1980–4200 m. Voucher: Hedberg 1595 (EA).

***Poecilostachys oplismenoides* (Hack.) Clayton** – Life form: Herb. Habitat: Shaded places in evergreen forest, 1000–2500 m. Voucher: Bogdan 1504 (EA).

***Polypogon schimperianus* (Hochst. ex Steud.) Cope** – Life form: Herb. Habitat: Along rivers and moist places in upland grassland and moorland, 1500–4000 m. Voucher: Greenway 10400 (EA).

***Pseudechinolaena polystachya* (Humb., Bonpl. & Kunth) Stapf** – Life form: Herb. Habitat: Upland moist forest often in shady grounds, 1000–2500 m. Voucher: Faden 67374 (EA).

***Setaria atrata* Hack. ex Engl.** – Life form: Herb. Habitat: Swamps, 2000–2600 m. Voucher: Bogdan 2787 (EA).

***Setaria megaphylla* (Steud.) T.Durand & Schinz** – Life form: Herb. Habitat: Shaded places in forest, 200–2350 m. Voucher: SK 0246 (EA, HIB).

***Setaria sphacelata* (Schumach.) Stapf & C.E.Hubb. ex Moss** – Life form: Herb. Habitat: Wooded grassland, 0–3300 m. Voucher: Robertson 7312 (EA).

***Snowdenia polystachya* (Fresen.) Pilg.** – Life form: Herb. Habitat: Upland grassland, 2100–2700 m. Voucher: Luke et al. 16120 (EA).

***Sporobolus africanus* (Poir.) Robyns & Tournay** – Life form: Herb. Habitat: Grazed grassland, 1300–2640 m. Voucher: Robertson 7312 (EA).

***Sporobolus agrostoides* Chiov.** – Life form: Herb. Habitat: Shades in upland evergreen forest, 1300–2800 m. Voucher: Robertson 7348 (EA).

***Sporobolus fimbriatus* (Trin.) Nees** – Life form: Herb. Habitat: Open deciduous bushland, 30–2000 m. Voucher: Mwadime et al. 30 (EA).

***Sporobolus olivaceus* Napper** – Life form: Herb. Habitat: Upland grassland and moorland, 2010–4000 m. Voucher: Turner 1568 (EA).

***Sporobolus quadratus* Clayton** – Life form: Herb. Habitat: Grassland and disturbed sites, 2200–3000 m. Voucher: Turner 1571 (EA).

***Sporobolus spicatus* (Vahl) Kunth** – Life form: Herb. Habitat: Grassland and open bushland, 0–2600 m. Voucher: Hammilton 384 (EA).

***Stipa dregeana* Steud.** – Life form: Herb. Habitat: Moist evergreen forest, 1940–2770 m. Voucher: Robertson 7342 (EA).

***Streblochaete longiarista* (A.Rich) Pilg.** – Life form: Herb. Habitat: Undergrowth in moist forest and bamboo thickets, 1500–3280 m. Voucher: Magogo 1529 (EA).

***Themeda triandra* Forssk.** – Life form: Herb. Habitat: Open deciduous bushland, 0–3200 m. Voucher: Turner 1577 (EA).

***Vulpia bromoides* (L.) Gray** – Life form: Herb. Habitat: Rocky grounds in upland grassland, 2500–3500 m. Voucher: Kuchar 12326 (EA).

***Yushania alpina* (K.Schum.) W.C.Lin** – Life form: Herb or subshrub. Habitat: Upland moist bush thickets and forested slopes, 2300–3300 m. Voucher: Fries 2565 (EA).

**F36. Potamogetonaceae**

***Potamogeton pusillus* L.** – Life form: Herb. Habitat: Aquatic in slow flowing streams, 600–2000 m. Voucher: Colgahoum (EA).

***Potamogeton richardii* Solms** – Life form: Herb. Habitat: In water pools and streams, 1150–3450 m. Voucher: Verdcourt & Steele 913 (EA).

**F37. Smilacaceae**

***Smilax aspera* L.** – Life form: Shrub. Habitat: Moist forest and associated bushland, 1450–2745 m. Voucher: Luke 14245 (EA).

**F38. Xanthorrhoeaceae**

***Aloe kedongensis* Reynolds** – Life form: Shrub. Habitat: Rocky sites in open woodland, 1825–2300 m. Voucher: Robertson 1747 (EA).

***Aloe ngongensis* Christian** – Life form: Shrub. Habitat: Open deciduous woodland and forest margins, 1370–1900 m. Voucher: Perdue and Kibuwa 8068 (EA).

***Aloe nyeriensis* Christian & I.Verd.** – Life form: Shrub. Habitat: Upland open bushland, 1760–2100 m. Voucher: Napier 2186 (EA).

***Kniphofia thomsonii* Baker** – Life form: Herb. Habitat: Along stream and swampy sites in forest, 1850–3960 m. Voucher: SK 0060 (EA, HIB).

**F39. Xyridaceae**

***Xyris capensis* Thunb.** – Life form: Herb. Habitat: Bogs and marshes in montane forest, 1100–3000 m. Voucher: Gilbert 4875 (EA).

### Part 5. Magnoliopsida

**F40. Acanthaceae**

***Asystasia lorata* Ensermu** – Life form: Herb. Habitat: Wooded grassland and bushland, 1400–2000 m. Voucher: Verdcourt 3553 (EA).

***Barleria ventricosa* Hochst. ex Nees** – Life form: Herb or subshrub. Habitat: Upland dry woodland and thickets, 1500–3590 m. Voucher: Napier 10542 (EA).

***Crossandra tridentata* Lindau** – Life form: Herb. Habitat: Upland rainforest in shaded places, 700–2700 m. Voucher: Napier 2711 (EA).

***Dicliptera laxata* C.B.Clarke** – Life form: Herb or subshrub. Habitat: Moist montane forest and forest margins, 1300–2800 m. Voucher: Luke 9586 (EA).

**Dicliptera maculata subsp. usambarica (Lindau) I.Darbysh.** – Life form: Herb. Habitat: Moist forest, 1150–3050 m. Vouchers: Napier 620, Luke 8243 (EA).

**Dyschoriste keniensis subsp. keniensis Malombe, Mwachala & Vollesen** – Life form: Herb or subshrub. Habitat: Wooded grassland and bushland, 900–2500 m. Voucher: Perdue and Kibuwa 8182 (EA).

***Dyschoriste nagchana* (Nees) Bennet** – Life form: Herb. Habitat: Wet grasslands and swamps, 0–1800 m. Voucher: Faden 74/697 (EA).

***Dyschoriste radicans* (Hochst. ex A.Rich) Nees** – Life form: Herb or subshrub. Habitat: Upland bushland and grassland, 900–2500 m. Voucher: Mailnnes 69 (EA).

***Hypoestes aristata* (Vahl) Roem. & Schult.** – Life form: Herb or subshrub. Habitat: Forest margin, 200–3000 m. Voucher: Napier 619 (EA).

***Hypoestes forskaolii* (Vahl) R.Br.** – Life form: Herb or subshrub. Habitat: Dry grassland and bushland, 0–3000 m. Voucher: Nattrass 646 (EA).

***Hypoestes triflora* (Forssk.) Roem. & Schult.** – Life form: Herb. Habitat: Forest margins and grassland, 900–3200 m. Voucher: Albrechtren 2618 (EA).

***Isoglossa gregoryi* (S.Moore) Lindau** – Life form: Herb. Habitat: Forest margins and grassland, 1700–2900 m. Voucher: Chandler 2221 (EA).

***Isoglossa lactea* Lindau ex Engl.** – Life form: Herb or subshrub. Habitat: Montane forest, 1250–2350 m. Voucher: Taylor 1032 (EA).

***Isoglossa substrobilina* C.B.Clarke** – Life form: Herb or subshrub. Habitat: Undergrowth in montane forest, 1750–2600 m. Voucher: Balbo 828 (EA).

***Justicia striata* (Klotzsch) Bullock** – Life form: Herb. Habitat: Grassland and bushland, 200–2600 m. Voucher: Verdcourt 2651 (EA).

***Justicia unyorensis* S.Moore** – Life form: Herb. Habitat: Moist woodland and bushland, 1150–3100 m. Voucher: Kerfoot 1389 (EA).

***Mimulopsis alpina* Chiov.** – Life form: Woody herb or shrub. Habitat: Montane evergreen forest, 1900–3300 m. Voucher: Dale 2154 (EA).

***Mimulopsis solmsii* Schweinf.** – Life form: Herb. Habitat: Montane evergreen forest, 1200–2700 m. Voucher: Jackson (EA).

***Phaulopsis imbricata* (Forssk.) Sweet** – Life form: Herb. Habitat: Forest edges and riverine forest, 1500–2970 m. Voucher: Faden 74/608 (EA).

***Rhinacanthus ndorensis* Schweinf. ex Engl.** – Life form: Herb or subshrub. Habitat: Upland grassland and open woodland, 1700–2150 m. Voucher: Napier 2579 (EA).

***Thunbergia gibsonii* S.Moore** – Life form: Herbaceous climber. Habitat: Montane grassland and bushland, 1700–3000 m. Voucher: Fries 2815 (EA).

***Thunbergia alata* Bojer ex Sims** – Life form: Herbaceous climber. Habitat: Wet bushland, 100–3000 m. Voucher: SK 0018 (EA, HIB).

***Thunbergia fischeri* Engl.** – Life form: Herb. Habitat: Upland grassland, 1200–2500 m. Voucher: Napier 1798 (EA).

***Thunbergia reniformis* Vollesen** – Life form: Herbaceous climber. Habitat: Grassland, 1800–2400 m. Voucher: Someren 5057 (EA).

**F41. Adoxaceae**

***Sambucus africana* Standl.** – Life form: Herb or shrub. Habitat: Roadsides in bamboo zone and montane forest, 1750–3370 m. Voucher: SK 0046 (EA, HIB).

***Sambucus ebulus* L.** – Life form: Herb. Habitat: Clearings and edges of moist bamboo thickets, 2320–3370 m. Voucher: Williams 1305 (EA).

**F42. Amaranthaceae**

***Achyranthes aspera* L.** – Life form: Herb. Habitat: Moist forest and open grassland, 0–3080 m. Voucher: SK 0206 (EA, HIB).

***Aerva lanata* (L.) Juss.** – Life form: Herb. Habitat: Moist forest edges and bushland, 0–2200 m. Voucher: Kirika et al. 152 (EA).

***Alternanthera caracasana* Kunth** – Life form: Herb. Habitat: Roadsides and forest edges, 0–2020 m. Voucher: Kokwaro 2795 (EA).

***Alternanthera pungens* Kunth** – Life form: Herb. Habitat: Roadsides and river banks, 0–2020 m. Voucher: Bally 4609 (EA).

***Amaranthus graecizans* L.** – Life form: Herb. Habitat: Grassland, 950–2900 m. Voucher: Hansen 85 (EA).

***Amaranthus hybridus* L.** – Life form: Herb. Habitat: Roadsides and forest edges, 1500–2600 m. Voucher: Mbevi 182 (EA).

***Celosia anthelminthica* Asch.** – Life form: Herb or subshrub. Habitat: Moist forest margins and clearings, 500–2300 m. Voucher: Someren 810 (EA).

***Chenopodium album* L.** – Life form: Herb. Habitat: Roadsides in moist forest, 1650–2600 m. Voucher: Lochhead 610 (EA).

***Chenopodium carinatum* R.Br.** – Life form: Herb. Habitat: Roadsides and pasture grounds, 900–2100 m. Voucher: Verdcourt 391 (EA).

***Chenopodium fasciculosum* Aellen** – Life form: Herb. Habitat: Roadsides in moist forest, 1310–2600 m. Voucher: Bogdan 4169 (EA).

***Chenopodium murale* L.** – Life form: Herb. Habitat: Grazing areas in lower parts of montane forest, 1070–2750 m. Voucher: Mungai 57 (EA).

***Chenopodium opulifolium* Schrad. ex W.D.J.Koch & Ziz** – Life form: Herb. Habitat: Roadsides and forest edges, 760–2100 m. Voucher: Bally 14439 (EA).

***Cyathula cylindrica* Moq.** – Life form: Herb or subshrub. Habitat: Moist forest and open rocky bushland, 1300–3240 m. Voucher: SK 0258 (EA, HIB).

***Cyathula uncinulata* (Schrad.) Schinz** – Life form: Herb. Habitat: Grassland and open bushland, 900–2730 m. Voucher: Faden 74/606 (EA).

***Dysphania schraderiana* (Schult.) Mosyakin & Clemants** – Life form: Herb. Habitat: Roadsides and forest margins, 1600–2300 m. Voucher: Fries 138 (EA).

***Gomphrena celosioides* Mart.** – Life form: Herb. Habitat: Forest edges and roadsides, 0–2150 m. Voucher: SK 0156 (EA, HIB).

***Pupalia lappacea* (L.) Juss.** – Life form: Herb. Habitat: Open sites in forest and forest edges, 10–2060 m. Voucher: Faden 74/664 (EA).

**F43. Anacardiaceae**

***Rhus longipes* Engl.** – Life form: Shrub or small tree. Habitat: Evergreen bushland and forest margins, 1000–2400 m. Vouchers: Jackson 312, Gardner 3623 (EA).

***Sclerocarya birrea* (A.Rich) Hochst.** – Life form: Tree. Habitat: Roadsides and bushland, 800–1800 m. Voucher: SK 0167 (EA, HIB).

***Searsia natalensis* (Bernh. ex C.Krauss) F.A.Barkley** – Life form: Tree. Habitat: Evergreen bushland and forest edges, 1–3000 m. Voucher: SK 0099 (EA, HIB).

**F44. Apiaceae**

***Afroligusticum aculeolatum* (Engl.) P.J.D.Winter** – Life form: Herb. Habitat: Bushland and moist montane forest, 1360–3030 m. Vouchers: Albrechtsen 6744, Scheffler 277 (EA).

***Afroligusticum elgonense* (H.Wolff) P.J.D.Winter** – Life form: Herb. Habitat: Wet sites and forest margins in montane forest, 1600–3600 m. Voucher: SK 0175 (EA, HIB).

***Afroligusticum linderi* (C.Norman) P.J.D.Winter** – Life form: Herb. Habitat: Montane grassland, 1860–3422 m. Voucher: Verdcourt & Polhill 7 (EA).

**^E^*Afrosciadium englerianum* (H.Wolff) P.J.D.Winter** – Life form: Herb. Habitat: Tussocky grounds in moorland, 3400–3750 m. Voucher: Townsend 2406 (EA).

**^E^Afrosciadium friesiorum var. friesiorum (H.Wolff) P.J.D.Winter** – Life form: Herb. Habitat: Wet grassy sites in bamboo and ericaceous zone, 2950–4260 m. Voucher: SK 0112 (EA, HIB).

**^E^Afrosciadium friesiorum var. bipinnatum (C.C.Towns.) P.J.D.Winter** – Life form: Herb. Habitat: Damp sites in moorland, 3000–3824 m. Voucher: Williams 1296 (EA).

***Afrosciadium kerstenii* (Engl.) P.J.D.Winter** – Life form: Herb. Habitat: Open places in bamboo zone, 2550–4300 m. Voucher: Verdcourt 2055 (EA).

***Agrocharis incognita* (C.Norman) Heywood & Jury** – Life form: Herb. Habitat: Montane forest margins, 900–3600 m. Vouchers: SK 0223, SK 0180 (EA, HIB).

***Agrocharis melanantha* Hochst.** – Life form: Herb. Habitat: Upland moist forest, 1520–3580 m. Voucher: RV 648 (EA).

***Alepidea peduncularis* Steud. ex A.Rich.** – Life form: Herb. Habitat: Grassland and open grounds in montane forest, 1050–3600 m. Voucher: SK 0109 (EA, HIB).

***Anthriscus sylvestris* (L.) Hoffm.** – Life form: Herb. Habitat: Forest margins and clearings in montane forest, 1800–3970 m. Voucher: Hedberg 1643 (EA).

***Berula erecta* (Huds.) Coville** – Life form: Herb. Habitat: Stream banks and swampy sites, 1000–1900 m. Voucher: Fries & Fries 216 (EA).

***Centella asiatica* (L.) Urb.** – Life form: Herb. Habitat: Wet grassland and open grounds in moist forest, 0–3540 m. Voucher: Kayombo et al. 5290 (EA).

***Haplosciadium abyssinicum* Hochst.** – Life form: Herb. Habitat: Moist montane forest and moorland, 2150–4600 m. Voucher: SK 0084 (EA, HIB).

***Heracleum abyssinicum* (Boiss.) C.Norman** – Life form: Herb. Habitat: Upland moist grassland, 1680–3970 m. Voucher: Townsend 2421 (EA).

***Heracleum elgonense* (H.Wolff) Bullock** – Life form: Herb. Habitat: Damp sites in moorland, 1080–4200 m. Voucher: SK 0115 (EA, HIB).

***Heracleum taylorii* C.Norman** – Life form: Herb. Habitat: Moorland, 3000–3800 m. Voucher: Taylor 1454 (EA).

***Oenanthe palustris* (Chiov.) C.Norman** – Life form: Herbaceous climber. Habitat: Moist forest edges, 1130–3260 m. Voucher: Carmichael 1293 (EA).

***Oenanthe procumbens* (H.Wolff) Norman** – Life form: Herbaceous climber. Habitat: Moist montane forest and shades in bamboo thickets, 1360–3200 m. Voucher: Townsend 2201 (EA).

***Oreoschimperella aberdarensis* (Norman) Rauschert** – Life form: Herb. Habitat: Along streams in upland forest, 2200–2910 m. Voucher: Napier 605 (EA).

***Peucedanum elgonense* H.Wolff** – Life form: Herb. Habitat: Wet grounds in montane forest, 1600–3600 m. Vouchers: Greenway 10397 (EA), SK 0175 (EA, HIB).

***Pimpinella keniensis* C.Norman** – Life form: Herb. Habitat: Wooded grassland, 1550–2500 m. Voucher: SK 0058 (EA, HIB).

**Pimpinella oreophila var. oreophila Hook.f.** – Life form: Herb. Habitat: Moist montane forest, 2300–4100 m. Voucher: Townsend 2428 (EA).

**Pimpinella oreophila var. kilimandscharica (Engl.) C.C.Towns.** – Life form: Herb. Habitat: Glades and grassland in upper montane forest, 3040–4030 m. Voucher: Townsend 2200 (EA).

***Pseudocarum eminii* (Engl.) H.Wolff** – Life form: Herbaceous climber. Habitat: Bamboo thickets, 1750–3350 m. Voucher: Polhill 241 (EA).

***Sanicula elata* Buch.-Ham. ex D.Don.** – Life form: Herb. Habitat: Shaded sites in bamboo thickets, 1240–3220 m. Voucher: Napier 608 (EA).

*Torilis africana Spreng. – Life form: Exotic herb. Habitat: Riverine forest and forest edges, 1360–2720 m. Vouchers: Semsei 2811, Bytebier 264 (EA).

**F45. Apocynaceae**

***Acokanthera schimperi* (A.DC.) Benth. & Hook.f. ex Schweinf.** – Life form: Shrub or small tree. Habitat: Dry forest margins, 250–2200 m. Voucher: Kamau 192 (EA).

* ***Asclepias physocarpa* (E. Mey.) Schltr.** – Life form: Exotic herb or subshrub. Habitat: Roadsides in montane forest, 1500–3000 m. Voucher: SK 0073 (EA, HIB).

**^E^*Brachystelma keniense* Schweinf.** – Life form: Herb. Habitat: Upland dry grassland, 1600–2700 m. Voucher: Hansen 79 (EA).

***Carissa spinarum* L.** – Life form: Shrub. Habitat: Bushland and riverine forest, 0–2250 m. Voucher: Kokwaro 2785 (EA).

***Cynanchum abyssinicum* Decne.** – Life form: Herbaceous climber. Habitat: Bushland and forest margins, 1600–2600 m. Vouchers: Polhill 433, Gilbert 6345 (EA).

***Cynanchum altiscandens* K.Schum.** – Life form: Herbaceous climber. Habitat: Forest margins, 1200–2500 m. Voucher: Mathenge 599 (EA).

**Cynanchum viminale subsp. suberosum (Meve & Liede) Goyder** – Life form: Herbaceous climber. Habitat: Dry rocky grounds in forest, 100–2200 m. Voucher: Rayner 70 (EA).

***Dregea schimperi* (Decne.) Bullock** – Life form: Woody climber. Habitat: Upland forest margins, 1500–2650 m. Voucher: SK 0001 (EA, HIB).

***Gomphocarpus kaessneri* (N.E.Br.) Goyder & Nicholas** – Life form: Herb. Habitat: Seasonal wet pastures, 900–2300 m. Voucher: Faden 71/530 (EA).

***Gomphocarpus semilunatus* A.Rich.** – Life form: Herb. Habitat: Seasonal wet grassland, 1300–2650 m. Voucher: SAJIT 006505 (EA, HIB).

***Gomphocarpus stenophyllus* Oliv.** – Life form: Herb. Habitat: Rocky sites in upland forest, 1200–3050 m. Voucher: Hooper et al 1647 (EA).

***Mondia whitei* (Hook.f.) Skeels** – Life form: Woody climber. Habitat: Upland moist forest, 1600– 2000 m. Voucher: Bell 1 (EA).

***Orbea sprengeri* (Schweinf.) Bruyns** – Life form: Herb. Habitat: Upland open woodland, 1400–2100 m. Voucher: Perkins s.n. (EA).

***Pachycarpus concolor* E.Mey.** – Life form: Herb. Habitat: Upland swampy grassland, 1450–2100 m. Voucher: Faden et al. 74/574 (EA).

**Cynanchum ethiopicum subsp. angolense (N.E.Br.) Liede & Khanum** – Life form: Herbaceous climber. Habitat: Moist forest, 1400–2200 m. Voucher: Napier 2475 (EA).

***Cynanchum gonoloboides* Schltr.** – Life form: Herbaceous climber. Habitat: Montane forest, 2400–3600 m. Voucher: SK 0196 (EA, HIB).

***Pergularia daemia* (Forssk) Chiov.** – Life form: Herbaceous climber. Habitat: Dry bushland, 0–2000 m. Voucher: SK 0207 (EA, HIB).

***Periploca linearifolia* Quart.-Dill. & A.Rich.** – Life form: Herbaceous climber. Habitat: Upland forest edges and riparian scrub forest, 1900–2900 m. Voucher: SK 0198 (EA, HIB).

***Secamone alpini* Schult.** – Life form: Woody climber. Habitat: Montane forest, 1300–2150 m. Voucher: Luke et al. 8923 (EA).

***Secamone punctulata* Decne.** – Life form: Woody climber. Habitat: Bush thickets and riverine forest, 0–2400 m. Voucher: Someren 15954 (EA).

***Tabernaemontana stapfiana* Britten** – Life form: Tree. Habitat: Moist forest, 1400–2370 m. Voucher: Beentje 2721 (EA).

***Tacazzea conferta* N.E.Br.** – Life form: Woody climber. Habitat: Moist bushlands, 1500–3000 m. Voucher: Gillett 16649 (EA).

***Tylophora anomala* N.E.Br.** – Life form: Herbaceous climber. Habitat: Margins of montane forest, 2000–2500 m. Voucher: SK 0251 (EA, HIB).

***Tylophora heterophylla* A.Rich.** – Life form: Woody climber. Habitat: Moist montane forest, 2200–3000 m. Voucher: Mbale et al. 858 (EA).

***Tylophora lugardae* Bullock** – Life form: Herbaceous climber. Habitat: Margins of montane forest, 2000–2500 m. Voucher: Kirrika 158 (EA).

**F46. Aquifoliaceae**

***Ilex mitis* (L.) Radlk.** – Life form: Tree. Habitat: Upland moist forest, 900–3150 m. Voucher: Beentje 2721 (EA).

**F47. Araliaceae**

***Cussonia holstii* Harms ex Engl.** – Life form: Tree. Habitat: Dry evergreen forest, 1110–2550 m. Voucher: SK 0270 (EA, HIB).

***Hydrocotyle mannii* Hook.f.** – Life form: Herb. Habitat: Roadsides and forest margins, 600–3370 m. Voucher: Luke 3887 (EA).

***Hydrocotyle ranunculoides* L.f.** – Life form: Herb. Habitat: Along streams and swamps, 700–2400 m. Voucher: Kibui et al. 2867 (EA).

***Hydrocotyle sibthorpioides* Lam.** – Life form: Herb. Habitat: Bogs, damp grassland and swamps in montane forest, 1134–3900 m. Voucher: SAJIT 006468 (EA, HIB).

***Polyscias fulva* (Hiern) Harms** – Life form: Tree. Habitat: Upland moist forest and riverine forest, 1180–2300 m. Voucher: SK 0164 (EA, HIB).

***Polyscias kikuyuensis* Summerh.** – Life form: Tree. Habitat: Upland moist forest, 1750–2620 m. Voucher: Kirika 28 (EA).

***Schefflera myriantha* (Baker) Drake** – Life form: Woody climber. Habitat: Moist bamboo thickets, 1540–2770 m. Voucher: Verdcourt et al. 2974 (EA).

***Schefflera volkensii* (Harms) Harms** – Life form: Tree. Habitat: Upland moist forest, 1550–3230 m. Voucher: Muchiri 588 (EA).

**F48. Aristolochiaceae**

***Aristolochia littoralis* Parodi** – Life form: Woody climber. Habitat: Riverine forest, 1080–1800 m. Voucher: Gachathi 76/135 (EA).

**F49. Asteraceae**

*Acanthospermum glabratum (DC.) Wild – Life form: Exotic herb. Habitat: Roadsides in moist forest, 150–1850 m. Voucher: Mbevi 177 (EA).

***Acmella caulirhiza* Delile** – Life form: Herb. Habitat: Along streams and forest margins, 600–2600 m. Voucher: SK 0160 (EA, HIB).

***Anthemis tigrensis* J. Gay ex A.Rich.** – Life form: Herb. Habitat: Montane grassland and moorland, 1950–4300 m. Vouchers: SAJIT 006473, SK 0079 (EA, HIB).

***Artemisia afra* Jacq. ex Willd** – Life form: Woody herb. Habitat: Montane forest and moorland, 1500–4050 m. Voucher: Molony s.n. (EA).

***Baccharoides lasiopus* (O.Hoffm.) H.Rob.** – Life form: Shrub. Habitat: Roadsides and forest margins, 1100–2800 m. Voucher: Mungai 123(EA).

***Berkheya spekeana* Oliv.** – Life form: Woody herb. Habitat: Rocky slopes in upland forest, 1800–3140 m. Voucher: Napier 1272 (EA).

***Bidens cinerea* Sherff** – Life form: Herb. Habitat: Dry bushland, 900–1950 m. Voucher: Verdcourt 3198 (EA).

***Bidens pilosa* L.** – Life form: Herb. Habitat: Forest margins and grassland, 750–2500 m. Voucher: Kerfoot 643 (EA).

***Bidens rueppellii* (Sch.Bip. ex Sch.Bip.) Sherff** – Life form: Herb or subshrub. Habitat: Moist forest margins, 2250–2800 m. Voucher: Pierce 2568 (EA).

***Bidens whytei* Sherff** – Life form: Herb. Habitat: Riverine vegetation and moist forest margin, 750–2500 m. Voucher: Verdcourt 1479 (EA).

***Blumea axillaris* (Lam.) DC.** – Life form: Herb. Habitat: Swampy sites in grassland, 0–2400 m. Voucher: SK 0067 (EA, HIB).

***Blumea elatior* (R.E.Fr.) Lisowski** – Life form: Herb. Habitat: Swampy sites in grassland, 0–2400 m. Voucher: SK 0264 (EA, HIB).

***Bothriocline amplifolia* (O.Hoffm. & Muschl.) M.G.Gilbert** – Life form: Woody herb or shrub. Habitat: Montane forest, 2100–2850 m. Voucher: Mungai 89/171 (EA).

***Bothriocline fusca* (S.Moore) M.G.Gilbert** – Life form: Shrub. Habitat: Montane forest, 1800–3600 m. Voucher: SAJIT 006475 (EA, HIB).

***Bothriocline glabrescens* C.Jeffrey** – Life form: Shrub. Habitat: Montane forest, 1950–3050 m. Voucher: Schmitt and Mathenge 1061 (EA).

***Bothriocline longipes* (Oliv. & Hiern) N.E.Br.** – Life form: Woody herb or shrub. Habitat: Upland grassland and forest margins, 850–2700 m. Voucher: Waiganjo 36 (EA).

***Carduus afromontanus* R.E.Fr.** – Life form: Herb. Habitat: Moist bamboo thicket, 2350–3430 m. Voucher: Agnew et al. 5623 (EA)

***Carduus keniensis* R.E.Fr.** – Life form: Herb. Habitat: Moist sites in moorland, 2950–4570 m. Voucher: Leakey 1226 (EA).

**^E^*Carduus millefolius* R.E.Fr.** – Life form: Herb. Habitat: Moorland and open grounds in montane forest, 2750–3200 m. Voucher: Kuchar 9579 (EA).

**Carduus nyassanus subsp. nyassanus (S.Moore) R.E.Fr.** – Life form: Herb. Habitat: Upland moist grassland, 1650–3150 m. Voucher: Kerfoot 650 (EA).

**Carduus nyassanus subsp. kikuyorum (R.E.Fr.) C.Jeffrey** – Life form: Herb. Habitat: Moorland and upper margins of bamboo thickets, 1500–3450 m. Voucher: Kuchar 12343 (EA).

**Carduus schimperi subsp. nanus (R.E.Fr.) C.Jeffrey** – Life form: Herb. Habitat: Grassland and moorland, 2550–4050 m. Vouchers: Coe 774, Meinertyhegen AH (EA).

***Carduus silvarum* R.E.Fr.** – Life form: Herb. Habitat: Upland dry forest, 1700–2300 m. Voucher: Verdcourt 3268 (EA).

***Centrapalus pauciflorus* (Willd.) H.Rob.** – Life form: Herb. Habitat: Dry forest margins, 800–2200 m. Voucher: Mungai 119 (EA).

***Cineraria deltoidea* Sond.** – Life form: Herb. Habitat: Montane forest and forest edges, 1890–4050 m. Vouchers: SAJIT 006474, SK 0080, 0179 & 0267 (EA, HIB).

***Cirsium vulgare* (Savi) Ten.** – Life form: Herb. Habitat: Roadsides in upland grassland, 1790–2400 m. Voucher: Kuchar 9566 (EA).

***Conyza clarenceana* Oliv. & Hiern** – Life form: Shrub. Habitat: Damp sites in moorland, 2300–3300 m. Voucher: Kuchar 10306 (EA).

***Conyza hochstetteri* Sch.Bip. ex A.Rich.** Life form: Herb. Habitat: Wooded grassland, 1050–3600 m. Voucher: Townsend (EA).

***Conyza hypoleuca* A.Rich.** – Life form: Woody herb or shrub. Habitat: Upland dry forest and forest margins, 1800–3000 m. Voucher: Kirika et al. 915 (EA).

***Conyza newii* Oliv. & Hiern** – Life form: Woody herb or shrub. Habitat: Upland forest margins and bushlands, 1500–3050 m. Voucher: Luke 15354 (EA).

***Conyza pallidiflora* R.E.Fr.** – Life form: Woody herb. Habitat: Upland moist forest and river banks, 1800–3000 m. Voucher: SK 0181 (EA, HIB).

***Conyza ruwenzoriensis* (S.Moore) R.E.Fr.** – Life form: Herb or shrub. Habitat: Afromontane grassland, 2500–3700 m. Voucher: Napier 599 (EA).

***Conyza schimperi* Sch.Bip. ex A.Rich.** – Life form: Woody herb or shrub. Habitat: Upland grassland, 1650–3000 m. Voucher: Kirika et al. 1051 (EA).

***Conyza subscaposa* O.Hoffm.** – Life form: Herb. Habitat: Afromontane grassland, 1200–4400 m. Voucher: Kerfoot 1519 (EA).

***Conyza tigrensis* Oliv. & Hiern** – Life form: Herb. Habitat: Roadsides in montane forest, 1350–3000 m. Voucher: Verdcourt & Lucas 320 (EA).

***Conyza vernonioides* (Sch.Bip. ex A.Rich.) Wild** – Life form: Shrub or small tree. Habitat: Montane forest margins, 2300–4000 m. Voucher: Battiscombe 946 (EA).

***Cotula abyssinica* Sch.Bip. ex A.Rich.** – Life form: Herb. Habitat: Montane grassland and forest margins, 2100–3650 m. Voucher: Agnew and Timberlake 11121 (EA).

***Crassocephalum crepidioides* (Benth.) S.Moore** – Life form: Herb. Habitat: Roadsides in moist forest, 0–2500 m. Voucher: Mutanga 33 (EA).

***Crassocephalum montuosum* (S.Moore) Milne-Redh.** – Life form: Herb or subshrub. Habitat: Upland moist forest, 900–3260 m. Voucher: Kerfoot 671 (EA).

***Crassocephalum vitellinum* (Benth.) S.Moore** – Life form: Herb. Habitat: Bushland and forest margins, 1500–2800 m. Voucher: Ericksson 607 (EA).

***Crepis carbonaria* Sch.Bip.** – Life form: Herb. Habitat: Montane grassland, 2960–3850 m. Voucher: Kerfoot 1393 (EA).

**Crepis newii subsp. oliveriana (Kuntze) C.Jeffrey & Beentje** – Life form: Herb. Habitat: Montane grassland, 1650–3750 m. Vouchers: Hedberg 1639, Coe & Kirika 422 (EA).

***Crepis rueppellii* Sch.Bip.** – Life form: Herb. Habitat: Upland grassland, 1300–3200 m. Vouchers: SAJIT 006470, SK 0173, SK 0056 (EA, HIB).

***Dendrosenecio battiscombei* (R.E.Fr. & T.C.E.Fr.) E.B.Knox** – Life form: Shrub. Habitat: Wet sites in afro-alpine zones, 2950–4000 m. Voucher: Dale 6981 (EA).

**^E^*Dendrosenecio brassiciformis* (R.E.Fr. & T.C.E.Fr.) Mabb.** – Life form: Shrub. Habitat: Afro-alpine zone, 2950–3950 m. Voucher: Mabberley 379 (EA).

**^E^*Dendrosenecio keniensis* (Baker f.) Mabb.** – Life form: Shrub. Habitat: Afro-alpine zone, 3300–4275 m. Voucher: Faden 70/697 (EA).

**^E^*Dendrosenecio keniodendron* (R.E.Fr. & T.C.E.Fr.) B.Nord.** – Life form: Shrub. Habitat: Afro-alpine zone, 3650–4350 m. Voucher: Kirika et al. 51 (EA).

**Dichrocephala chrysanthemifolia var. alpina (R.E.Fr.) Beentje** – Life form: Herb. Habitat: Damp sites in moorland, 2200–3600 m. Vouchers: Bally 1227, Napier 625 (EA).

***Dichrocephala integrifolia* (L.f.) Kuntze** – Life form: Herb. Habitat: Moist forest margins and wooded grassland, 1100–3670 m. Voucher: Mwangangi 985 (EA).

***Echinops aberdaricus* R.E.Fr.** – Life form: Herb. Habitat: Montane grassland and moorland, 2400–3450 m. Voucher: Kuchar 12706 (EA).

***Echinops angustilobus* S.Moore** – Life form: Herb. Habitat: Bushed grassland, 2000–2850 m. Voucher: Kerfoot 2945 (EA).

***Echinops hoehnelii* Schweinf.** – Life form: Herb. Habitat: Open sites in montane forest, 2200–3500 m. Voucher: SK 0149 (EA, HIB).

*Erigeron bonariensis L. – Life form: Exotic herb. Habitat: Roadsides in grassland, 300–2850 m. Voucher: Perdue and Kibuwa 8414 (EA).

***Ethulia scheffleri* S.Moore** – Life form: Herb or subshrub. Habitat: Marshy grassland and river banks, 1500–2500 m. Voucher: Bally 13211 (EA).

***Euryops brownei* S.Moore** – Life form: Woody herb or shrub. Habitat: Upper parts of montane forest and heath zone, 2300–4000 m. Voucher: SK 0088 (EA, HIB).

***Euryops chrysanthemoides* (DC.) B.Nord.** – Life form: Shrub. Habitat: Montane grassland and bushland, 1500–2100 m. Voucher: SK 0190 (EA, HIB).

***Euryops jacksonii* S.Moore** – Life form: Woody herb or shrub. Habitat: Montane grassland and bushland, 2000–3300 m. Voucher: Durie 1533 (EA).

***Gerbera piloselloides* (L.) Cass.** – Life form: Herb. Habitat: Wet woodland, moist forest and moorland, 900–3700 m. Voucher: Napier 646(EA).

**Gnaphalium unionis var. tweediae (Hilliard) Beentje** – Life form: Herb. Habitat: Montane grassland, 1600–3200 m. Voucher: Townsend 2313 (EA).

***Guizotia jacksonii* (S.Moore) J. Baagøe** – Life form: Herb. Habitat: Montane forest glades and moorland, 2350–3900 m. Voucher: SK 0052 (EA, HIB).

***Gutenbergia rueppellii* Sch.Bip.** – Life form: Herb. Habitat: Open woodland and grassland, 900–2300 m. Voucher: Rauh 500 (EA).

***Gymnanthemum urticifolium* (A.Rich.) H.Rob.** – Life form: Shrubland. Habitat: Upland forest margins, 2160–3000 m. Voucher: Greenway 9734 (EA).

***Gynura scandens* O.Hoffm.** – Life form: Herbaceous climber. Habitat: Moist forest margins and glades, 0–2200 m. Voucher: Napier 2452 (EA).

***Haplocarpha rueppellii* (Sch.Bip.) K.Lewin** – Life form: Herb. Habitat: Moist montane grassland and moorland, 2550–4650 m. Voucher: SK 0083 (EA, HIB).

***Helichrysum argyranthum* O.Hoffm.** – Life form: Woody herb or shrub. Habitat: Montane grassland and moorland, 2100–3900 m. Voucher: SAJIT 006477 (EA, HIB).

**^E^*Helichrysum brownei* S.Moore** – Life form: Herb or subshrub. Habitat: Rocky slopes and ridges, 3300–4500 m. Voucher: SK 0086 (EA).

***Helichrysum chionoides* Philipson** – Life form: Shrub. Habitat: Moorland and bamboo zone, 2800–3850 m. Voucher: Coe & Kirika 327 (EA).

**Helichrysum citrispinum var. hoehnelii (Schweinf.) Schweinf. & Hedberg** – Life form: Shrub. Habitat: Rocky sites in afro-alpine zone, 3200–5100 m. Vouchers: Young 5, Townsend 2402 (EA).

***Helichrysum ellipticifolium* Moeser** – Life form: Herb or subshrub. Habitat: Damp sites in bamboo zone, 2500–4800 m. Voucher: Kuchar 12500 (EA).

***Helichrysum foetidum* (L.) Cass.** – Life form: Herb. Habitat: Upland forest margins and clearings, 1350–3000 m. Voucher: Polhill et al. 319 (EA).

***Helichrysum formosissimum* Sch.Bip.** – Life form: Woody herb or shrub. Habitat: Swamps and bogs in moorland, 2300–4200 m. Voucher: Kuchar 12744 (EA).

**Helichrysum formosissimum var. guilelmii (Engl.) Mesfin** – Life form: Woody herb or shrub. Habitat: Wet sites in moorland, 1800–4200 m. Voucher: Kuchar 2694 (EA).

**Helichrysum forskahlii var. compactum (Vatke) Mesfin.** – Life form: Woody herb or shrub. Habitat: Moorland, 1200–5000 m. Vouchers: Perdue and Kibuwa 8244, Kirika et al. 1063 (EA).

***Helichrysum globosum* Sch.Bip.** – Life form: Herb. Habitat: Upland grassland and forest margins, 900–3100 m. Voucher: Coe 783 (EA).

**^E^*Helichrysum gloria-dei* Chiov.** – Life form: Shrub. Habitat: Rocky sites in afro-alpine zone, 3650–4000 m. Voucher: Taylor 1495 (EA).

***Helichrysum kilimanjari* Oliv.** – Life form: Herb. Habitat: Montane grassland and heathland, 1650–3900 m. Voucher: Agnew 7205 (EA).

***Helichrysum maranguense* O.Hoffm.** – Life form: Shrub. Habitat: Upland forest margins and bamboo zone, 1950–2700 m. Voucher: Fries and Fries 2324 (EA).

***Helichrysum newii* Oliv. & Hiern** – Life form: Shrub. Habitat: Montane grassland, 2700–4600 m. Voucher: Knox 2851 (EA).

***Helichrysum nudifolium* (L.) Less.** – Life form: Woody herb or shrub. Habitat: Upland grassland, 600–2750 m. Voucher: Faden & Evans 74/581 (EA).

***Helichrysum odoratissimum* (L.) Sweet** – Life form: Woody herb or shrub. Habitat: Montane grassland and bushland, 1700–3700 m. Voucher: Kuchar 12426 (EA).

***Helichrysum schimperi* (Sch.Bip. ex A.Rich.) Moeser** – Life form: Woody herb or shrub. Habitat: Upland grassland, 1350–3300 m. Voucher: Kokwaro and Mathenge 3271 (EA).

***Helichrysum setosum* Harv.** – Life form: Herb. Habitat: Clearings in forest and grassland, 1250–3000 m. Voucher: Natrass 1375 (EA).

***Helichrysum stenopterum* DC.** – Life form: Herb. Habitat: Montane grassland and forest margins, 600–2800 m. Voucher: Kirika 161 (EA).

***Hilliardiella aristata* (DC.) H.Rob.** – Life form: Herb. Habitat: Upland grassland and woodland, 1600–2400 m. Voucher: Faden 67/722 (EA).

***Hypochaeris glabra* L.** – Life form: Herb. Habitat: Roadsides in grassland, 1850–3000 m. Voucher: Kuchar 12290 (EA).

**Kleinia abyssinica var. hildebrandtii (Vatke) C.Jeffrey** – Life form: Herb. Habitat: Dry grassland and woodland, 20–2700 m. Voucher: Bogdan 499 (EA).

***Lactuca glandulifera* Hook.f.** – Life form: Herb. Habitat: Moist forest and grassland, 1200–3600 m. Voucher: SK 0222 (EA, HIB).

***Lactuca inermis* Forssk.** – Life form: Herb. Habitat: Roadsides and disturbed sites in grassland, 500–3300 m. Voucher: Malombe & Kirika 22 (EA).

***Lactuca paradoxa* Sch.Bip. ex A.Rich.** – Life form: Herb. Habitat: Upland forest margins and thickets, 1950–2650 m. Voucher: Kokwaro and Mathenge 3348 (EA).

***Laphangium luteoalbum* (L.) Tzvelev** – Life form: Herb. Habitat: Montane grassland, 300–3850 m. Voucher: Muasya 2148 (EA).

***Linzia glabra* Steetz** – Life form: Woody herb. Habitat: Roadsides in bushland, 1300–2160 m. Voucher: Gilbert and Thulin 1750 (EA).

***Linzia ituriensis* (Muschl.) H.Rob.** – Life form: Woody herb. Habitat: Moist forest, 1200–2500 m. Voucher: Verdcourt 3267 (EA).

***Melanthera scandens* (Schumach. & Thonn.) Roberty** – Life form: Herb. Habitat: Riverine forest and swampy sites, 250–2200 m. Voucher: Perdue and Kibuwa 8201 (EA).

***Micractis bojeri* DC.** – Life form: Herb. Habitat: Moist forest and swamp grassland, 1350–2750 m. Voucher: Verdcourt et al. 3030 (EA).

***Microglossa densiflora* Hook.f.** – Life form: Shrub. Habitat: Montane forest margins, 1200–2650 m. Voucher: Mathenge 192 (EA).

***Microglossa pyrifolia* (Lam.) Kuntze** – Life form: Shrub. Habitat: Grassland and bushland, 50–2650 m. Voucher: Kokwaro 327 (EA).

***Mikaniopsis bambuseti* (R.E.Fr.) C.Jeffrey** – Life form: Woody climber. Habitat: Bamboo zone, 2100–3150 m. Voucher: Vorontsova et al. 61 (EA).

***Mikaniopsis usambarensis* (Muschl.) Milne-Redh.** – Life form: Woody climber. Habitat: Moist forest margins, 850–2350 m. Voucher: Malombe et al. 27/986 (EA).

***Prenanthes subpeltata* Stebbins** – Life form: Herbaceous climber. Habitat: Bamboo thicket, 2470–2500 m. Voucher: Faden et al. 71/193 (EA).

***Pseudognaphalium oligandrum* (DC.) Hilliard & B.L.Burtt** – Life form: Herb. Habitat: Montane grassland, 1050–2550 m. Voucher: Burney 18 (EA).

***Psiadia punctulata* (DC) Vatke** – Life form: Woody herb or shrub. Habitat: Evergreen bushland and forest edges, 1000–2500 m. Voucher: SK 0010 (EA, HIB).

*Schkuhria pinnata (Lam.) Kuntze ex Thell. – Life form: Exotic herb. Habitat: Upland dry woodland and grassland, 1000–2220 m. Voucher: Mbevi 189 (EA).

***Senecio aequinoctialis* R.E.Fr.** – Life form: Woody herb. Habitat: Moist afro-alpine zone, 3000–4250 m. Voucher: SK 0085 (EA, HIB).

**^E^*Senecio amplificatus* C.Jeffrey** – Life form: Herb. Habitat: Upper regions of giant heath zone, 2900–3500 m. Voucher: Alexander 11635 (EA).

***Senecio crispatipilosus* C.Jeffrey** – Life form: Herb. Habitat: Open sites in bamboo zone, 2050–3050 m. Voucher: Coe 754 (EA).

***Senecio deltoideus* Less.** – Life form: Herb. Habitat: Moist forest margins, 1700–2600 m. Voucher: Kuchar 12245 (EA).

***Senecio hadiensis* Forsk.** – Life form: Herbaceous climber. Habitat: Riverine thickets and forest margins, 500–2600 m. Voucher: Hiepko 2678 (EA).

***Senecio hochstetteri* Sch.Bip. ex A.Rich.** – Life form: Herb. Habitat: Forest margins and wooded grassland, 900–3350 m. Voucher: Rauh 388 (EA).

***Senecio jacksonii* S.Moore** – Life form: Herb. Habitat: Montane swampy grassland, 3250–4150 m. Vouchers: Hedberg 1545, Rauh 2388 (EA).

***Senecio lyratus* Forssk.** – Life form: Woody climber. Habitat: Evergreen dry forest margins, 1500–2760 m. Voucher: Gillet 20736 (EA).

***Senecio maranguensis* O.Hoffm.** – Life form: Woody herb or shrub. Habitat: Montane forest and bamboo thicket, 1800–3250 m. Voucher: Fries and Fries 2699 (EA).

**^E^*Senecio margaritae* C.Jeffrey** – Life form: Shrub. Habitat: Rocky bushland, 1800–1950 m. Voucher: Bally 1013 (EA).

***Senecio mesogrammoides* O.Hoffm.** – Life form: Herb. Habitat: Open grassland, 1200–2500 m. Voucher: SK 0044 (EA, HIB).

***Senecio moorei* R.E.Fr.** – Life form: Woody herb or shrub. Habitat: Montane grassland and heath zone, 1750–3500 m. Voucher: Kuchar 12527 (EA).

***Senecio pseudosubsessilis* C.Jeffrey** – Life form: Herb. Habitat: Upland forest margins and swamps, 1900–3050 m. Voucher: Charles 10580 (EA).

**^E^*Senecio roseiflorus* R.E.Fr.** – Life form: Woody herb or shrub. Habitat: Rocky moorland and giant heath zone, 3100–4200 m. Vouchers: SAJIT 006490, SK 0114 (EA, HIB).

***Senecio schweinfurthii* O.Hoffm.** – Life form: Herb. Habitat: Moorland and clearings in upper montane forest, 2300–4500 m. Voucher: SK 0089 (EA, HIB).

***Senecio subsessilis* Oliv. & Hiern** – Life form: Woody herb or shrub. Habitat: Montane forest margins, 1800–3600 m. Voucher: Kuchar 12550 (EA).

***Senecio syringifolius* O.Hoffm.** – Life form: Herbaceous climber. Habitat: Montane forest margins, 1500–3300 m. Voucher: SK 0103 (EA, HIB).

***Solanecio angulatus* (Vahl) C.Jeffrey** – Life form: Herbaceous climber. Habitat: Riverine vegetation and moist bushland, 1800–2500 m. Voucher: Faden et al. 74/657 (EA).

***Solanecio mannii* (Hook.f.) C.Jeffrey** – Life form: Shrub or small tree. Habitat: Forest glades and margins, 80–2700 m. Voucher: SK 0165 (EA, HIB).

***Solanecio nandensis* (S.Moore) C.Jeffrey** – Life form: Herbaceous climber. Habitat: Forest margins and bushland, 1500–2700 m. Voucher: Kamau 372 (EA).

***Sonchus afromontanus* R.E.Fr.** – Life form: Herb. Habitat: Upland grassland, 2200–3700 m. Voucher: Agnew et al. 142 (EA).

***Sonchus asper* (L.) Hill** – Life form: Herb. Habitat: Upland grassland, 1600–3000 m. Voucher: Someren 63/192 (EA).

***Sonchus bipontini* Asch.** – Life form: Herb. Habitat: Upland grassland, forest glades and margins, 1700–3370 m. Voucher: Hooper and Townsend 1389 (EA).

***Sonchus camporum* (R.E.Fr.) Boulos ex C.Jeffrey** – Life form: Herb. Habitat: Upland grassland, 1800–2300 m. Voucher: Fries & Fries 513b (EA).

***Sonchus luxurians* (R.E.Fr.) C.Jeffrey** – Life form: Herb. Habitat: Roadsides in grassland and montane forest margins, 1600–3800 m. Voucher: Young 1001 (EA).

***Sonchus stenophyllus* R.E.Fr.** – Life form: Herb. Habitat: Upland grassland and forest edges, 2200–3700 m. Voucher: Fries & Fries 123 (EA).

***Sphaeranthus suaveolens* (Forsk.) DC.** – Life form: Herb. Habitat: Stream banks and marshy sites, 350–2500 m. Voucher: Kuchar 8276 (EA).

***Stoebe kilimandscharica* O.Hoffm.** – Life form: Shrub. Habitat: Moist afro-alpine zone, 1950–3900 m. Voucher: Bally 1167 (EA).

*Tagetes minuta L. – Life form: Exotic herb. Habitat: Roadsides and disturbed sites, 850–2750 m. Voucher: Spjut 2791 (EA).

***Taraxacum campylodes* G.E.Haglund** – Life form: Herb. Habitat: Roadsides in montane forest, 2350–2500 m. Voucher: Dyson 638 (EA).

*Tithonia diversifolia (Hemsl.) A.Gray – Life form: Exotic woody herb or subshrub. Habitat: Roadsides and forest margins, 0–1950 m. Voucher: SK 0204 (EA, HIB).

***Tolpis capensis* (L.) Sch.Bip.** – Life form: Herb. Habitat: Open woodland and grassland, 1800–3300 m. Voucher: Kerfoot 1457 (EA).

***Tripteris vaillantii* Decne.** – Life form: Herb or subshrub. Habitat: Upland grassland and bushland, 1200–3000 m. Voucher: Faden et al. 74/646 (EA).

***Vernonia auriculifera* Hiern** – Life form: Woody herb or shrub. Habitat: Montane forest margins and glades, 750–3000 m. Voucher: Kuchar 8342 (EA).

***Vernonia hochstetteri* Sch.Bip. ex Walp.** – Life form: Woody herb or shrub. Habitat: Moist forest and bushlands, 1000–2400 m. Voucher: Polhill 76 (EA).

***Vernonia pteropoda* Oliv. & Hiern** – Life form: Herb or subshrub. Habitat: Wet evergreen forest, 1800–2750 m. Voucher: Verdcourt 2311 (EA).

***Vernonia subscandens* R.E.Fr.** – Life form: Shrub. Habitat: Moist forest and forest margins, 1650–2100 m. Voucher: Verdcourt 3706 (EA).

***Vernonia syringifolia* O.Hoffm.** – Life form: Woody herb or shrub. Habitat: Moist forest and forest margins, 1550–3050 m. Voucher: Kerfoot 465 (EA).

***Vernonia amygdalina* Delile** – Life form: Shrub or small tree. Habitat: Grassland and woodland, 200–2300 m. Voucher: SK 0016 (EA, HIB).

**Vernonia galamensis subsp. afromontana (R.E.Fr.) M.G.Gilbert** – Life form: Shrub. Habitat: Dry forest margins and glades, 800–2200 m. Voucher: SK 0205 (EA, HIB).

**F50. Balsaminaceae**

***Impatiens fischeri* Warb.** – Life form: Herb. Habitat: Moist shaded sites in upland forest, 2000–3100 m. Voucher: SK 0127 (EA, HIB).

***Impatiens hoehnelii* T.C.E.Fr.** – Life form: Herb. Habitat: Moist shaded sites in rainforest, 1475–3350 m. Voucher: SK 0126 (EA, HIB).

**Impatiens meruensis subsp. cruciata (T.C.E.Fr.) Grey-Wilson** – Life form: Herb. Habitat: Moist forest and bamboo thickets, 1100–3630 m. Voucher: Napier 722 (EA).

***Impatiens tinctoria* A.Rich.** – Life form: Herb. Habitat: Waterfalls and stream banks in wet upland forests, 1800–3630 m. Voucher: SK 0178 (EA, HIB).

**F51. Basellaceae**

***Basella alba* L.** – Life form Herbaceous climber. Habitat: Bush thickets and forest edges, 0–2450 m. Voucher: Faden 74/712 (EA).

**F52. Begoniaceae**

***Begonia meyeri-johannis* Engl.** – Life form: Woody climber. Habitat: Moist forest, 1350–2800 m. Vouchers: SK 0183, SK 0187 (EA, HIB).

**F53. Berberidaceae**

***Berberis holstii* Engl.** – Life form: Shrub. Habitat: Upland forest margins and bushland 1500–3450 m. Voucher: Battiscombe 203 (EA).

**F54. Bignoniaceae**

*Jacaranda mimosifolia D.Don – Life form: Exotic tree. Habitat: Cultivated, 1970–1970 m. Voucher: Perdue and Kibuwa 8096 (EA).

**Kigelia africana subsp. moosa (Sprague) Bidgood & Verdc.** – Life form: Tree. Habitat: Moist evergreen forest and swampy forest, 1050–2250 m. Vouchers: Perdue and Kibuwa 8421, Nyakundi 421 (EA).

***Spathodea campanulata* P.Beauv.** – Life form: Tree. Habitat: Montane forest and riverine forest, 1500–2000 m. Voucher: SK 0202 (EA, HIB).

**F55. Boraginaceae**

***Cordia africana* Lam.** – Life form: Tree. Habitat: Forest edges and wooded grassland, 450–2100 m. Voucher: SK 0224 (EA, HIB).

***Cynoglossum aequinoctiale* T.C.E.Fr.** – Life form: Herb. Habitat: Upland grassland, 2100–2870 m. Voucher: Lacey 24A (EA).

**Cynoglossum amplifolium var. amplifolium Hochst. ex A.DC.** – Life form: Herb. Habitat: Grassland and open sites in bamboo thickets, 1800–3200 m. Voucher: Brown 1717 (EA).

**Cynoglossum amplifolium var. subalpinum (T.C.E.Fr.) Verdc.** – Life form: Herb. Habitat: Montane grassland and open sites in bamboo thickets, 2100–3430 m. Voucher: Kerfoot 1418 (EA).

**Cynoglossum coeruleum var. kenyense B. Verdcourt** – Life form: Herb. Habitat: Grassland and forest edges, 1100–3200 m. Vouchers: SAJIT 006460, SK 0075, SK 0174 (EA, HIB).

***Cynoglossum lanceolatum* Forssk.** – Life form: Herb. Habitat: Grassland and bushland, 1100–3220 m. Voucher: Kuchar 12236 (EA).

**Ehretia cymosa var. silvatica (Gürke) Brenan** – Life form: Tree. Habitat: Moist forest and bushland, 960–2250 m. Vouchers: Kenya Forest excursion 88 (EA), SK 0154 (EA, HIB).

***Heliotropium scotteae* Rendle** – Life form: Herb. Habitat: Open sites in upland forests and bushland, 1500–2170 m. Voucher: McDonald 879 (EA).

***Heliotropium zeylanicum* (Burm.f.) Lam.** – Life form: Herb. Habitat: Roadsides in grassland and bushland, 0–2317 m. Voucher: SK 0171 (EA, HIB).

***Lithospermum afromontanum* Weim.** – Life form: Woody climber. Habitat: Montane forest margins, 1560–3950 m. Voucher: SAJIT 006483 (EA, HIB).

***Myosotis abyssinica* Boiss. & Reut.** – Life form: Herb. Habitat: Upland grassland and moorland, 1560–3590 m. Voucher: Mabberley 381 (EA).

***Myosotis vestergrenii* Stroh** – Life form: Herb. Habitat: Wet sites in moorland and bamboo thickets, 2000–4250 m. Voucher: Rauh et al. 527 (EA).

**Trichodesma ambacense subsp. hockii (De Wild.) Brummitt.** – Life form: Herb. Habitat: Burnt grassland and woodland, 780–3000 m. Vouchers: Shayse 8835, Bally 8835 (EA).

***Trichodesma physaloides* (Fenzl) A.DC.** – Life form: Herb. Habitat: Wooded grassland, 700–2400 m. Voucher: SK 0184 (EA).

**F56. Brassicaceae**

***Arabidopsis thaliana* (L.) Heynh.** – Life form: Herb. Habitat: Montane bushland and moorland, 1750–4250 m. Voucher: Coe & Kirika 290 (EA).

***Arabis alpina* L.** – Life form: Herb. Habitat: Damp sites in moorland, 2450–4800 m. Voucher: SAJIT 006461 (EA, HIB).

***Barbarea intermedia* Boreau** – Life form: Herb. Habitat: Streamsides in upper parts of montane forest, 3050–3950 m. Voucher: Muninentyhegen 9313 (EA).

*Brassica napus L. – Life form: Exotic herb. Habitat: Escaped cultivation common along roadsides and disturbed sites, 1750–2300 m. Voucher: Someren s.n (EA).

*Brassica rapa L. – Life form: Exotic herb. Habitat: Escape cultivation common along roadsides, 1500–2600 m. Voucher: Someren 603 (EA).

***Capsella bursa-pastoris* (L.) Medik.** – Life form: Herb. Habitat: Roadsides in montane forest, 1600–2500 m. Voucher: Greenway 10206 (EA).

***Cardamine africana* L.** – Life form: Herb. Habitat: Moist forest, 1000–3400 m. Voucher: Muninentyhegen 9304 (EA).

***Cardamine obliqua* Hochst. ex A.Rich.** – Life form: Herb. Habitat: Moist montane forest up to moorland, 2000–4900 m. Voucher: Hedberg 1596 (EA).

***Cardamine hirsuta* L.** – Life form: Herb. Habitat: Wet open grounds in montane forest, 500–4600 m. Vouchers: SAJIT 006466, SK 0177 (EA, HIB).

***Erucastrum arabicum* Fisch. & C.A.Mey.** – Life form: Herb. Habitat: Roadsides in upland forests, 0–3170 m. Voucher: John Terry 180 (EA).

***Farsetia stenoptera* Hochst.** – Life form: Herb. Habitat: Roadsides in open bushland, 500–3613 m. Voucher: SK 0094 (EA, HIB).

***Farsetia undulicarpa* Jonsell** – Life form: Shrub. Habitat: Bushland and wooded grassland, 1750–2300 m. Voucher: Verdcourt 2159 (EA).

***Lepidium africanum* (Burm.f.) DC.** – Life form: Herb. Habitat: Along the streams and roadsides in upland grassland, 1200–2120 m. Voucher: Harper 2143 (EA).

*Lepidium bonariense L. – Life form: Exotic herb. Habitat: Roadsides in upland grassland, 1460–2650 m. Voucher: Verdcourt 2767 (EA).

***Lepidium didymum* L.** – Life form: Herb. Habitat: Roadsides and forest clearings, 1350–2800 m. Voucher: Kroo 13173 (EA).

***Nasturtium microphyllum* (Boenn. ex Rchb.) Rchb.** – Life form: Herb. Habitat: Upland stream banks, 1500–2000 m. Voucher: Bally 8653 (EA).

***Nasturtium officinale* R.Br.** – Life form: Herb. Habitat: Muddy soils along rivers, 1500–2700 m. Voucher: Bogdan 2140 (EA).

***Oreophyton falcatum* O.E.Schulz** – Life form: Herb. Habitat: Rocky sites in afro-alpine zone, 3820–4900 m. Voucher: Hedberg 1557 (EA).

*Raphanus raphanistrum L. – Life form: Exotic herb. Habitat: Roadsides and other ruderal sites, 15–2750 m. Voucher: Someren 549-556 (EA).

*Raphanus sativus L. – Life form: Exotic herb. Habitat: Roadsides, 15–2650 m. Voucher: Someren 550 (EA).

***Rorippa cryptantha* (A.Rich.) Robyns & Boutique** – Life form: Herb. Habitat: Stream banks and wet sites in forest, 1800–3000 m. Voucher: Bogdan 4757 (EA).

***Rorippa micrantha* (Roth) Jonsell** – Life form: Herb. Habitat: Along streams and muddy sites, 10–2400 m. Voucher: Gillett (EA).

***Rorippa nudiuscula* Thell.** – Life form: Herb. Habitat: Upland moist forest and stream banks, 2200–3000 m. Voucher: Kuchar 8288 (EA).

***Sisymbrium erysimoides* Desf.** – Life form: Herb. Habitat: Clearings in forest and disturbed sites, 2000–2400 m. Voucher: Meinertzhagen 9309 (EA).

***Sisymbrium officinale* (L.) Scop.** – Life form: Herb. Habitat: Roadsides and waste places, 1350–1800 m. Voucher: Frank Msafiri 20 (EA).

***Sisymbrium orientale* L.** – Life form: Herb. Habitat: Roadsides and waste places, 15–2000 m. Voucher: Greenway 14910 (EA).

***Subularia monticola* A.Braun ex Schweinf.** – Life form: Herb. Habitat: Moist montane forest and moorland, 2750–4750 m. Voucher: SAJIT 006467 (EA, HIB).

***Thlaspi alliaceum* L.** – Life form: Herb. Habitat: Upper montane forest and moorland, 3050–3600 m. Voucher: Gillet 16229 (EA).

***Turritis glabra* L.** – Life form: Herb. Habitat: Roadsides in upland moist forest, 920–2700 m. Voucher: Verdcourt 3206 (EA).

**F57. Burseraceae**

**Commiphora africana var. oblongifoliolata (Engl.) J.B.Gillett** – Life form: Shrub or small tree. Habitat: Bushed grassland, 20–1890 m. Voucher: Adamson (EA).

**Commiphora africana var. rubriflora (Engl.) Wild** – Life form: Shrub or small tree. Habitat: Bushed grassland, 640–2070 m. Voucher: Gillett 19442 (EA).

**F58. Campanulaceae**

***Campanula edulis* Forssk.** – Life form: Herb. Habitat: Upland grassland, 1500–3700 m. Voucher: Napier 2107 (EA).

***Canarina eminii* Asch. & Schweinf.** – Life form: Herb. Habitat: Often epiphytic in moist forests, 1600–3200 m. Voucher: SK 0141 (EA, HIB).

***Lobelia aberdarica* R.E.Fr. & T.C.E.Fr.** – Life form: Shrub. Habitat: Swampy sites in montane forest, 1800–3500 m. Voucher: Rauh 520 (EA).

***Lobelia bambuseti* R.E.Fr. & T.C.E.Fr.** – Life form: Shrub. Habitat: Upland forests and bamboo thickets, 2350–4000 m. Voucher: Taylor 1361 (EA).

***Lobelia baumannii* Engl.** – Life form: Shrub. Habitat: Forest floors and margins, 800–2400 m. Voucher: Knox 2575 (EA).

***Lobelia duriprati* T.C.E.Fr.** – Life form: Herb. Habitat: Roadsides and forest margins, 1675–3550 m. Voucher: Agnew and Timberlake 11144 (EA).

**^E^Lobelia gregoriana subsp. sattimae (R.E.Fr. & T.C.E.Fr.) E.B.Knox** – Life form: Shrub. Habitat: Wet places in moorland and stream banks, 3350–3900 m. Vouchers: Hedberg 1608, Kirika and York 1192 (EA).

***Lobelia holstii* Engl.** – Life form: Herb. Habitat: Upland grassland and moorland, 900–3520 m. Voucher: Kerfoot 412 (EA).

***Lobelia lindblomii* Mildbr.** – Life form: Herb. Habitat: Moorland and swampy grounds in montane grassland, 3100–4250 m. Voucher: Knox 2519 (EA).

***Lobelia minutula* Engl.** – Life form: Herb. Habitat: Upland grassland and moorland, 2125–3940 m. Voucher: Polhill 166 (EA).

***Lobelia telekii* Schweinf.** – Life form: Shrub. Habitat: Upland grassland and moorland, 2950–4550 m. Voucher: Kirika et al. 905 (EA).

**Monopsis stellarioides subsp. schimperiana (Urb.) Thulin** – Life form: Herb. Habitat: Upland grassland and moorland, 1100–3600 m. Vouchers: Kuchar 12413, Napier 730 (EA).

**Wahlenbergia capillacea subsp. tenuior (Engl.) Thulin** – Life form: Herb. Habitat: Upland grassland and rocky sites in bamboo zone, 1500–3500 m. Vouchers: Gardner 10120, Leakey 14 (EA).

**Wahlenbergia krebsii subsp. arguta (Hook.f.) Thulin** – Life form: Herb. Habitat: Upland grassland and moorland, 1500–4000 m. Vouchers: Kuchar 9612, 10395 (EA).

***Wahlenbergia pusilla* Hochst. ex A.Rich.** – Life form: Herb. Habitat: Upland grassland and moorland, 2800–4500 m. Voucher: Hedberg 4297 (EA).

***Wahlenbergia scottii* Thulin** – Life form: Herb. Habitat: Upland grassland, 1500–3000 m. Voucher: Bogdan 4451 (EA).

***Wahlenbergia silenoides* Hochst. ex A.Rich.** – Life form: Herb. Habitat: Upland grassland and forest margins, 2200–3350 m. Voucher: Chandler 2317 (EA).

***Wahlenbergia virgata* Engl.** – Life form: Herb. Habitat: Upland grassland, 1100–2700 m. Voucher: H & F 4850 (EA).

**F59. Canellaceae**

***Warburgia ugandensis* Sprague** – Life form: Tree. Habitat: Moist forest, 1100–2230 m. Voucher: Trapnell 2143 (EA).

**F60. Cannabaceae**

***Celtis africana* Burm.f.** – Life form: Tree. Habitat: Upland rainforest and riverine forest, 30–2400 m. Voucher: Kamau 418 (EA).

**F61. Capparaceae**

***Cadaba farinosa* Forssk.** – Life form: Shrub. Habitat: Deciduous bushland and grassland, 0–1900 m. Voucher: SK 0007 (EA, HIB).

**Capparis fascicularis var. elaeagnoides (Gilg) DeWolf** – Life form: Woody climber. Habitat: Deciduous bushland and grassland, 900–2100 m. Vouchers: Battiscombe 1091 (EA), SK 0004 (EA, HIB).

***Capparis tomentosa* Lam.** – Life form: Shrub or small tree. Habitat: Bushland and grassland, 0–2500 m. Voucher: Someren 1769 (EA).

***Capparis viminea* Oliv.** – Life form: Shrub. Habitat: Moist forest, 0–2030 m. Voucher: Battiscombe 562 (EA).

***Maerua triphylla* A.Rich.** – Life form: Shrub. Habitat: Grassland and bushland, 0–2300 m. Voucher: Agnew et al. 8724 (EA).

***Ritchiea albersii* Gilg** – Life form: Tree. Habitat: Upland rainforest, 1100–2400 m. Voucher: SK 0155 (EA, HIB).

***Thylacium africanum* Lour.** – Life form: Shrub or small tree. Habitat: Wooded grassland and deciduous bushland, 1–2400 m. Voucher: SK 0026 (EA, HIB).

**F62. Caprifoliaceae**

***Dipsacus pinnatifidus* Steud. ex A.Rich.** – Life form: Herb. Habitat: Clearings in upland forests and bamboo thickets, 2000–3950 m. Voucher: Napier 664 (EA, HIB).

***Scabiosa columbaria* L.** – Life form: Herb. Habitat: Upland grassland and moorland, 2100–4100 m. Voucher: SK 0092 (EA, HIB).

***Valeriana capensis* Thunb.** – Life form: Herb. Habitat: Upland moist forests and moorland, 1500–3400 m. Voucher: Coe 757 (EA).

***Valeriana kilimandscharica* Engl.** – Life form: Woody herb. Habitat: Wet sites in moorland and tussocky grassland, 2800–4500 m. Voucher: Dale 294 (EA).

***Valerianella microcarpa* Loisel.** – Life form: Herb. Habitat: Moorlands and upper parts of bamboo zone, 2800–3500 m. Voucher: Kokwaro et al. 2423 (EA).

**F63. Caryophyllaceae**

***Cerastium afromontanum* T.C.E.Fr.** – Life form: Herb. Habitat: Moorland, 2100–3940 m. Voucher: SK 0061 (EA).

***Cerastium lanceolatum* (Poir.) Volponi** – Life form: Herb. Habitat: Forest margins and glades, 1050–3600 m. Voucher: Drummond and Hemsley 4283 (EA).

**Cerastium octandrum var. adnivale (Chiov.) Möschl** – Life form: Herb. Habitat: Montane bushland edges and open grassland, 1920–4200 m. Vouchers: Gillett 18988, Kuchar 12460 (EA).

***Corrigiola litoralis* L.** – Life form: Herb. Habitat: Roadsides in montane forest, 1200–2190 m. Voucher: Mathenge 208 (EA).

***Drymaria cordata* (L.) Willd. ex Schult.** – Life form: Herb. Habitat: Roadsides in forest and bushland, 870–2700 m. Voucher: SK 0136 (EA, HIB).

***Sagina abyssinica* Hochst. ex A.Rich.** – Life form: Herb. Habitat: Damp sites in moorland, 2150–4250 m. Voucher: Coe 784 (EA).

***Sagina afroalpina* Hedberg** – Life form: Herb. Habitat: Bogs and swamps in montane forest, 2980–4600 m. Voucher: Hedberg 1542 (EA).

***Silene burchellii* Otth ex DC.** – Life form: Herb. Habitat: Rocky grounds in moorland, 1500–4050 m. Voucher: Kuchar 10349 (EA).

***Silene macrosolen* Steud. ex A.Rich.** – Life form: Herb. Habitat: Upland rocky grassland, 1800–3300 m. Voucher: Bally 906 (EA).

***Stellaria sennii* Chiov.** – Life form: Herb. Habitat: Roadsides in upland wet forests, 1650–3440 m. Voucher: Young 1002 (EA).

***Uebelinia crassifolia* T.C.E.Fr.** – Life form: Herb. Habitat: Grassy glades in bamboo thickets and moorland, 2500–4000 m. Voucher: Miss Dent 1306 (EA).

**F64. Celastraceae**

***Cassine buchananii* Loes.** – Life form: Tree. Habitat: Dry evergreen forest and wooded grassland, 1000–2330 m. Voucher: Verdcourt 3049 (EA).

***Gymnosporia buchananii* Loes.** – Life form: Shrub. Habitat: Dry evergreen forest, 60–2640 m. Voucher: Bogdan 467 (EA).

***Gymnosporia heterophylla* (Eckl. & Zeyh.) Loes.** – Life form: Shrub. Habitat: Moist forest and riverine forest, 0–2670 m. Voucher: Ward 3049 (EA).

***Gymnosporia putterlickioides* Loes.** – Life form: Shrub. Habitat: Dry woodland, 850–1800 m. Vouchers: H & J 6603 (EA) SK 0100 (EA, HIB).

***Hippocratea goetzei* Loes.** – Life form: Woody climber. Habitat: Evergreen forest, 0–3000 m. Voucher: SK 0247 (EA, HIB).

***Maytenus obscura* (A.Rich.) Cufod.** – Life form: Shrub or small tree. Habitat: Moist forest, 2100–2550 m. Voucher: Bogdan 468 (EA).

***Maytenus undata* (Thunb.) Blakelock** – Life form: Tree. Habitat: Moist forest, 0–3150 m. Voucher: Kuchar 10284 (EA).

***Cassine aethiopicum* (Thunb.) Loes.** – Life form: Tree. Habitat: Moist forest, 0–2550 m. Voucher: Hansen 804 (EA).

***Pristimera goetzei* (Loes.) R. H. Archer** – Life form: Herb or subshrub. Habitat: Moist forest, 90–3000 m. Voucher: Beentje and Mungai 2898 (EA).

**F65. Cleomaceae**

***Cleome gynandra* L.** – Life form: Herb. Habitat: Roadsides and disturbed sites, 0–2400 m. Voucher: Ward 10861 (EA).

**F66. Clusiaceae**

***Garcinia volkensii* Engl.** – Life form: Tree. Habitat: Moist or dry evergreen forest, 30–2400 m. Voucher: Kuchar et al. 5455 (EA).

**F67. Connaraceae**

***Agelaea pentagyna* (Lam.) Baill.** – Life form: Woody climber. Habitat: Upland wet forest, 1200–2100 m. Voucher: Ndonge 37 (EA).

***Rourea thomsonii* (Baker.) Jongkind** – Life form: Shrub or small tree. Habitat: Upland wet forest, 0–2500 m. Voucher: Kuchar 5460 (EA).

**F68. Convolvulaceae**

***Convolvulus farinosus* L -.** Life form: Herbaceous climber. Habitat: Upland grassland, 450–2600 m. Voucher: Faden 67/270 (EA).

***Convolvulus kilimandschari* Engl.** – Life form: Herbaceous climber. Habitat: Moist forest margins and bamboo thicket, 1800–3750 m. Voucher: Svarreush 18 (EA).

***Convolvulus siculus* L.** – Life form: Herb. Habitat: Grassland, 1800–2300 m. Voucher: Faden 67/270 (EA).

***Cuscuta australis* R.Br.** – Life form: Herbaceous climber. Habitat: Parasitic in swampy vegetation, 1750–2170 m. Voucher: Gillett 16568 (EA).

***Cuscuta kilimanjari* Oliv.** – Life form: Herbaceous climber. Habitat: Parasitic in upland moist forest, 500–2770 m. Voucher: Faden 74/709 (EA).

**Cuscuta planiflora var. madagascarensis (Yunck.) Verdc.** – Life form: Herbaceous climber. Habitat: Parasitic in upland grassland, 1500–3000 m. Vouchers: Bogdan 839, Gillett 16568 (EA).

***Dichondra repens* J.R.Forst. & G.Forst.** – Life form: Herb. Habitat: Upland grassland, 1650–2520 m. Voucher: Faden 67418 (EA).

***Ipomoea alba* L.** – Life form: Herbaceous climber. Habitat: Moist forest, 420–3393 m. Voucher: SAJIT 006487 (EA, HIB).

***Ipomoea purpurea* (L.) Roth** – Life form: Herbaceous climber. Habitat: Escaped cultivation common on roadsides and other waste places, 900–2040 m. Voucher: Greenway 10950 (EA).

**Ipomoea tenuirostris subsp. tenuirostris Steud. ex Choisy** – Life form: Herb. Habitat: Upland bushland, 1350–2250 m. Vouchers: Faden 68/283 (EA), SK 0048 (EA, HIB).

***Ipomoea wightii* (Wall.) Choisy** – Life form: Herb. Habitat: Upland grassland and montane forest margins, 1040–2400 m. Voucher: Beentje 3195 (EA).

**F69. Cornaceae**

***Cornus volkensii* Harms** – Life form: Tree. Habitat: Moist forest and riparian forest, 1200–3000 m. Vouchers: Dale 402 (EA), SK 0131 (EA, HIB).

**F70. Crassulaceae**

**Crassula alata subsp. pharnaceoides (Fisch. & C.A.Mey.) Wickens & Bywater** – Life form: Herb. Habitat: Moist rocky sites and stream banks, 1900–2100 m. Vouchers: Hedberg 6278, Gilbert 4835 (EA).

***Crassula alsinoides* (Hook.f.) Engl.** – Life form: Herb. Habitat: Along streams and swamps, 1300–3500 m. Voucher: Agnew 7164 (EA).

***Crassula granvikii* Mildbr.** – Life form: Herb. Habitat: Along streams in alpine zone and damp open soils, 1200–4250 m. Voucher: Gillet 18059 (EA).

***Crassula rhodesica* (Merxm.) Wickens & M.Bywater** – Life form: Herb. Habitat: Forest shades especially on rocky grounds, 1200–2100 m. Voucher: Verdcourt 670 (EA).

***Crassula schimperi* Fisch. & C.A.Mey.** – Life form: Herb. Habitat: Moist rocky places in grassland, 1050–3820 m. Voucher: Bogdan 4635 (EA).

***Kalanchoe densiflora* Rolfe** – Life form: Herb. Habitat: Upland forest margins and grassland, 1000–3000 m. Voucher: SK 0072 (EA, HIB).

***Sedum crassularia* (Schwienf.) R.-Hamet** – Life form: Herb. Habitat: Rocky sites in moorland, 3300–4300 m. Voucher: Hedberg 1552 (EA).

***Sedum meyeri-johannis* Engl.** – Life form: Herb. Habitat: Epiphytic in moist forest and rocky heathland, 2100–3150 m. Voucher: SK 0265 (EA, HIB).

***Sedum ruwenzoriense* Baker f.** – Life form: Herb. Habitat: Rocky grounds in moorland, 2400–4500 m. Voucher: Mwangani 318 (EA).

***Umbilicus botryoides* Hochst. ex A.Rich.** – Life form: Herb. Habitat: Epiphytic in wet montane forest, 2100–3900 m. Voucher: Kirika et al. 19 (EA).

**F71. Cucurbitaceae**

***Cucumis ficifolius* A.Rich.** – Life form: Herbaceous climber. Habitat: Grassland, 1070–2800 m. Voucher: Ekkens 661 (EA).

***Dactyliandra stefaninii* (Chiov.) C.Jeffrey** – Life form: Herbaceous climber. Habitat: Bushlands, 500–2594 m. Vouchers: SK 0036, SK 0071 (EA, HIB).

***Diplocyclos palmatus* (L.) C.Jeffrey** – Life form: Herbaceous climber. Habitat: Moist forest and swampy grassland, 0–1830 m. Vouchers: SK 0220, SK 0066 (EA, HIB).

***Lagenaria abyssinica* (Hook.f.) C.Jeffrey** – Life form: Herbaceous climber. Habitat: Upland moist forest and riparian forest, 900–3000 m. Voucher: SK 0230 (EA, HIB).

***Momordica calantha* Gilg** – Life form: Herbaceous climber. Habitat: Moist forest margins and valley grassland, 400–1900 m. Voucher: SK 0213 (EA, HIB).

***Momordica foetida* Schumach.** – Life form: Herbaceous climber. Habitat: Moist forest edges and open sites or glades, 1200–3000 m. Voucher: SK 0211 (EA, HIB).

***Momordica friesiorum* (Harms) C.Jeffrey** – Life form: Herbaceous climber. Habitat: Upland moist forest margins and glades, 1500–2850 m. Vouchers: SK 0211, SK 0214, SK 0232, SK 0252, SK 0218, SK 0188 (EA, HIB).

***Oreosyce africana* Hook.f.** – Life form: Herbaceous climber. Habitat: Moist forest margins and bamboo thickets, 900–3000 m. Voucher: SK 0144 (EA, HIB).

***Peponium vogelii* (Hook.f.) Engl.** – Life form: Herbaceous climber. Habitat: Moist forest and bamboo thickets, 10–2600 m. Voucher: Kamau 338 (EA).

***Zehneria minutiflora* (Cogn.) C.Jeffrey** – Life form: Herbaceous climber. Habitat: Moist forest, 1100–3350 m. Voucher: Faden et al. 74/702 (EA).

***Zehneria scabra* Sond.** – Life form: Herbaceous climber. Habitat: Riverine forest and damp sites in bushland, 80–3350 m. Voucher: SAJIT 006501 (EA).

***Zehneria subcoriaceae* Y.D.Zhou & Q.F.Wang** – Life form: Herbaceous climber. Habitat: Moist montane forest, 2000–3000 m. Voucher: SK 0137 (EA, HIB).

**F72. Dichapetalaceae**

**Dichapetalum madagascariense var. brevistylum F.J.Breteler** – Life form: Woody climber. Habitat: Upland evergreen forest, 1500–2400 m. Vouchers: Luke 384, Perdue and Kibuwa 8391 (EA).

**F73. Ebenaceae**

***Diospyros abyssinica* (Hiern) F.White** – Life form: Tree. Habitat: Montane bushland, 0–2400 m. Voucher: SK 0008 (EA, HIB).

***Euclea divinorum* Hiern** – Life form: Tree. Habitat: Grassland and open bushland, 0–2700 m. Voucher: Gardner 1392 (EA).

***Agarista salicifolia* (Lam.) G.Don** – Life form: Shrub or tree. Habitat: Dry and moist forests, 1050–3500 m. Voucher: SK 0254 (EA, HIB).

**F74. Ericaceae**

***Erica arborea* L.** – Life form: Shrub. Habitat: Upper montane forest and moorland, 1600–3900 m. Voucher: Gardner 1694 (EA).

***Erica mannii* (Hook.f.) Beentje** – Life form: Shrub. Habitat: Forest clearings in hilltops, 1200–2650 m. Vouchers: Mlawton 1803, Kuchar 10283 (EA).

***Erica silvatica* (Welw. ex Engl.) Beentje** – Life form: Shrub. Habitat: Rocky grounds in moorland, 1650–4200 m. Voucher: Kokwaro 3242 (EA).

***Erica whyteana* Britten** – Life form: Shrub. Habitat: Moist sites in moorland, 1900–3650 m. Voucher: Hedberg 1511 (EA).

***Erica filago* (Alm & T.C.E.Fr.) Beentje** – Life form: Shrub. Habitat: Rocky sites in moorland, 2700–4350 m. Voucher: Kirika et al. 908 (EA).

**F75. Euphorbiaceae**

***Acalypha volkensii* Pax** – Life form: Shrub. Habitat: Forest undergrowth and bushland, 800–3000 m. Voucher: SK 0192 (EA, HIB).

***Bridelia micrantha* (Hochst.) Baill.** – Life form: Tree. Habitat: Evergreen forest and riparian forest, 0–2300 m. Voucher: SK 0034 (EA, HIB).

**Clutia abyssinica var. abyssinica Jaub. & Spach** – Life form: Shrub. Habitat: Upland bushland and wooded grassland, 1000–3700 m. Voucher: SK 0069 (EA, HIB).

**Clutia abyssinica var. usambarica Pax & K.Hoffm** – Life form: Shrub. Habitat: Dry evergreen forest, 300–2600 m. Voucher: Verdcourt 3135 (EA).

***Clutia kilimandscharica* Engl.** – Life form: Shrub. Habitat: Forest edges and bushland, 1700–3600 m. Voucher: Bally 13958 (EA).

***Croton alienus* Pax** – Life form: Shrub or small tree. Habitat: Upland dry evergreen forest, 1525–1825 m. Voucher: Kamau 319 (EA).

***Croton macrostachyus* Hochst. ex Delile** – Life form: Tree. Habitat: Forest margins and along streams, 1350–2300 m. Voucher: SK 0033 (EA, HIB).

***Croton megalocarpus* Hutch.** – Life form: Tree. Habitat: Wet and dry evergreen forest, 700–2400 m. Voucher: Kamau 319 (EA).

***Erythrococca bongensis* Pax** – Life form: Shrub. Habitat: Forest margins and bushland, 200–2440 m. Voucher: Kirika et al. 32 (EA).

***Euphorbia brevicornu* Pax** – Life form: Herb. Habitat: Moist forest, 2000–3600 m. Voucher: Napier 657 (EA).

***Euphorbia brevitorta* P.R.O.Bally** – Life form: Herb. Habitat: Dry bushland in rocky slopes, 1500–2000 m. Voucher: Kuchar 5105 (EA).

***Euphorbia depauperata* Hochst. ex A.Rich.** – Life form: Herb. Habitat: Rocky grounds in grassland and forest clearings, 1200–3350 m. Vouchers: Napier 655 (EA), SAJIT 006502 (EA, HIB).

***Euphorbia engleri* Pax** – Life form: Shrub. Habitat: Upland forest undergrowth and dense bushland, 1500–2800 m. Voucher: Hooper 1686 (EA).

***Euphorbia inaequilatera* Sond.** – Life form: Herb. Habitat: Swampy patches in upland grassland, 1990–2090 m. Voucher: Faden et al. 74/648 (EA).

***Euphorbia magnicapsula* S.Carter** – Life form: Tree. Habitat: Open deciduous bushland, 1000–2165 m. Vouchers: Kirika & Muthoka 6, Perdue and Kibuwa 8264 (EA).

***Euphorbia scarlatina* S.Carter** – Life form: Shrub. Habitat: Open deciduous bushland, 600–2000 m. Voucher: Kirika 12 (EA).

**Euphorbia schimperiana var. velutina N.E.Br.** – Life form: Herb. Habitat: Open sites in montane forests and grassland, 2970–3760 m. Voucher: Hooper et al. 1684 (EA).

***Euphorbia ugandensis* Pax & K.Hoffm.** – Life form: Shrub. Habitat: Moist forest and bamboo thicket, 1980–3350 m. Voucher: Kerfoot 478 (EA).

**Euphorbia wellbyi var. wellbyi N.E.Br.** – Life form: Shrub. Habitat: Moist upper parts montane forest zone and stream-sides in moorland, 3000–4000 m. Vouchers: Faden 74/856, Mabberley 333 (EA).

**Euphorbia wellbyi var. glabra S.Carter** – Life form: Herb. Habitat: Upper montane forest edges and heathland, 2900–4000 m. Voucher: Beentje 2625 (EA).

***Euphorbia candelabrum* Trémaux ex Kotschy** – Life form: Tree. Habitat: Open wooded grassland, 900–2180 m. Voucher: Perdue and Kibuwa 8265 (EA).

***Heywoodia lucens* Sim** – Life form: Tree. Habitat: Upland riparian forests, 1200–1950 m. Vouchers: JKCAT 1538, Seki (EA).

***Homalanthus populifolius* Graham** – Life form: Shrub to small tree. Habitat: Undergrowth in evergreen forests, 950–2100 m. Voucher: Gillett 20454 (EA).

***Macaranga capensis* (Baill.) Sim** – Life form: Tree. Habitat: Moist evergreen forest, 75–3050 m. Voucher: SK 0170 (EA, HIB).

***Macaranga kilimandscharica* Pax** – Life form: Tree. Habitat: Moist evergreen forest, 1310–3000 m. Voucher: Kuchar 5450 (EA).

***Micrococca holstii* (Pax) Prain** – Life form: Shrub. Habitat: Moist evergreen forest, 1000–2400 m. Vouchers: Battiscombe 678, Faden & Evans 70/71 (EA).

***Neoboutonia macrocalyx* Pax** – Life form: Tree. Habitat: Upland moist forest margins and forest clearings, 1100–2700 m. Voucher: Someren 3555 (EA).

**Phyllanthus boehmii var. boehmii Pax** – Life form: Herb. Habitat: Moist forest and woodland, 1050–3270 m. Vouchers: Kahurananga 2825, Verdcourt 400 (EA).

**Phyllanthus boehmii var. humilis Radcl.-Sm.** – Life form: Herb. Habitat: Damp sites in upland grassland and moorland, 2100–3250 m. Voucher: Napier 693 (EA).

***Phyllanthus fischeri* Pax** – Life form: Shrub. Habitat: Forest edges and along seasonal streams, 1450–2960 m. Voucher: Kamau 320 (EA).

***Tragia brevipes* Pax** – Life form: Herb or subshrub. Habitat: Forest edges and riverine vegetation, 600–2600 m. Voucher: SK 0157 (EA, HIB).

***Tragiella natalensis* (Sond.) Pax & K.Hoffm.** – Life form: Herb. Habitat: Forest edges and associated bushland, 80–2300 m. Voucher: Verdcourt 546 (EA).

*Vernicia fordii (Hemsl.) Airy Shaw – Life form: Exotic tree. Habitat: Cultivated along moist forest edges. Voucher: Patterson 324/58 (EA).

**F76. Fabaceae**

***Acacia abyssinica* Benth.** – Life form: Tree. Habitat: Woodland and wooded grassland, 1500–2300 m. Voucher: Verdcourt 1501 (EA).

***Acacia xanthophloea* Benth.** – Life form: Tree. Habitat: Riverine forest, 600–1980 m. Voucher: Ahiti 131 (EA).

***Adenocarpus mannii* (Hook.f.) Hook.f.** – Life form: Shrub. Habitat: Moorland, 1500–4000 m. Voucher: SK 0113 (EA, HIB).

***Aeschynomene schimperi* A.Rich.** – Life form: Shrub. Habitat: Swampy areas and along streams, 60–2340 m. Voucher: Battiscombe 193 (EA).

***Albizia gummifera* (J.F.Gmel.) C.A.Sm.** – Life form: Tree. Habitat: Moist forest, 0–2440 m. Voucher: Poster (EA).

***Amphicarpaea africana* (Hook.f.) Harms** – Life form: Herbaceous climber. Habitat: Upland moist forest and bamboo zone, 1680–2700 m. Voucher: Battiscombe 298 (EA).

***Argyrolobium friesianum* (Hook.f.) Harms** – Life form: Herb or subshrub. Habitat: Margins of upland moist forest, 1800–3000 m. Voucher: Mbale et al. 847 (EA).

**Argyrolobium rupestre subsp. aberdaricum (Harms) Polhill** – Life form: Herb. Habitat: Upland grassland and moorland, 1900–3500 m. Voucher: Bally 2756 (EA).

**Argyrolobium rupestre subsp. kilimandscharicum (Taub.) Polhill** – Life form: Herb. Habitat: Upland grassland, 2250–3700 m. Voucher: Sir Charles 10607 (EA).

**Astragalus atropilosulus var. astropilosulus (Hochst.) Bunge** – Life form: Herb. Habitat: Upland grassland and forest margins, 1200–4200 m. Voucher: Beentje 2452 (EA).

**Astragalus atropilosulus subsp. bequaertii (De Wild.) J.B.Gillett** – Life form: Herb. Habitat: Roadsides in grassland, 1200–4200 m. Voucher: Kerfoot 402 (EA).

**Astragalus atropilosulus subsp. burkeanus (Harvey) J.B.Gillett** – Life form: Herb. Habitat: Bushland margins and open sites in bamboo forest, 2100–2700 m. Voucher: Verdcourt 691 (EA).

*Caesalpinia decapetala (Roth) Alston – Life form: Exotic woody climber. Habitat: Open sites in montane forest and bushland, 880–2200 m. Voucher: Ament et al. 121 (EA).

***Calpurnia aurea* (Aiton) Benth.** – Life form: Tree. Habitat: Upland rainforest margins and riverine forest, 1300–2260 m. Voucher: Kirika et al. 881 (EA).

***Chamaecrista hildebrandtii* (Vatke) Lock** – Life form: Woody herb. Habitat: Wooded grassland, 1470–2300 m. Voucher: Faden 67419 (EA).

***Chamaecrista stricta* E.Mey.** – Life form: Woody herb. Habitat: Roadsides in open bushland, 880–2040 m. Voucher: Whyte (EA).

***Chamaecrista usambarensis* (Taub.) Standl.** – Life form: Herb. Habitat: Rocky grounds in upland grassland, 1760–2590 m. Voucher: Yyne-Watt 1187 (EA).

**Crotalaria agatiflora subsp. agatiflora Schweinf.** – Life form: Herb. Habitat: Upland grassland and deciduous bushland, 1500–3150 m. Voucher: SK 0051 (EA, HIB).

**Crotalaria agatiflora subsp. engleri (Baker f.) Polhill.** – Life form: Herb. Habitat: Upland forest margins and riverine forest, 1500–3500 m. Voucher: Kimani 16 (EA).

***Crotalaria axillaris* Aiton** – Life form: Shrub. Habitat: Forest margins and deciduous woodland, 0–2500 m. Voucher: Perdue 8064 (EA).

**Crotalaria brevidens var. parviflora (Baker f.) Polhill** – Life form: Herb. Habitat: Upland dry evergreen forest, 1500–3000 m. Vouchers: Verdcourt 2920, Strange 286 (EA).

***Crotalaria fascicularis* Polhill** – Life form: Shrub. Habitat: Margins of upland rainforest, 1950–2950 m. Voucher: Gilbert 4896 (EA).

**Crotalaria incana var. purpurascens (Lam.) Milne-Redh.** – Life form: Herb. Habitat: Upland grassland, 1050–2600 m. Vouchers: Kokwaro 346, Kuchar 12255 (EA).

***Crotalaria jacksonii* Baker f.** – Life form: Shrub. Habitat: Upland grassland and margins of moist forest, 2200–3000 m. Voucher: Kerfoot 4590 (EA).

***Crotalaria keniensis* Baker f.** – Life form: Shrub. Habitat: Moist forest margins and clearings, 1500–2850 m. Voucher: Becky 2175 (EA).

***Crotalaria lebrunii* Baker f.** – Life form: Shrub. Habitat: Moist forest margins and clearings, 1350–2760 m. Voucher: Mathenge 206 (EA).

***Crotalaria mauensis* Baker f.** – Life form: Shrub. Habitat: Upland forest margins, 1600–2800 m. Voucher: Verdcourt 681 (EA).

***Crotalaria natalitia* Meissner** – Life form: Woody herb or shrub. Habitat: Riverine forest and upland moist forest, 0–3000 m. Voucher: Kerfoot 481 (EA).

***Crotalaria prittwitzii* Baker f.** – Life form: Woody herb or shrub. Habitat: Riverine forest and upland moist forest, 0–3000 m. Voucher: Napper 637 (EA).

***Crotalaria pseudospartium* Baker f.** – Life form: Shrub. Habitat: Upland wooded grassland, 1400–2500 m. Voucher: Verdcourt 3571 (EA).

***Crotalaria rhizoclada* Polhill** – Life form: Shrub. Habitat: Roadsides in upland grassland, 1200–2500 m. Voucher: Lacey (EA).

***Crotalaria tabularis* Baker f.** – Life form: Shrub. Habitat: Margins of upland rainforest, 1200–3000 m. Voucher: Mainwaring 2407 (EA).

**Dolichos sericeus subsp. glabrescens Verdc.** – Life form: Herb. Habitat: Upland bushland and dry evergreen forest, 1200–2780 m. Voucher: SK 0225 (EA, HIB).

**Dolichos sericeus subsp. pseudofalcatus Verdc.** – Life form: Herb. Habitat: Upland grassland, 1200–2780 m. Voucher: Someren 1151 (EA).

**Eriosema scioanum subsp. lejeunei (Staner & De Craene) Verdc.** – Life form: Herb. Habitat: Upland forest glades and grassland, 1500–2580 m. Voucher: Verdcourt 1605 (EA).

***Erythrina abyssinica* DC.** – Life form: Tree. Habitat: Scattered-tree grassland, 200–2100 m. Vouchers: Kirika 163, 157 (EA).

***Hylodesmum repandum* (Vahl) H.Ohashi & R.R.Mill** – Life form: Herb. Habitat: Shaded grounds in upland moist forest, 1000–3000 m. Voucher: Kerfoot 473 (EA).

***Indigastrum costatum* (Guill. & Perr.) Schrire** – Life form: Herb. Habitat: Short grassland, 500–1900 m. Voucher: Bally 955 (EA).

***Indigofera arrecta* A.Rich.** – Life form: Woody herb. Habitat: Bushland and forest edges, 300–2700 m. Voucher: Kerfoot 392 (EA).

***Indigofera atriceps* Hook.f.** – Life form: Herb. Habitat: Upland grassland and forest margins, 1000–3200 m. Vouchers: Kerfoot 1443 (EA), SK 0041 (EA, HIB).

***Indigofera circinella* Baker f.** – Life form: Woody herb. Habitat: Grassland, 50–2200 m. Voucher: Napier 1794 (EA).

***Indigofera demissa* Taub.** – Life form: Herb. Habitat: Roadsides in grassland, 900–2500 m. Voucher: Nattras 1285 (EA).

**Indigofera nairobiensis subsp. nairobiensis Baker f.** – Life form: Woody herb. Habitat: Upland grassland, 1900–2300 m. Voucher: Bogdan 4624 (EA).

**Indigofera nairobiensis subsp. vicida J.B.Gillett** – Life form: Woody herb. Habitat: Upland grassland, 1900–2300 m. Voucher: Gillet 19364 (EA).

***Indigofera swaziensis* Bolus** – Life form: Shrub. Habitat: Upland evergreen forest margins, 1200–2700 m. Voucher: Hansen 762 (EA).

**Indigofera trita subsp. scabra (Roth) Ali** – Life form: Woody herb. Habitat: Grassland and bushland, 0–2500 m. Voucher: Malombe et al. 1381 (EA).

**Kotschya recurvifolia subsp. keniensis Verdc.** – Life form: Shrub. Habitat: Dry evergreen bushland, 2340–3000 m. Vouchers: Gardner 1135, Dale 2684 (EA).

***Lathyrus hygrophilus* Taub.** – Life form: Herbaceous climber. Habitat: Wet sites in moorland and bamboo forest, 1800–4100 m. Voucher: Mabberley 377 (EA).

***Lotus becquetii* Boutique** – Life form: Herb. Habitat: Upland grassland, 2000–3200 m. Voucher: Hawery 168B (EA).

***Lotus corniculatus* L.** – Life form: Herb. Habitat: Wet sites in upland grassland, 1400–2700 m. Voucher: Allnechtsen 10 (EA).

***Lotus goetzei* Harms** – Life form: Herb. Habitat: Upland forest edges and grassland, 1500–3700 m. Voucher: Napier 689 (EA).

***Medicago lupulina* L.** – Life form: Herb. Habitat: Upland grassland, 1800–2900 m. Voucher: Kirika et al. 77 (EA).

***Melilotus officinalis* (L.) Pall.** – Life form: Herb. Habitat: Roadsides in grassland, 2000–2000 m. Voucher: Kulkarni 14116 (EA).

***Neonotonia wightii* (Wight & Arn.) J.A.Lackey** – Life form: Woody climber. Habitat: Grassland and bushland, 0–2500 m. Voucher: Fries 537 (EA).

***Ormocarpum trachycarpum* (Taub.) Harms** – Life form: Shrub or small tree. Habitat: Grassland and woodland, 950–1800 m. Voucher: Perdue and Kibuwa 8263 (EA).

***Otholobium foliosum* (Oliv.) C.H.Stirt.** – Life form: Shrub. Habitat: Upland grassland and forest margins, 1200–3200 m. Voucher: Fries 1581 (EA).

***Parochetus communis* D.Don** – Life form: Herb. Habitat: Upland moist forest and bamboo forest, 1500–3450 m. Voucher: Kuchar and Msafiri 5442 (EA).

**Rhynchosia congensis subsp. orientalis Verdc.** – Life form: Herb. Habitat: Grassland, 45–2280 m. Voucher: SK 0022 (EA, HIB).

**Rhynchosia densiflora subsp. stuhlmannii (Harms) Verdc.** – Life form: Herbaceous climber. Habitat: Upland grassland with scattered trees, 1200–2160 m. Vouchers: Nattrass 375, 588 (EA).

***Rhynchosia hirta* (Andrews) Meikle & Verdc.** – Life form: Herb. Habitat: Grassland and forest edges, 0–1850 m. Voucher: KEFRI & Omondi 106 (EA).

**Rhynchosia minima var. prostrata (Harv.) Meikle.** – Life form: Herb. Habitat: Grassland, 45–2280 m. Vouchers: Verdcourt 1606, Agnew 7685 (EA).

**Rhynchosia usambarensis subsp. inelegans Verdc.** Life form: Woody herb. Habitat: Upland forest edges and bushland, 1200–2400 m. Vouchers: Napier 2454, Robertson 1545 (EA).

***Senna didymobotrya* (Fresen.) H.S.Irwin & Barneby** – Life form: Shrub. Habitat: Moist forest edges and riverine forest, 1500–2250 m. Voucher: SK 0209 (EA, HIB).

*Senna septemtrionalis (Viv.) H.S.Irwin & Barneby – Life form: Exotic shrub. Habitat: Dry or moist forest, 910–3200 m. Vouchers: Mainwaring 2192 (EA) SK 0030 (EA, HIB).

***Senna singueana* (Delile) Lock** – Life form: Shrub or small tree. Habitat: Wooded grassland, 0–2130 m. Voucher: Perdue and Kibuwa 8216 (EA).

***Stylosanthes fruticosa* (Retz.) Alston** – Life form: Herb or subshrub. Habitat: Grassland and bushland, 10–2720 m. Voucher: Perdue and Kibuwa 8183 (EA).

***Trifolium burchellianum* Ser.** – Life form: Herb. Habitat: Clearings in upland forest and bamboo thickets, 1600–3980 m. Voucher: SK 0063 (EA, HIB).

***Trifolium cryptopodium* Steud. ex A.Rich.** – Life form: Herb. Habitat: Moist forest clearings and moorland, 1800–4200 m. Voucher: Gillett 19289 (EA).

***Trifolium lanceolatum* (J.B.Gillett) J.B.Gillett** – Life form: Herb. Habitat: Upland grassland, 1950–2800 m. Voucher: Achlactoe 2749 (EA).

***Trifolium multinerve* A.Rich.** – Life form: Herb. Habitat: Moist upland grassland and moorland, 1800–3700 m. Voucher: Mabberley 352 (EA).

***Trifolium polystachyum* Fresen.** – Life form: Herb. Habitat: Moist forest margins, 1600–2800 m. Voucher: Meinertzhagen (EA).

**Trifolium semipilosum var. semipilosum Fresen.** – Life form: Herb. Habitat: Upland grassland, 1500–3000 m. Voucher: Beentje 320g (EA).

**Trifolium semipilosum var. glabrescens J.B.Gillett** – Life form: Herb. Habitat: Upland grassland, 1200–2700 m. Vouchers: Trapnell 2111, Scott Elliot 6606 (EA).

***Trifolium simense* Fresen.** – Life form: Herb. Habitat: Upland grassland, 1500–3100 m. Voucher: Muasya et al. 002 (EA).

***Trifolium steudneri* Schweinf.** – Life form: Herb. Habitat: Upland grassland and forest margins, 1800–2400 m. Voucher: Bogdan 3222 (EA).

***Trifolium tembense* Fresen.** – Life form: Herb. Habitat: Wet places in upland forest and moorland, 2000–3800 m. Voucher: Polhill 438 (EA).

*Vicia benghalensis L. – Life form: Exotic herb. Habitat: Disturbed areas in upland forest, 2400–2800 m. Voucher: Agriculture Dept 11568 (EA).

***Vicia hirsuta* (L.) Gray** – Life form: Herb. Habitat: Upland forest glades and grassland, 1950–3360 m. Voucher: Bogdan 1985 (EA).

***Vicia sativa* L.** – Life form: Herb. Habitat: Upland grassland, 1700–3350 m. Voucher: Mabberley 376 (EA).

*Vicia villosa subsp. varia (Host) Corb. – Life form: Exotic herb. Habitat: Upland grassland, 1860–2700 m. Vouchers: Agric. Dept 11570, Blacklands 16293 (EA).

***Vigna luteola* (Jacq.) Benth.** – Life form: Herbaceous climber. Habitat: Swampy forest and wet grassland, 650–1920 m. Vouchers: Battiscombe 1123, Kirrika 230, Napper 412 (EA).

**Vigna membranacea subsp. macrodon (Robyns & Boutique) Verdc.** – Life form: Herbaceous climber. Habitat: Upland moist forest, 1275–2100 m. Vouchers: Verdcourt 661, EANHS KF/77 (EA).

***Vigna parkeri* Baker** – Life form: Herbaceous climber. Habitat: Upland grassland with scattered trees, 1050–2900 m. Voucher: Kerfoot 1369 (EA).

**F77. Gentianaceae**

***Sebaea brachyphylla* Griseb.** – Life form: Herb. Habitat: Moist montane forests and moorlands, 1400–3470 m. Voucher: SK 0118 (EA, HIB).

***Sebaea leiostyla* Gilg** – Life form: Herb. Habitat: Stream-sides and marshes in upland grassland, 1800–3500 m. Voucher: Kerfoot 1369 (EA).

**Sebaea pentandra var. burchellii E.Mey.** – Life form: Herb. Habitat: Stream-sides and marshes in upland grassland, 1450–2450 m. Voucher: Greenway 13569 (EA).

***Swertia crassiuscula var. crassiuscula* Gilg** – Life form: Herb. Habitat: Moorland, 2700–4500 m. Vouchers: Copley 138 (EA), SK 0087 (EA, HIB).

**Swertia crassiuscula var. leucantha (T.C.E.Fr.) Sileshi** – Life form: Herb. Habitat: Moorland, 2700–4500 m. Voucher: Dowson 101 (EA).

***Swertia eminii* Engl.** – Life form: Herb. Habitat: Wet grassland and swamp margins, 1200–2250 m. Voucher: Paulo et al. 888 (EA).

***Swertia kilimandscharica* Engl.** – Life form: Herb. Habitat: Open sites in montane forest, 2100–3840 m. Voucher: Bally 8643 (EA).

***Swertia lugardiae* Bullock** – Life form: Herb. Habitat: Montane grassland, 2450–3550 m. Voucher: Naper 1231 (EA).

***Swertia volkensii* Gilg** – Life form: Herb. Habitat: Upper parts of montane forest to the moorland, 2800–4250 m. Voucher: Hedberg 1544 (EA).

**F78. Geraniaceae**

***Geranium aculeolatum* Oliv.** – Life form: Herb. Habitat: Upland rainforest, 1200–3400 m. Voucher: Mathenge 220 (EA).

**Geranium arabicum subsp. arabicum Forssk.** – Life form: Herb. Habitat: Rainforest and moist sites in grassland and moorland, 1100–3940 m. Voucher: SAJIT 006498 (EA, HIB).

**Geranium arabicum subsp. latistipulatum (Hochst. ex A.Rich.) Kokwaro** – Life form: Herb. Habitat: Upland grassland and bushland, 1100–2800 m. Vouchers: Mwangangi 989, Kokwaro 32 (EA).

***Geranium kilimandscharicum* Engl.** – Life form: Herb. Habitat: Rocky sites in moorland, 2260–4300 m. Voucher: SK 0059 (EA, HIB).

***Geranium mascatense* Boiss.** – Life form: Herb. Habitat: Upland grassland and evergreen bushland, 1000–2900 m. Voucher: Verdcourt 679 (EA).

***Geranium ocellatum* Cambess.** – Life form: Herb. Habitat: Upland wooded grassland and evergreen bushland, 1000–2900 m. Voucher: Verdcourt 679 (EA).

***Geranium purpureum* Vill.** – Life form: Herb. Habitat: Upland rainforest and riverine forest, 1300–3000 m. Vouchers: Napier 729, Pierce 1682 (EA).

**Geranium vagans subsp. vagans Baker** – Life form: Herb. Habitat: Upland grassland and moorland, 1370–4500 m. Vouchers: SAJIT 006478, SK 0108 (EA, HIB).

**Geranium vagans subsp. whytei (Baker) J.R.Laundon** – Life form: Herb. Habitat: Upland grassland and moorland, 1370–4500 m. Voucher: Kuchar 12495 (EA).

***Pelargonium alchemilloides* (L.) Aiton** – Life form: Herb. Habitat: Bushland and montane forest edges, 700–2800 m. Voucher: Townsend 2319 (EA).

***Pelargonium inquinans* (L.) L’Her.** – Life form: Herb. Habitat: Bushland and wooded grassland, 700–2800 m. Voucher: SK 0269 (EA, HIB).

**F79. Gesneriaceae**

***Streptocarpus glandulosissimus* Engl.** – Life form: Herb. Habitat: Moist forest, 900–2600 m. Voucher: Bally 8517 (EA).

**F80. Gunneraceae**

***Gunnera perpensa* L.** – Life form: Herb. Habitat: Upland riparian forest, 1560–4000 m. Voucher: Gardner 1882 (EA).

**F81. Hamamelidaceae**

***Trichocladus ellipticus* Eckl. & Zeyh.** – Life form: Tree. Habitat: Upland moist forest, 1350–2800 m. Voucher: Hansen 808 (EA).

**F82. Hypericaceae**

***Hypericum kiboense* Oliv.** – Life form: Shrub. Habitat: Upland dry evergreen forests and grassland, 2100–3900 m. Voucher: Battiscombe 710 (EA).

***Hypericum lalandii* Choisy** – Life form: Herb. Habitat: Marshes and damp sites in upland grassland, 1080–2250 m. Voucher: Taylor 1511 (EA).

***Hypericum lanceolatum* Lam.** – Life form: Shrub or small tree. Habitat: Upland dry evergreen forests, 1800–3360 m. Voucher: Edwards 2843/16 (EA).

***Hypericum peplidifolium* A.Rich.** – Life form: Herb. Habitat: Wet places in moorland, 1170–3600 m. Voucher: Coe 788 (EA).

**Hypericum revolutum subsp. revolutum Vahl** – Life form: Shrub. Habitat: Upland dry evergreen forest, 2100–3250 m. Vouchers: SAJIT 006484, SK 0117 (EA, HIB).

**Hypericum revolutum subsp. keniense (Schweinf.) N.Robson** – Life form: Shrub. Habitat: Dry evergreen forest, 2700–3800 m. Voucher: Knox 3722 (EA).

***Hypericum scioanum* Chiov.** – Life form: Herb. Habitat: Wet sites in moorland, 1830–3590 m. Voucher: Mabberley 344 (EA).

**F83. Icacinaceae**

***Apodytes dimidiata* E.Mey. ex Arn.** – Life form: Tree. Habitat: Upland moist forest, 1000–2500 m. Voucher: SK 0146 (EA, HIB).

**F84. Lamiaceae**

***Achyrospermum schimperi* (Hochst. ex Briq.) Perkins** – Life form: Herb. Habitat: Montane forest undergrowth, 1200–3000 m. Voucher: SK 0208 (EA, HIB).

***Ajuga integrifolia* Buch.-Ham. ex D.Don** – Life form: Herb. Habitat: Upland moist forest, 1000–3400 m. Voucher: SK 0197 (EA, HIB).

***Clerodendrum johnstonii* Oliv.** – Life form: Woody climber. Habitat: Upland moist forest, 1200–2550 m. Voucher: Verdcourt 3780 (EA).

**Clinopodium abyssinicum var. condensatum (Hedberg) Ryding** – Life form: Shrub. Habitat: Wet evergreen bushland and grassland, 1000–3950 m. Vouchers: Lind 2934, Agricultural Dept 62 (EA).

***Clinopodium kilimandschari* (Gürke) Ryding** – Life form: Herb. Habitat: Moorland and heath zone, 2900–4400 m. Voucher: SK 0090 (EA, HIB).

***Clinopodium simense* (Benth.) Kuntze** – Life form: Herb. Habitat: Moist grassland and open woodland, 1700–3500 m. Voucher: Fries and Fries 2429 (EA).

***Clinopodium uhligii* (Gürke) Ryding** – Life form: Shrub. Habitat: Montane forest edges and evergreen bushland, 3350–4180 m. Voucher: Kuchar 10360 (EA).

***Fuerstia africana* T.C.E.Fr.** – Life form: Woody herb. Habitat: Upland grassland, 1200–2550 m. Voucher: Kuchar 8357a (EA).

***Leonotis nepetifolia* (L.) R.Br.** – Life form: Herb. Habitat: Upland grassland and bushland, 1000–2290 m. Voucher: Napier 2587 (EA).

**Leonotis ocymifolia var. raineriana (Vis.) Iwarsson.** – Life form: Shrub. Habitat: Margins of montane forest, 600–3700 m. Vouchers: Verdcourt 3813 (EA), SK 0097 (EA, HIB).

***Leucas deflexa* Hook.f.** – Life form: Herb. Habitat: Moist forest, 1000–2500 m. Voucher: Gilbert 6312 (EA).

***Leucas grandis* Vatke** – Life form: Herb or subshrub. Habitat: Upland evergreen forest, 500–2780 m. Voucher: Kamau 367 (EA).

***Leucas masaiensis* Oliv.** – Life form: Herb. Habitat: Upland forest glades and margins, 1300–3200 m. Voucher: Kirika et al. 163 (EA).

**Leucas masaiensis var. venulosa (Baker) Sebald** – Life form: Herb. Habitat: Upland grassland, 1300–3200 m. Voucher: Faden et al. 74/577 (EA).

**Leucas oligocephala var. oligocephala Hook.f.** Life form: Herb. Habitat: Upland grassland and forest margins, 1600–2990 m. Vouchers: Verdcourt 1010, 1023 (EA).

**Leucas volkensii var. parviflora Sebald.** – Life form: Herb. Habitat: Open sites in montane forest, 2000–2600 m. Vouchers: Taylor 1237, Beentje 3254 (EA).

***Mentha aquatica* L.** – Life form: Herb. Habitat: Upland marshes, 1100–2150 m. Voucher: Kuchar 9573 (EA).

***Mentha longifolia* (L.) L.** – Life form: Herb. Habitat: Upland grassland and marshes, 1650–2450 m. Vouchers: MacDonald 1344, Poster 3234 (EA).

**Micromeria imbricata var. imbricata (Forssk.) C.Chr.** – Life form: Woody herb. Habitat: Upland open woodland and dry grassland, 1200–4000 m. Voucher: Napier 1761 (EA).

**Micromeria imbricata var. villosa (Elly Walther & K.H. Walther) Ryding** – Life form: Woody herb. Habitat: Upland evergreen bushland and grassland, 1850–4100 m. Voucher: Dawson 415 (EA).

***Nepeta azurea* R.Br. ex Benth.** – Life form: Herb. Habitat: Upland evergreen bushland and grassland, 1700–3800 m. Voucher: SK 0120 (EA, HIB).

***Ocimum decumbens* Gürke** – Life form: Shrub. Habitat: Grassland, 950–4000 m. Voucher: Lind 3138 (EA).

***Ocimum gratissimum* L.** – Life form: Herb. Habitat: Dry montane forest, 1100–2400 m. Voucher: Perdue and Kibuwa 8046 (EA).

***Ocimum kenyense* Ayob. ex A.J.Paton** – Life form: Herb. Habitat: Wet places in grassland, 1050–2300 m. Voucher: Mainwaring 2406 (EA).

***Ocimum kilimandscharicum* Gürke** – Life form: Shrub. Habitat: Upland grassland, 1100–2350 m. Voucher: Faden 68/721 (EA).

***Ocimum lamiifolium* Hochst. ex Benth.** – Life form: Shrub. Habitat: Upland forest edges and bushland, 1000–2500 m. Voucher: Kokwaro et al. 2344 (EA).

***Platostoma denticulatum* Robyns** – Life form: Herb. Habitat: Damp sites in grassland and open woodland, 100–2480 m. Voucher: Kerfoot 639 (EA).

***Plectranthus alboviolaceus* Gürke** – Life form: Shrub. Habitat: Upland moist forest and along rivers, 1500–2800 m. Voucher: Kimani 22 (EA).

***Plectranthus alpinus* (Vatke) Ryding** – Life form: Shrub. Habitat: Moist forest and along streams, 1900–2750 m. Voucher: Napper 657 (EA).

***Plectranthus caespitosus* Lukhoba & A.J.Paton** – Life form: Herb. Habitat: Upland grassland, 1500–2850 m. Voucher: Someren 28 (EA).

***Plectranthus kamerunensis* Gürke** – Life form: Herb. Habitat: Glades in moist forest and bamboo zone, 1200–2700 m. Voucher: Young 1021 (EA).

***Plectranthus laxiflorus* Benth.** – Life form: Herb. Habitat: Glades in moist forest and bamboo forest, 1600–3120 m. Voucher: Alexander 11636 (EA).

***Plectranthus longipes* Baker** – Life form: Herb or subshrub. Habitat: Rocky grounds in bushland and woodland, 700–2440 m. Voucher: Patel 164 (EA).

***Plectranthus melleri* Baker** – Life form: Shrub. Habitat: Open areas in moist montane forest, 1300–2400 m. Voucher: Kokwaro and Mathenge 2981 (EA).

***Plectranthus mollis* (Aiton) Spreng.** – Life form: Herb. Habitat: Upland grassland and forest margins, 1200–2900 m. Voucher: SK 0231 (EA, HIB).

***Plectranthus montanus* Benth.** – Life form: Herb. Habitat: Dry evergreen forest, 500–2400 m. Voucher: Bally 2602 (EA).

***Plectranthus parvus* Oliv.** – Life form: Herb. Habitat: Upland grassland and forest margins, 1200–2900 m. Voucher: Faden 67/838 (EA).

**Plectranthus punctatus subsp. edulis (Vatke) A.J.Paton** – Life form: Herb. Habitat: Moist montane forest up to bamboo zone, 1800–3200 m. Vouchers: Kerfoot 51, Mwangangi 977 (EA).

***Plectranthus sylvestris* Gürke** – Life form: Shrub. Habitat: Moist montane forest up to bamboo zone, 1750–3280 m. Voucher: Agnew 7705 (EA).

**Rotheca myricoides var. myricoides (Hochst.) Steane & Mabb.** – Life form: Shrub. Habitat: Grassland and open woodland, 900–2400 m. Voucher: Faden 74/570 (EA).

**Rotheca myricoides var. discolor (Klotzsch) Verdc.** – Life form: Shrub. Habitat: Grassland and open woodland, 900–2400 m. Voucher: Hansen 781 (EA).

***Salvia merjamie* Forssk.** – Life form: Herb. Habitat: Montane grassland and moorland, 2250–4100 m. Voucher: Mbale et al. 861 (EA).

*Salvia nilotica Juss. ex Jacq. – Life form: Exotic herb. Habitat: Montane grassland and forest edges, 1350–3700 m. Voucher: Harvey 179 (EA).

***Stachys aculeolata* Hook.f.** – Life form: Herb. Habitat: Moist sites in bamboo zone and moorland, 1400–3650 m. Voucher: Napier 698 (EA).

***Stachys alpigena* T.C.E.Fr.** – Life form: Herb. Habitat: Montane moorland and ericaceous zone, 2900–3750 m. Vouchers: Kokwaro 1908 (EA), SK 0064 (EA, HIB).

***Stachys argillicola* Sebsebe** – Life form: Woody herb. Habitat: Upland grassland, 1660–2200 m. Voucher: Evans 60/167 (EA).

***Tinnea aethiopica* Kotschy ex Hook.f.** – Life form: Herb. Habitat: Wooded grassland, 0–2300 m. Voucher: Faden 74/578 (EA).

***Vitex keniensis* Turrill** – Life form: Tree. Habitat: Moist evergreen forest, 1290–2100 m. Voucher: SK 0248 (EA, HIB).

**F85. Lauraceae**

***Ocotea kenyensis* (Chiov.) Robyns & R.Wilczek** – Life form: Tree. Habitat: Moist montane forest, 1140–2400 m. Voucher: Elliot 2356 (EA).

***Ocotea usambarensis* Engl.** – Life form: Tree. Habitat: Moist montane forest, 900–3000 m. Voucher: Forest Dept. 176 (EA).

**F86. Lentibulariaceae**

***Utricularia gibba* L.** – Life form: Herb. Habitat: Aquatic in shallow flowing water and freshwater pools, 10–2550 m. Vouchers: William 12346 & 12347 (EA).

***Utricularia livida* E.Mey.** – Life form: Herb. Habitat: Wet grassland, 0–2730 m. Voucher: Gilbert 4868 (EA).

**F87. Linaceae**

***Linum keniense* T.C.E.Fr.** – Life form: Herb. Habitat: Upland grassland and open grounds in bamboo thickets, 2200–3360 m. Vouchers: SAJIT 006471 & 006489 (EA, HIB).

***Linum volkensii* Engl.** – Life form: Herb. Habitat: Wet grassland and stream banks, 1300–2750 m. Voucher: Symes 131 (EA).

**F88. Loganiaceae**

***Buddleja polystachya* Fresen.** – Life form: Shrub. Habitat: Margins and clearings in upland rainforest, 1000–2700 m. Voucher: Birch 61/31 (EA).

***Nuxia congesta* R.Br. ex Fresen** – Life form: Tree. Habitat: Upland rainforest, 1550–2850 m. Voucher: Kokwaro 4421 (EA).

**F89. Loranthaceae**

***Agelanthus brunneus* (Engl.) Balle & N.Hallé** – Life form: Shrub. Habitat: Moist evergreen forest and riparian forest, 1000–1800 m. Voucher: Someren 3191 (EA).

***Agelanthus pennatulus* (Sprague) Polhill & Wiens** – Life form: Shrub. Habitat: Moist montane forest, 1650–2400 m. Voucher: Kuchar and Msafiri 5461 (EA).

**Agelanthus sansibarensis subsp. montanus Polhill & Wiens** – Life form: Shrub. Habitat: Moist montane forest, 0–2500 m. Vouchers: Wiens 4564, Mainwaring s.n. (EA).

***Agelanthus subulatus* (Engl.) Polhill & Wiens** – Life form: Shrub. Habitat: Bushland and wooded grassland, 10–2300 m. Voucher: Faden & Evans 74/706 (EA).

***Englerina woodfordioides* (Schweinf.) Balle** – Life form: Shrub. Habitat: Moist montane forest and riverine forest, 1350–3050 m. Vouchers: SK 0070, SK 0240 (EA, HIB).

***Oncocalyx sulfureus* (Engl.) Wiens & Polhill** -Life form: Herb. Habitat: Epiphytic in upland dry evergreen forest, 1700–3000 m. Voucher: Hepper 4916 (EA).

**F90. Lythraceae**

*Parsonsia micropetala (Kunth) Standl. – Life form: Exotic shrub. Habitat: Escaped cultivation common on stream-sides and disturbed sites. Voucher: Hooper and Townsend 1691 (EA).

***Lythrum rotundifolium* Hochst. ex A.Rich.** – Life form: Herb. Habitat: Upland water pools and swamps, 1650–3300 m. Voucher: Kahurananga et al. 2820 (EA).

**Nesaea kilimandscharica var. ngongensis Verdc.** – Life form: Woody herb or shrub. Habitat: Upland grassland and bushland, 1650–2130 m. Vouchers: Hansen 816, Gillett 17342 (EA).

**Nesaea schinzii subsp. subalata (Koehne) Verdc.** – Life form: Herb or subshrub. Habitat: Scattered-tree grassland, 1080–2100 m. Vouchers: Dowson 541, Dowson 541 (EA).

**F91. Malvaceae**

**Abutilon longicuspe var. longicuspe Hochst. ex A.Rich.** – Life form: Shrub. Habitat: Open sites in dry evergreen forest, 1650–3300 m. Voucher: Faden 67719 (EA).

**Abutilon longicuspe var. pilosicalyx Verdc.** – Life form: Shrub. Habitat: Upland grassland and dry forest margins, 1700–2400 m. Voucher: Dyson 456 (EA).

***Abutilon mauritianum* (Jacq.) Medik.** – Life form: Shrub. Habitat: Woodland and forest edges, 0–2300 m. Voucher: Kokwaro & Kabuye 329 (EA).

***Dombeya kirkii* Mast.** – Life form: Shrub or small tree. Habitat: Bushland and forest margins, 600–2400 m. Voucher: SK 0132 (EA, HIB).

***Dombeya rotundifolia* (Hochst.) Planch.** – Life form: Tree. Habitat: Forest edges and wooded grassland, 1000–2400 m. Voucher: Someren 692 (EA).

***Dombeya torrida* (J.F.Gmel.) Bamps** – Life form: Tree. Habitat: Upland open forests and forest margins, 1700–3050 m. Voucher: Brasnett 200 (EA).

***Grewia similis* K.Schum.** – Life form: Shrub. Habitat: Woodland and grassland, 600–2250 m. Voucher: Fukuoka 106 (EA).

***Hibiscus fuscus* Garcke** – Life form: Woody herb. Habitat: Upland bush thickets and grassland, 1400–2650 m. Voucher: Kokwaro & Kabuye 340 (EA).

***Hibiscus macranthus* Hochst. ex A.Rich.** – Life form: Shrub. Habitat: Upland forest edges and bushland, 1500–2900 m. Voucher: Kuchar 12297 (EA).

**Hibiscus vitifolius subsp. vitifolius Brenan & Exell** – Life form: Shrub. Habitat: Dry bushland, 420–3000 m. Vouchers: Robertson 1805, McDonald 922 (EA).

***Malva verticillata* L.** – Life form: Herb. Habitat: Moist montane forest, 1200–4050 m. Voucher: McDonald 1285 (EA).

***Pavonia burchellii* (DC.) R.A.Dyer** – Life form: Shrub. Habitat: Woodland and rainforest margins, 750–2300 m. Voucher: SK 0203 (EA, HIB).

***Pavonia schimperiana* Hochst. ex A.Rich.** – Life form: Woody herb or shrub. Habitat: Upland short grassland and forest edges, 1100–2400 m. Voucher: Smith et al. 66 (EA).

***Pavonia urens* Cav.** – Life form: Shrub. Habitat: Forest margins and riparian vegetation, 600–3000 m. Vouchers: SK 0020, SK 0068, SK 0261 (EA, HIB).

***Sida cordifolia* L.** – Life form: Herb or subshrub. Habitat: Upland bushland, 1300–2291 m. Voucher: SK 0163 (EA, HIB).

***Sida rhombifolia* L.** – Life form: Shrub. Habitat: Open woodland, 900–2250 m. Voucher: SK 0242 (EA, HIB).

***Sida schimperiana* Hochst. ex A.Rich.** – Life form: Shrub. Habitat: Upland grassland, 1200–2700 m. Voucher: Smith et al. 66 (EA).

***Sida tenuicarpa* Vollesen** – Life form: Shrub. Habitat: Roadsides in forest, 750–2400 m. Voucher: SK 0015 (EA, HIB).

***Sida ternata* L.f.** – Life form: Herb. Habitat: Dry montane forest, 1350–3280 m. Voucher: Taylor 1586 (EA).

***Sparmannia ricinocarpa* (Eckl. & Zeyh.) Kuntze** – Life form: Shrub. Habitat: Upland grassland with moist forest edges, 1550–3380 m. Voucher: Blake 10858 (EA).

***Triumfetta brachyceras* K.Schum.** – Life form: Woody herb or shrub. Habitat: Moist forest clearings and margins, 1200–3000 m. Voucher: Musili et al. 187 (EA).

***Triumfetta longicornuta* Hutch. & M.B.Moss** – Life form: Shrub. Habitat: Glades in dry evergreen forest, 1350–2150 m. Voucher: SK 0125 (EA, HIB).

***Triumfetta pilosa* Roth** – Life form: Shrub. Habitat: Moist forest and swamp edges, 1200–2250 m. Voucher: Stroud 78 6732 (EA).

**F92. Meliaceae**

***Ekebergia capensis* Sparrm.** – Life form: Tree. Habitat: Montane forest and riparian forest, 600–2750 m. Voucher: Gardner 380 (EA).

***Lepidotrichilia volkensii* (Gürke) Leroy** – Life form: Tree. Habitat: Upland forest margins, 1550–2600 m. Voucher: Napper 1491 (EA).

***Trichilia dregeana* Sond.** – Life form: Tree. Habitat: Moist forest and riparian forest, 775–1800 m. Voucher: Gachathi 2/81 (EA).

***Turraea abyssinica* Hochst. ex A.Rich.** – Life form: Shrub or small tree. Habitat: Montane forest, 1820–2225 m. Voucher: Gardner 542 (EA).

**Turraea mombassana subsp. cuneata (Gürke) Styles & F.White** – Life form: Shrub or small tree. Habitat: Upland dry forest and bushlands, 1525–2225 m. Vouchers: Faden 6742, Hansen 760 (EA).

**F93. Melianthaceae**

***Bersama abyssinica* Fresen.** – Life form: Tree. Habitat: Upland moist forest, 1140–2550 m. Vouchers: Davidse 7058 (EA), SK 0013, SK 0139, SK 0233 (EA, HIB).

**F94. Menispermaceae**

***Cissampelos friesiorum* Diels** – Life form: Herbaceous climber. Habitat: Upland moist forest, 2000–2100 m. Voucher: Fries 1625 (EA).

**Stephania abyssinica var. abyssinica (Quart.-Dill & A.Rich.) Walp.** – Life form: Herbaceous climber. Habitat: Moist shaded sites in wooded grassland, 1450–3500 m. Vouchers: SAJIT 006465, SK 0035 (EA, HIB).

**Stephania abyssinica var. tomentella (Oliv.) Diels** – Life form: Herbaceous climber. Habitat: Moist shaded sites in wooded grassland, 1450–3500 m. Voucher: SK 0159 (EA, HIB).

**F95. Moraceae**

***Dorstenia afromontana* R.E.Fr.** – Life form: Herb. Habitat: Upland rainforest, 2000–2600 m. Voucher: Kirika et al. 76 (EA).

***Dorstenia hildebrandtii* Engl.** – Life form: Herb. Habitat: Epiphytic in moist forest and stream banks, 300–2170 m. Voucher: Napier 2182 (EA).

**Ficus cordata subsp. salicifolia (Vahl) C.C.Berg** – Life form: Tree. Habitat: Riparian forest and seasonal streams, 950–2400 m. Vouchers: Verdcourt 3548, Makin 26 (EA).

***Ficus sur* Forssk.** – Life form: Tree. Habitat: Riverine forest, 350–2500 m. Vouchers: SK 0228, SK 0229 (EA, HIB).

***Ficus thonningii* Blume** – Life form: Tree. Habitat: Moist forest, 350–2500 m. Voucher: Kamau 310 (EA).

**F96. Myricaceae**

**Morella salicifolia subsp. meyeri-johannis (Engl.) Verdc. & Polhill.** – Life form: Tree. Habitat: Upland grassland and moorland, 2700–3700 m. Vouchers: Beentje 3240, Thairu 16861 (EA).

**F97. Myrtaceae**

*Corymbia calophylla (R.Br. ex Lindl.) K.D.Hill & L.A.S.Johnson – Life form: Exotic tree. Habitat: Cultivated in roadsides and moist forest edges. Voucher: Muasya 2020 (EA).

*Corymbia gummifera (Gaertn.) K.D.Hill & L.A.S.Johnson – Life form: Exotic tree. Habitat: Cultivated in roadsides and moist forest edges. Voucher: Pudden 16 (EA).

*Eucalyptus crebra F.Muell. – Life form: Exotic tree. Habitat: Cultivated in roadsides and moist forest edges. Voucher: Greenway 8762 (EA).

*Eucalyptus globulus subsp. maidenii (F.Muell.) J.B.Kirkp. – Life form: Exotic tree. Habitat: Cultivated in roadsides and moist forest edges. Vouchers: Verdcourt 12800, Forest Dept 16083 (EA).

*Eucalyptus longifolia Link & Otto. – Life form: Exotic tree. Habitat: Cultivated in roadsides and moist forest edges. Voucher: Forest Dept 16098 (EA).

*Eucalyptus microcorys F.Muell. – Life form: Exotic tree. Habitat: Cultivated in roadsides and moist forest edges. Voucher: Forest Dept 16108 (EA).

*Eucalyptus muelleriana Howitt – Life form: Exotic tree. Habitat: Cultivated in roadsides and moist forest edges. Voucher: Pudden 26 (EA).

*Eucalyptus obliqua L’Hér. – Life form: Exotic tree. Habitat: Cultivated in roadsides and moist forest edges. Voucher: Pudden 054 (EA).

*Eucalyptus paniculata Sm. – Life form: Exotic tree. Habitat: Cultivated in roadsides and moist forest edges. Voucher: Stuart 2 (EA).

*Eucalyptus pellita F.Muell. – Life form: Exotic tree. Habitat: Cultivated in roadsides and moist forest edges. Voucher: Greenway 8754 (EA).

*Eucalyptus punctata A.Cunn. ex DC. – Life form: Exotic tree. Habitat: Cultivated in roadsides and moist forest edges. Voucher: Darling 31 (EA).

*Eucalyptus siderophloia Benth. – Life form: Exotic tree. Habitat: Cultivated in roadsides and moist forest edges. Voucher: Verdcourt 1963 (EA).

*Eucalyptus viminalis Labill. – Life form: Exotic tree. Habitat: Cultivated in roadsides and moist forest edges. Voucher: Forest Dept. 16137 (EA).

**Syzygium guineense subsp. afromontanum F.White** – Life form: Exotic tree. Habitat: Upland riverine and moist forest, 1500–2550 m. Vouchers: Battiscombe 555, Moore 762 (EA).

**F98. Ochnaceae**

***Ochna holstii* Engl.** – Life form: Tree. Habitat: Upland moist forest, 900–2350 m. Voucher: Polhill 165 (EA).

***Ochna insculpta* Sleumer** – Life form: Tree. Habitat: Evergreen forest, 1050–2450 m. Voucher: Agnew et al. 7939 (EA).

**F99. Olacaceae**

***Strombosia scheffleri* Engl.** – Life form: Tree. Habitat: Moist forest, 800–2500 m. Voucher: SK 0028 (EA, HIB).

**F100. Oleaceae**

***Chionanthus mildbraedii* (Gilg & G.Schellenb.) Stearn** – Life form: Tree. Habitat: Upland moist forest and riverine forest, 1200–2100 m. Voucher: SK 0238 (EA, HIB).

*Fraxinus pennsylvanica Marshall – Life form: Exotic tree. Habitat: Cultivated in roadsides. Voucher: SK 0127 (EA, HIB).

***Jasminum abyssinicum* Hochst. ex DC.** – Life form: Woody climber. Habitat: Forest undergrowth and forest margins, 690–3000 m. Voucher: McDonald 1336 (EA).

***Jasminum schimperi* Vatke** – Life form: Woody climber. Habitat: Rainforest and wooded grassland, 690–3000 m. Voucher: Miss Mainwaring 2404 (EA).

***Olea europaea* L.** – Life form: Tree. Habitat: Upland wet forest, 950–2400 m. Voucher: Napper 1697 (EA).

***Olea capensis* L.** – Life form: Tree. Habitat: Upland dry forest, 1150–2680 m. Voucher: SK 0105 (EA, HIB).

**F101. Onagraceae**

***Epilobium hirsutum* L.** – Life form: Herb. Habitat: Damp sites in upland grassland and moorland, 1190–2590 m. Voucher: SK 0237 (EA, HIB).

***Epilobium stereophyllum* Fresen.** – Life form: Herb. Habitat: Damp sites in upland grassland and moorland, 1750–3500 m. Voucher: SK 0116 (EA, HIB).

*Fuchsia arborescens Sims – Life form: Exotic shrub or tree. Habitat: Moist forest, 1220–2490 m. Voucher: SK 0130 (EA, HIB).

**Ludwigia adscendens subsp. diffusa (Forssk.) P.H.Raven** – Life form: Herb. Habitat: Swamps and freshwater pools, 600–1900 m. Voucher: SK 0161 (EA, HIB).

**F102. Orobanchaceae**

***Hedbergia abyssinica* (Benth.) Molau** – Life form: Herb. Habitat: Montane grassland and forest margins, 2000–3980 m. Voucher: Hedberg 1646 (EA).

***Orobanche minor* Sm.** – Life form: Herb. Habitat: Forest edges and disturbed grounds, 540–3000 m. Voucher: Townsend 2294 (EA).

***Orobanche ramosa* L.** – Life form: Herb. Habitat: Roadsides in upland grassland and woodland, 1735–2250 m. Vouchers: The Wallis 15436, Scaham 22 (EA).

**F103. Oxalidaceae**

***Oxalis corniculata* L.** – Life form: Herb. Habitat: Forest glades, 0–3600 m. Voucher: SAJIT 006481 (EA, HIB).

**F104. Papaveraceae**

***Corydalis cornuta* Royle** – Life form: Herb. Habitat: Montane forest up to the moorland, 2300–3300 m. Voucher: Napier 720 (EA).

***Corydalis mildbraedii* Fedde** – Life form: Herb. Habitat: Montane forest up to moorland, 2200–3600 m. Vouchers: SAJIT 006463, SK 0176 (EA, HIB).

***Fumaria abyssinica* Hammar** – Life form: Herb. Habitat: Montane forest up to moorland, 1300–3200 m. Voucher: Verdcourt 3207 (EA).

**F105. Passifloraceae**

**Adenia globosa subsp. pseudoglobosa (Verdc.) W.J. de Wilde** – Life form: Woody climber. Habitat: Deciduous and dry evergreen forest, 0–1850 m. Vouchers: Verdcourt 2677, 3547 (EA).

***Adenia gummifera* (Harv.) Harms** – Life form: Woody climber. Habitat: Dry or moist forest and bushland, 0–1850 m. Voucher: SK 0195 (EA, HIB).

*Passiflora edulis Sims – Life form: Exotic herbaceous climber. Habitat: Moist forest edges and bush thickets, 0–2500 m. Voucher: Greenway 10899 (EA).

*Passiflora mollissima L.H.Bailey – Life form: Exotic woody climber. Habitat: Moist forest edges, 1000–3000 m. Voucher: SK 0065 (EA, HIB).

***Passiflora subpeltata* (Kunth) L.H.Bailey** – Life form: Herbaceous climber. Habitat: Moist forest edges, 1500–2060 m. Voucher: SK 0003 (EA, HIB).

**F106. Penaeaceae**

***Olinia rochetiana* A.Juss.** – Life form: Tree. Habitat: Upland dry and moist evergreen forest, 1700–3100 m. Vouchers: Verdcourt 3283, Holyoak 712 (EA).

**F107. Phytolaccaceae**

***Phytolacca dodecandra* L’Hér.** – Life form: Shrub. Habitat: Riparian vegetation and bushland, 500–2400 m. Vouchers: SK 0032, SK 0236 (EA, HIB).

**F108. Piperaceae**

***Peperomia abyssinica* Miq.** – Life form: Herb. Habitat: Moist montane forest, 1600–2950 m. Voucher: SK 0142 (EA, HIB).

***Piper capense* L.f.** – Life form: Herb or subshrub. Habitat: Upland swampy forest edges and wet forest floors, 1200–2700 m. Voucher: SK 0134 (EA, HIB).

**F109. Pittosporaceae**

***Pittosporum viridiflorum* Sims** – Life form: Tree. Habitat: Upland moist forest, 900–2400 m. Voucher: Faden et al. 74/858 (EA).

**F110. Plantaginaceae**

***Callitriche oreophila* Schotsman** – Life form: Herb. Habitat: Aquatic in water pools and streams, 1150–3300 m. Voucher: Chandler 2322 (EA).

***Callitriche vulcanicola* Schotsman** – Life form: Herb. Habitat: Moist grounds in montane grassland, 3000–4050 m. Vouchers: Hedberg 1650, Gilbert and Thulin 1047 (EA).

***Plantago palmata* Hook.f.** – Life form: Herb. Habitat: Roadsides and clearings in upland forest, 1170–3300 m. Voucher: Bally 8519 (EA).

***Veronica abyssinica* Fresen.** – Life form: Herb. Habitat: Upland forest and bushy grassland, 1200–3900 m. Vouchers: SK 0050, SAJIT 006464 (EA, HIB).

***Veronica anagallis-aquatica* L.** – Life form: Herb. Habitat: Stream-sides, 480–2400 m. Voucher: Glover & Samuel 445 (EA).

***Veronica glandulosa* Hochst. ex Benth.** – Life form: Herb. Habitat: Upland forest margins, 2850–3980 m. Voucher: Mabberlay 350 (EA).

**F111. Polygalaceae**

***Polygala ohlendorfiana* Eckl. & Zeyh.** – Life form: Herb. Habitat: Upland grassland, 1800–3050 m. Voucher: Beentje 2658 (EA).

***Polygala sadebeckiana* Gürke** – Life form: Herb or subshrub. Habitat: Upland grassland, 10–2500 m. Voucher: SK 0257 (EA, HIB).

***Polygala sphenoptera* Fresen.** – Life form: Herb or subshrub. Habitat: Wooded grassland and bushland, 0–3300 m. Voucher: Kokwaro 2797 (EA).

***Polygala steudneri* Chodat** – Life form: Herb. Habitat: Upland grassland, 3000–4050 m. Voucher: Coe 775 (EA).

**F112. Polygonaceae**

***Harpagocarpus snowdenii* Hutch. & Dandy** – Life form: Herbaceous climber. Habitat: Upland moist forest, 1350–2650 m. Voucher: Gedye 6700 (EA).

***Oxygonum sinuatum* (Hochst. & Steud ex Meisn.) Dammer** – Life form: Herb. Habitat: Waste places in grassland, 0–2250 m. Voucher: Margareta 6 (EA).

***Oxygonum stuhlmannii* Dammer** – Life form: Herb. Habitat: Waste places in grassland, 10–2250 m. Voucher: Aement et al. 150 (EA).

***Persicaria decipiens* (R.Br.) K.L.Wilson** – Life form: Herb. Habitat: Wet places often in water pools, 1100–2291 m. Voucher: SK 0162 (EA, HIB).

***Persicaria nepalensis* (Meisn.) H.Gross** – Life form: Herb. Habitat: Upland grassland and forest edges, 1140–3500 m. Voucher: Kimani 9 (EA).

***Persicaria setosula* (A.Rich.) K.L.Wilson** – Life form: Herb. Habitat: Along streams in upland forest, 1050–2670 m. Voucher: Kerfoot 609 (EA).

***Persicaria strigosa* (R.Br.) Nakai** – Life form: Herb. Habitat: Upland moist forest and river banks, 1110–1920 m. Voucher: Faden 68/706 (EA).

***Polygonum afromontanum* Greenway** – Life form: Shrub. Habitat: Upland rainforest and moorland, 2100–3490 m. Voucher: Townsend 2316 (EA).

*Polygonum aviculare L. – Life form: Exotic herb. Habitat: Upland roadsides and disturbed areas, 2100–3490 m. Voucher: Albrechtsen 5238 (EA).

*Rumex acetosella L. – Life form: Exotic herb. Habitat: Montane grassland, 2400–3160 m. Voucher: Luke 15363 (EA).

***Rumex nepalensis* Spreng.** – Life form: Herb. Habitat: Upland grassland and bushland, 690–3700 m. Voucher: Blain 10915 (EA).

***Rumex ruwenzoriensis* Chiov.** – Life form: Herb. Habitat: Upland grassland and moorland, 1950–3700 m. Voucher: SK 0172 (EA, HIB).

**F113. Portulacaceae**

***Portulaca nitida* (Danin & H.G.Baker) Ricceri & Arrigoni** – Life form: Herb. Habitat: Roadsides and waste places in grassland, 0–2350 m. Voucher: Faden 74/835 (EA).

**F114. Primulaceae**

***Lysimachia arvensis* (L.) U.Manns & Anderb.** – Life form: Herb. Habitat: Dry evergreen bushland, 1350–2635 m. Voucher: Verdcourt 715 (EA).

***Lysimachia hexamera* (P.Taylor) U.Manns & Anderb.** – Life form: Herb. Habitat: Swampy sites in upland grassland, 2100–2600 m. Voucher: Hedberg 1085(EA).

***Lysimachia serpens* (Hochst. ex A.DC.) U.Manns & Anderb.** – Life form: Herb. Habitat: Damp sites in moorland, 2600–3960 m. Vouchers: Kuchar 9604 (EA), SK 0074 (EA, HIB).

***Ardisiandra sibthorpioides* Hook.f.** – Life form: Herb. Habitat: Moist evergreen forest, 900–2670 m. Voucher: Chandler 2227 (EA).

***Ardisiandra wettsteinii* J.Wagner** – Life form: Herb. Habitat: Upland moist forest and bamboo thickets, 1580–3600 m. Voucher: Albrechtsen 5946 (EA).

**^E^*Embelia keniensis* R.E.Fr.** – Life form: Tree. Habitat: Upland moist forest, 1500–2100 m. Voucher: Luke 447 (EA).

***Lysimachia ruhmeriana* Vatke** – Life form: Herb. Habitat: Wet montane forest, 2020–3500 m. Voucher: Kirika and York 1061 (EA).

***Maesa lanceolata* Forssk.** – Life form: Shrub or tree. Habitat: Riverine forest and moist forest margins, 360–2800 m. Voucher: Wimbush 1118 (EA).

***Myrsine africana* L.** – Life form: Shrub. Habitat: Upland wooded grassland, 1200–3600 m. Voucher: SK 0055 (EA, HIB).

***Rapanea melanophloeos* (L.) Mez** – Life form: Tree. Habitat: Moist forest, 900–3800 m. Voucher: SK 0101 (EA, HIB).

**F115. Proteaceae**

***Faurea arborea* Engl.** – Life form: Tree. Habitat: Upland dry forest, 1280–3100 m. Voucher: Gardner 7063 (EA).

***Faurea rochetiana* (A.Rich.) Chiov. ex Pic.Serm.** – Life form: Tree. Habitat: Wooded grassland, 900–2400 m. Voucher: Dyson 745 (EA).

***Faurea saligna* Harv.** – Life form: Tree. Habitat: Grassland with scattered trees, 700–1800 m. Voucher: Bono 10 (EA).

**Protea caffra subsp. kilimandscharica (Engl.) Chisumpa & Brummitt** – Life form: Shrub. Habitat: Montane grassland and forest edges, 2300–3700 m. Vouchers: Beentje 3241 (EA), SK 0110 (EA, HIB).

***Protea gaguedi* J.F.Gmel.** – Life form: Tree. Habitat: Woodland and scattered-tree grassland, 900–2100 m. Voucher: Holyoak 711 (EA).

**F116. Putranjivaceae**

**Drypetes gerrardii var. gerrardii Hutch.** – Life form: Tree. Habitat: Dry or moist evergreen forest, 1150–2300 m. Voucher: Fries 234 (EA).

**Drypetes gerrardii var. tomentosa Radcl.-Sm.** – Life form: Tree. Habitat: Upland evergreen forest, 1150–2000 m. Voucher: Kirika 496 (EA).

**F117. Ranunculaceae**

***Anemone thomsonii* Oliv.** – Life form: Herb. Habitat: Wet rocky sites in moorland, 2500–4000 m. Vouchers: SK 0182, SK 0054 (EA, HIB).

***Clematis simensis* Fresen.** – Life form: Woody climber. Habitat: Bushland and forest margins, 1000–3360 m. Vouchers: SK 0027, SK 0081 (EA, HIB).

***Delphinium macrocentrum* Oliv.** – Life form: Herb. Habitat: Upland grassland and bamboo thicket margins, 1650–3900 m. Voucher: SK 0107 (EA, HIB).

**^E^*Ranunculus aberdaricus* Ulbr.** – Life form: Herb. Habitat: Clearings in moist bamboo forest, 2550–3660 m. Voucher: Coe 789 (EA).

***Ranunculus multifidus* Forssk.** – Life form: Herb. Habitat: Stream banks and moist bushland, 1170–3450 m. Voucher: Mathenge 23 (EA).

***Ranunculus oreophytus* Delile** – Life form: Herb. Habitat: Wet and boggy places in moorland, 2240–4200 m. Voucher: Milne-Redhead et al. 1617 (EA).

***Ranunculus stagnalis* Hochst. ex A.Rich.** – Life form: Herb. Habitat: Bogs and water pools in moorland, 3000–4750 m. Voucher: Hedberg 994 (EA).

***Ranunculus volkensii* Engl.** – Life form: Herb. Habitat: Marshy sites in moorland, 2700–4050 m. Voucher: SK 0119 (EA, HIB).

***Thalictrum rhynchocarpum* Quart.-Dill. & A.Rich.** – Life form: Herb. Habitat: Undergrowth in upland forest, 1550–3275 m. Voucher: SK 0145 (EA, HIB).

**F118. Resedaceae**

***Caylusea abyssinica* (Fresen.) Fisch. & C.A.Mey.** – Life form: Herb. Habitat: Roadsides in upland grassland, 1100–3000 m. Voucher: SK 0124 (EA, HIB).

**F119. Rhamnaceae**

***Helinus mystacinus* (Aiton.) E.Mey. ex Steud.** – Life form: Woody climber. Habitat: Wooded grassland and forest margins, 100–2400 m. Voucher: SK 0019 (EA, HIB).

***Rhamnus prinoides* L’Hér.** – Life form: Tree. Habitat: Upland moist forests and bushland, 1500–3700 m. Vouchers: SK 0006, SK 0263 (EA, HIB).

***Rhamnus staddo* A.Rich.** – Life form: Tree. Habitat: Upland evergreen bushland and dry forest margins, 1000–3600 m. Voucher: SAJIT 006504 (EA, HIB).

***Scutia myrtina* (Burm.f.) Kurz** – Life form: Shrub. Habitat: Forest margins and bushland, 0–2750 m. Voucher: SK 0266 (EA, HIB).

**F120. Rhizophoraceae**

***Cassipourea celastroides* Alston** – Life form: Shrub or small tree. Habitat: Rocky hills in evergreen bushland, 250–1850 m. Voucher: Fries 2103 (EA).

***Cassipourea gummiflua* Tul.** – Life form: Tree. Habitat: Upland wet evergreen forest, 2000–2300 m. Vouchers: Luke et al. 7171, Medley 665 (EA).

***Cassipourea malosana* (Baker.) Alston** – Life form: Tree. Habitat: Moist or dry forest, 750–2600 m. Voucher: SK 0025 (EA, HIB).

**F121. Rosaceae**

***Alchemilla argyrophylla* Oliv.** – Life form: Shrub. Habitat: Damp sites in moorland, 2250–4650 m. Vouchers: Vorontsova 43 (EA), SK 0053 (EA, HIB).

***Alchemilla cryptantha* Steud. ex A.Rich.** – Life form: Herb. Habitat: Moist moorland and bamboo thickets, 1300–4050 m. Voucher: Townsend 2436 (EA).

**Alchemilla abyssinica subsp. cyclophylla (T.C.E.Fr.) Kalheber** – Life form: Herb. Habitat: Moist moorland and bamboo thickets, 2900–4300 m. Voucher: SK 0095 (EA, HIB).

***Alchemilla elgonensis* Mildbr.** – Life form: Shrub. Habitat: Moist moorland and bamboo thickets, 2700–4250 m. Voucher: Verdcourt 3777 (EA).

***Alchemilla ellenbeckii* Engl.** – Life form: Herb. Habitat: Moist moorland and bamboo thickets, 2100–3900 m. Voucher: Hedberg 1501 (EA).

***Alchemilla fischeri* Engl.** – Life form: Herb. Habitat: Moist montane forest and bamboo thickets, 2320–3440 m. Voucher: Muasya et al. 018(EA).

***Alchemilla pedata* Hochst. ex A.Rich.** – Life form: Herb. Habitat: Damp sites in upland grassland, 2120–3120 m. Voucher: Napier 683 (EA).

***Alchemilla hageniae* T.C.E.Fr.** – Life form: Herb. Habitat: Moist bamboo thickets and montane evergreen bushland, 3000–3490 m. Voucher: Muasya et al. 052 (EA).

***Alchemilla johnstonii* Oliv.** – Life form: Shrub. Habitat: Moist moorland and bamboo thickets, 2400–4260 m. Voucher: Mabberley 324 (EA).

***Alchemilla kiwuensis* Engl.** – Life form: Herb. Habitat: Moist upland forest and bamboo forest, 1250–3000 m. Voucher: Verdcourt 601 (EA).

***Alchemilla microbetula* T.C.E.Fr.** – Life form: Herb. Habitat: Moist moorland, 3350–4400 m. Voucher: Hedberg 4430 (EA).

***Alchemilla rothii* Oliv.** – Life form: Herb. Habitat: Moorland and upper edges of montane forest, 2700–4000 m. Voucher: Kuchar 12468(EA).

***Cliffortia nitidula* (Engl.) R.E.Fr. & T.C.E.Fr.** – Life form: Shrub. Habitat: Damp sites in moorland, 2040–3150 m. Voucher: Kuchar 10289 (EA).

***Fragaria vesca* L.** – Life form: Herb. Habitat: Upland grassland and forest edges, 2400–2850 m. Voucher: Mungai 50 (EA).

***Hagenia abyssinica* (Bruce ex Steud.) J.F.Gmel.** – Life form: Tree. Habitat: Upland moist forest, 2400–3600 m. Voucher: SK 0122 (EA, HIB).

***Prunus africana* (Hook.f.) Kalkman** – Life form: Tree. Habitat: Upland moist forest and riverine forest, 1350–2750 m. Voucher: Moon 765 (EA).

***Rosa rubiginosa* L.** – Life form: Herb. Habitat: Upland forest margins, 1600–3000 m. Voucher: SK 0264 (EA, HIB).

***Rubus apetalus* Poir.** – Life form: Shrub. Habitat: Upland moist forest, 1275–2700 m. Voucher: Logic/Bally 7958 (EA).

***Rubus friesiorum* Gust.** – Life form: Shrub. Habitat: Upland moist forest, 3050–3400 m. Vouchers: Kuchar 12476 (EA), SK 0062 (EA, HIB).

***Rubus keniensis* Standl.** – Life form: Shrub. Habitat: Upland moist forest, 1950–2800 m. Voucher: SAJIT 006472 (EA, HIB).

***Rubus pinnatus* Willd.** – Life form: Shrub. Habitat: Upland moist forest and bamboo thickets, 2400–3000 m. Voucher: Kuchar 7838 (EA).

***Rubus scheffleri* Engl.** – Life form: Shrub. Habitat: Upland moist forest margins and evergreen bushland, 1650–3150 m. Voucher: Grant 1217 (EA).

***Rubus steudneri* Schweinf.** – Life form: Shrub. Habitat: Upland moist forest margins and evergreen bushland, 1500–3480 m. Vouchers: Kuchar 8311, Kuchar and Msafiri 5206 (EA).

***Rubus volkensii* Engl.** – Life form: Shrub. Habitat: Upland moist forest margins and open sites, 2100–3450 m. Voucher: SAJIT 006480 (EA, HIB).

**F122. Rubiaceae**

***Anthospermum herbaceum* L.f.** – Life form: Herb. Habitat: Woodland and forest edges, 900–3240 m. Voucher: Kerfoot 477 (EA).

***Anthospermum usambarense* K.Schum.** – Life form: Shrub. Habitat: Moorland and upper edges of montane forest, 1300–4050 m. Voucher: Gardner 1113 (EA).

**Canthium oligocarpum subsp. friesiorum (Robyns) Bridson** – Life form: Shrub or tree. Habitat: Upland wet evergreen forest, 2000–2500 m. Vouchers: Battiscombe 1049, Venn Fey 11703 (EA).

***Chassalia kenyensis* Verdc.** – Life form: Shrub. Habitat: Moist evergreen forest, 1650–2300 m. Voucher: Polhill 360 (EA).

***Galiniera saxifraga* (Hochst.) Bridson** – Life form: Tree. Habitat: Upland moist forest and stream-sides, 1700–3000 m. Voucher: SK 0234 (EA, HIB).

***Galium acrophyum* Hochst. ex Chiov.** – Life form: Herb. Habitat: Open areas in bamboo thickets and montane forest, 2700–3200 m. Voucher: Kokwaro 1947 (EA).

***Galium aparinoides* Forssk.** – Life form: Herb. Habitat: Wet evergreen forest, 1680–3700 m. Voucher: Verdcourt, Cooley and Howard 3766G (EA).

**Galium glaciale var. satimmae Verdc.** – Life form: Herb. Habitat: Damp places in moorland, 3510–4350 m. Voucher: Coe & Kirika 284 (EA).

***Galium kenyanum* Verdc.** – Life form: Herb. Habitat: Upper margins of montane forest and moorland, 2880–3550 m. Voucher: Polhill 239 (EA).

***Galium ossirwaense* K.Krause** – Life form: Herb. Habitat: Upland moist forest edges, 2160–3750 m. Vouchers: Kuchar 12353, Napper 536 (EA).

***Galium ruwenzoriense* (Cortesi) Chiov.** – Life form: Herb. Habitat: Open upland forests or bushland, 2700–4000 m. Voucher: Hansen 870 (EA).

***Galium simense* Fresen.** – Life form: Herb. Habitat: Upland bushland and woodland, 1500–2700 m. Voucher: Taiti 2027 (EA).

**Galium spurium subsp. africanum Verdc.** – Life form: Herb. Habitat: Upland bushland and forest edges, 1250–2700 m. Vouchers: Knox 3213, Kanore Kibui 16 (EA).

***Galium thunbergianum* Eckl. & Zeyh.** – Life form: Herb. Habitat: Wet montane forest, 2000–3750 m. Voucher: Knox 3091 (EA).

***Galium scioanum* Chiov.** – Life form: Herb. Habitat: Upland swamps and riversides, 1800–2700 m. Voucher: SK 0093 (EA, HIB).

***Lasianthus kilimandscharicus* K.Schum.** – Life form: Shrub or small tree. Habitat: Undergrowth in moist forest, 1500–2500 m. Voucher: Hanse 834 (EA).

**Mussaenda microdonta subsp. odorata (Hutch.) Bridson** – Life form: Shrub or tree. Habitat: Upland moist evergreen forest and riparian forest, 1830–2100 m. Vouchers: Greenway 9680, Kirika et al. 6 (EA).

***Oldenlandia friesiorum* Bremek** – Life form: Herb. Habitat: Upland evergreen forest and forest edges, 1800–2550 m. Voucher: Kuchar 8348 (EA).

***Oldenlandia monanthos* (Hochst. ex A.Rich.) Hiern** – Life form: Herb. Habitat: Upland evergreen forest and montane grassland, 1350–3500 m. Voucher: Lind 2883 (EA).

***Pauridiantha paucinervis* (Hiern) Bremek.** – Life form: Shrub or small tree. Habitat: Upland moist evergreen forest, 500–2400 m. Voucher: SK 0244 (EA, HIB).

**Pavetta abyssinica var. lamurensis Verdc.** – Life form: Tree. Habitat: Upland forest and bushland, 1500–2550 m. Vouchers: Luke 705, Gardner 2837 (EA).

***Pentanisia foetida* Verdc.** – Life form: Herb. Habitat: Upland grassland and forest margins, 1830–2300 m. Voucher: De Block & Stieperaere 503 (EA).

***Pentas lanceolata* (Forssk.) Deflers** – Life form: Herb or subshrub. Habitat: Roadsides in upland grassland, 1200–2830 m. Voucher: Young 1004 (EA).

***Psychotria fractinervata* E.M.A.Petit** – Life form: Tree. Habitat: Upland wet forest, 1800–2600 m. Vouchers: SK 0128, SK 0135, SK 0241 (EA, HIB).

***Psychotria kirkii* Hiern** – Life form: Shrub. Habitat: Moist forests and open woodland, 250–2250 m. Voucher: SK 0152 (EA, HIB).

***Psychotria mahonii* C.H.Wright** – Life form: Tree. Habitat: Upland wet evergreen forest and swampy forest, 1230–2700 m. Voucher: SK 0243 (EA, HIB).

**Psydrax parviflora subsp. rubrocostata (Robyns) Bridson** – Life form: Tree. Habitat: Upland moist forest, 1375–2750 m. Vouchers: Someren 3534, Muhia 123 (EA).

***Psydrax schimperiana* (A.Rich.) Bridson** – Life form: Tree. Habitat: Moist forest and bushland, 15–2500 m. Voucher: SK 0005 (EA, HIB).

***Rothmannia manganjae* (Hiern) Keay** – Life form: Tree. Habitat: Moist forest, 230–1800 m. Voucher: Gardner 2477 (EA).

**Rubia cordifolia subsp. conotricha (Gand.) Verdc.** – Life form: Herbaceous climber. Habitat: Upland forest edges and bushland, 1140–3120 m. Vouchers: Verdcourt 620 (EA), SAJIT 006503 (EA, HIB)

***Rytigynia bugoyensis* (K.Krause) Verdc.** – Life form: Shrub. Habitat: Montane evergreen forest, 1230–2400 m. Voucher: Faden 67/142 (EA).

***Rytigynia uhligii* (K.Schum. & K.Krause) Verdc.** – Life form: Tree. Habitat: Montane dry or moist evergreen forest, 1000–2460 m. Voucher: SK 0200 (EA, HIB).

***Spermacoce princeae* (K.Schum.) Verdc.** – Life form: Herb. Habitat: Roadsides in bamboo thickets, 960–2650 m. Voucher: SK 0049 (EA, HIB).

***Vangueria apiculata* K.Schum.** – Life form: Tree. Habitat: Evergreen forest, bushland and riverine forest, 900–2330 m. Voucher: SK 0138 (EA, HIB).

***Vangueria infausta* Burch.** – Life form: Tree. Habitat: Evergreen forest margins and woodland, 30–2100 m. Voucher: Faden 6774 (EA).

***Vangueria madagascariensis* J.F.Gmel.** – Life form: Tree. Habitat: Moist forest and riparian forest, 0–2130 m. Voucher: SK 0037 (EA, HIB).

***Vangueria volkensii* K.Schum.** – Life form: Tree. Habitat: Dry forest margin and riverine forest, 900–2300 m. Voucher: SK 0235 (EA, HIB).

**F123. Rutaceae**

***Calodendrum capense* (L.f.) Thunb.** – Life form: Tree. Habitat: Upland evergreen and riverine forest, 1200–2200 m. Voucher: Kamau 326 (EA).

***Clausena anisata* (Willd.) Hook.f. ex Benth.** – Life form: Tree. Habitat: Upland moist forest, 0–2700 m. Voucher: SK 0129 (EA, HIB).

***Fagaropsis angolensis* (Engl.) Dale** – Life form: Tree. Habitat: Upland moist forest, 1000–2250 m. Voucher: Faden 74/895E (EA).

***Toddalia asiatica* (L.) Lam.** – Life form: Shrub. Habitat: Moist forest edges and wet bushland, 0–3000 m. Voucher: SK 0002 (EA, HIB).

***Vepris glandulosa* (Hoyle & Leakey) Kokwaro** – Life form: Tree. Habitat: Upland dry evergreen forest, 1700–2020 m. Voucher: Kirika 491 (EA).

**Vepris hanangensis var. unifoliata Kokwaro** – Life form: Tree. Habitat: Upland evergreen forest and bushland, 1700–2020 m. Vouchers: Kokwaro 4038 & 4039, Greenway 7595 (EA).

***Vepris nobilis* (Delile) Mziray** – Life form: Tree. Habitat: Dry evergreen forest and bushland, 900–2750 m. Voucher: SK 0169 (EA, HIB).

***Vepris simplicifolia* (Engl.) Mziray** – Life form: Tree. Habitat: Dry forest and evergreen forest and bushland, 300–2420 m. Voucher: Battiscombe 867 (EA).

***Vepris trichocarpa* (Engl.) Mziray** – Life form: Shrub. Habitat: Wooded grassland and riparian forest, 0–2660 m. Voucher: Stuhlmann 937 (EA).

***Zanthoxylum usambarense* (Engl.) Kokwaro** – Life form: Tree. Habitat: Upland dry forest, 1200–2600 m. Voucher: Greenway 12624 (EA).

**F124. Salicaceae**

***Casearia battiscombei* R.E.Fr.** – Life form: Tree. Habitat: Upland moist forest, 1000–2440 m. Voucher: Gachathi 1/81 (EA).

***Dovyalis abyssinica* (A.Rich.) Warb.** – Life form: Shrub or tree. Habitat: Upland moist forest, 1450–3000 m. Voucher: SK 0021 (EA, HIB).

***Oncoba routledgei* Sprague** – Life form: Shrub or small tree. Habitat: Upland moist forest and riparian vegetation, 900–2440 m. Voucher: Hargen 1186 (EA).

***Oncoba spinosa* Forssk.** – Life form: Shrub. Habitat: Moist forest edges and bushland, 0–1800 m. Voucher: Kirika et al. 168 (EA).

***Scolopia zeyheri* (Nees) Szyszył** – Life form: Tree. Habitat: Dry evergreen forest and bushland, 0–2400 m. Voucher: Verdcourt 3132 (EA).

***Trimeria grandifolia* (Hochst.) Warb.** – Life form: Tree. Habitat: Dry evergreen forest and bushland, 150–2500 m. Voucher: Napier 1933 (EA).

**F125. Santalaceae**

***Osyris lanceolata* Hochst. & Steud.** – Life form: Shrub or small tree. Habitat: Upland forest and bushland, 900–2700 m. Voucher: Robertson 7675 (EA).

***Thesium kilimandscharicum* Engl.** – Life form: Herb. Habitat: Montane grassland and moorland, 2200–4200 m. Voucher: Polhill 231 (EA).

***Thesium mukense* A.W.Hill** – Life form: Herb. Habitat: Upland grassland and woodland, 1100–2500 m. Voucher: Faden & Evans 74/622 (EA).

***Thesium radicans* Hochst. ex A.Rich.** – Life form: Herb. Habitat: Upland open grassland, 1800–3000 m. Voucher: Harwey Albuchtsen 186 (EA).

***Thesium schweinfurthii* Engl.** – Life form: Herb. Habitat: Upland grassland and woodland, 1050–2300 m. Voucher: Harwey Albuchtsen 187 (EA).

***Viscum tuberculatum* A.Rich.** – Life form: Shrub. Habitat: Upland dry evergreen forest and bushland, 1200–2400 m. Voucher: SK 0199 (EA, HIB).

**F126. Sapindaceae**

***Allophylus abyssinicus* (Hochst.) Radk.** – Life form: Tree. Habitat: Upland moist forest, 1050–2550 m. Voucher: Lind et al. 5029 (EA).

***Allophylus africanus* P.Beauv.** – Life form: Tree. Habitat: Wooded grassland and forest margins, 650–2400 m. Voucher: SK 0194 (EA, HIB).

***Blighia unijugata* Baker** – Life form: Tree. Habitat: Moist or dry evergreen forest and bushland, 0–1900 m. Voucher: Battiscombe 6 (EA).

***Deinbollia kilimandscharica* Taub.** – Life form: Tree. Habitat: Moist or dry evergreen forest and bushland, 1100–2400 m. Voucher: E.A.A. F.RO Staff 53 (EA).

**Dodonaea viscosa subsp. angustifolia (L.f.) J.G.West.** – Life form: Shrub. Habitat: Grassland and bushland, 0–2700 m. Voucher: SK 0098 (EA, HIB).

**F127. Sapotaceae**

***Chrysophyllum gorungosanum* Engl.** – Life form: Tree. Habitat: Upland moist forest, 1300–2250 m. Voucher: Hockliffe 1370 (EA).

**Pouteria adolfi-friedericii subsp. keniensis (R.E.Fr.) L.Gaut.** – Life form: Tree. Habitat: Upland moist forest, 1430–2500 m. Vouchers: Leaky 1224, Luke 408 (EA).

**F128. Scrophulariaceae**

***Hedbergia decurva* (Hochst. ex Benth.) A.Fleischm. & Heubl** – Life form: Herb. Habitat: Moorland and upper edges of montane forest, 2950–3840 m. Voucher: SK 0078 (EA, HIB).

***Hedbergia longiflora* (Hochst. ex Benth.) A.Fleischm. & Heubl** – Life form: Herb. Habitat: Moist montane forest and grassland, 2450–3500 m. Voucher: Kuchar 12763 (EA).

***Bartsia trixago* L.** – Life form: Herb. Habitat: Rocky sites in moorland, 930–3700 m. Voucher: Dale 2683 (EA).

***Craterostigma pumilum* Hochst.** – Life form: Herb. Habitat: Montane grassland, 2000–2600 m. Voucher: Verdcourt 1042 (EA).

***Cycnium tenuisectum* (Standl.) O.J.Hansen** – Life form: Herb. Habitat: Open places in montane forest, 1800–3500 m. Voucher: Nattrass 1459 (EA).

**Cycnium tubulosum subsp. montanum (N.E.Br.) O.J.Hansen.** – Life form: Herb. Habitat: Grassland, 200–2600 m. Vouchers: Harvey 178, Tallantire 675 (EA).

***Diclis bambuseti* R.E.Fr.** – Life form: Herb. Habitat: Moist montane forest, 2000–3720 m. Voucher: Kuchar 12729 (EA).

***Diclis ovata* Benth.** – Life form: Herb. Habitat: Upland moist grassland and montane forest, 1300–3440 m. Voucher: John Terry 178 (EA).

***Hebenstretia angolensis* Rolfe** – Life form: Herb. Habitat: Dry montane grassland and rocky heathland, 1500–4000 m. Voucher: SAJIT 006476, SK 0091 (EA, HIB).

***Limosella africana* Glück** – Life form: Herb. Habitat: Aquatic in upland waterfalls edges and stream banks, 1600–4200 m. Voucher: Gilbert et al. 1045 (EA).

***Limosella capensis* Thunb.** – Life form: Herb. Habitat: Aquatic in muddy water pools and slow flowing streams, 1800–2800 m. Voucher: Verdcourt 641 (EA).

***Limosella macrantha* R.E.Fr.** – Life form: Herb. Habitat: Moist depressions in montane forest and moorland, 2500–4500 m. Voucher: Napper 1232 (EA).

***Limosella maior* Diels** – Life form: Herb. Habitat: Aquatic in upland muddy water pools, 1800–2700 m. Voucher: Greenway and Hemming 8768 (EA).

***Lindernia rotundifolia* (L.) Alston** – Life form: Herb. Habitat: Roadsides in upland moist grassland, 1600–2000 m. Voucher: Agnew 7081 (EA).

***Selago thomsonii* Rolfe** – Life form: Herb. Habitat: Dry montane grassland, 1860–3380 m. Voucher: Greenway and Kanuri 15045 (EA).

***Sibthorpia europaea* L.** – Life form: Herb. Habitat: Moist montane forest, 2000–3750 m. Voucher: SAJIT 006469 (EA, HIB).

***Verbascum brevipedicellatum* (Engl.) Hub.-Mor.** – Life form: Herb. Habitat: Upland rocky grassland, 1550–4100 m. Voucher: Mbale 860 (EA).

***Verbascum scrophulariifolium* (Hochstetter) D.Hartl.** – Life form: Herb. Habitat: Upland grassland, 1800–3600 m. Voucher: Piers 10163 (EA).

**F129. Simaroubaceae**

***Brucea antidysenterica* J.F.Mill.** – Life form: Tree. Habitat: Upland dry or moist evergreen forest, 1400–2800 m. Voucher: Davidse 7054 (EA).

**F130. Solanaceae**

*Brugmansia suaveolens (Humb. & Bonpl. ex Willd.) Sweet – Life form: Exotic shrub or small tree. Habitat: Forest margins and roadsides, 500–1800 m. Voucher: SK 0226 (EA, HIB).

*Cestrum aurantiacum Lindl. – Life form: Exotic shrub. Habitat: Roadsides and disturbed areas, 850–2600 m. Voucher: SK 0158 (EA, HIB).

*Cestrum elegans (Brongn.) Schltdl. – Life form: Exotic shrub or small tree. Habitat: Disturbed sites in upland moist forest, 1350–2600 m. Voucher: SK 0262 (EA, HIB).

***Discopodium penninervium* Hochst.** – Life form: Shrub. Habitat: Moist upper parts of montane forest and moorland, 1400–3000 m. Voucher: SK 0082 (EA, HIB).

*Physalis philadelphica Lam. – Life form: Exotic herb. Habitat: Forest margins and bushland, 990–2250 m. Voucher: SK 0031 (EA, HIB).

*Physalis peruviana L. – Life form: Exotic herb. Habitat: Upland forest margins and bushland, 1700–2500 m. Voucher: Kamau 333 (EA).

***Solanum aculeastrum* Dunal** – Life form: Shrub or tree. Habitat: Upland forest margins and grassland, 1200–2500 m. Voucher: SK 0043 (EA, HIB).

***Solanum agnewiorum* Voronts.** – Life form: Shrub. Habitat: Moist montane forest, 1800–2500 m. Voucher: Hepper and Field 4923 (EA).

*Solanum americanum Mill. – Life form: Exotic herb. Habitat: Disturbed places in grassland and bushland, 0–3200 m. Voucher: Kerfoot 638 (EA).

***Solanum giganteum* Jacq.** – Life form: Shrub. Habitat: Upland grassland and bushland, 1500–2450 m. Voucher: Polhill & Verdcourt 267 (EA).

***Solanum incanum* L.** – Life form: Shrub. Habitat: Bushland and wooded grassland, 15–2200 m. Voucher: Bally and Smith 14628A (EA).

*Solanum laxum Spreng. – Life form: Exotic shrub. Habitat: Upland rainforest, 1600–2650 m. Voucher: Verdcourt 3026 (EA).

***Solanum mauense* Bitter** – Life form: Shrub. Habitat: Upland forest edges and bushland, 1800–3000 m. Voucher: Simpson 13390 (EA).

*Solanum mauritianum Scop. – Life form: Exotic shrub or small tree. Habitat: Upland forest margins and roadsides, 1150–2800 m. Voucher: SK 0151 (EA, HIB).

***Solanum phoxocarpum* Voronts.** – Life form: Shrub or tree. Habitat: Upland moist forest and woodland, 2100–3000 m. Voucher: Mbale et al. 844 (EA).

*Solanum pseudocapsicum L. – Life form: Exotic shrub. Habitat: Disturbed sites in upland forest, 1600–2150 m. Voucher: SK 0153 (EA, HIB).

***Solanum pseudospinosum* C.H.Wright** – Life form: Shrub. Habitat: Upland grassland and bushland, 2100–3600 m. Voucher: Snowden 564 (EA).

***Solanum runsoriense* C.H.Wright** – Life form: Shrub. Habitat: Upper parts of montane forest, 2400–3200 m. Voucher: Polhill 250 (EA).

***Solanum schumannianum* Dammer** – Life form: Shrub. Habitat: Upland forest clearings, roadsides and bushland, 1300–3000 m. Voucher: SK 0029 (EA, HIB).

***Solanum tarderemotum* Bitter** – Life form: Herb. Habitat: Moist forest margins and along streams, 550–2950 m. Voucher: Kerfoot 637 (EA).

***Solanum terminale* Forsk.** – Life form: Shrub. Habitat: Riverine forest and bushland, 400–3220 m. Voucher: SK 0040 (EA, HIB).

***Withania somnifera* (L.) Dunal** – Life form: Herb or subshrub. Habitat: Grassland and bushland, 0–2800 m. Voucher: SK 0024 (EA, HIB).

**F131. Stilbaceae**

***Halleria lucida* L.** – Life form: Shrub. Habitat: Dry forest, 900–2700 m. Voucher: SK 0104 (EA, HIB).

**F132. Thymelaeaceae**

***Gnidia glauca* (Fresen.) Gilg** – Life form: Tree. Habitat: Upland forest margins to bamboo zone, 2250–3300 m. Voucher: Kerfoot 1402 (EA).

***Peddiea fischeri* Engl.** – Life form: Tree. Habitat: Upland forest understorey and bushland, 950–2400 m. Voucher: Battiscombe 391 (EA).

**F133. Urticaceae**

***Didymodoxa caffra* (Thunb.) Friis & Wilmot-Dear** – Life form: Herb. Habitat: Dry upland forests, 1750–2840 m. Voucher: Faden & Evans 74/682 (EA).

***Droguetia debilis* Rendle** – Life form: Herb. Habitat: Undergrowth in upland moist forest, 1550–3090 m. Voucher: Agnew et al. 8181 (EA).

***Droguetia iners* (Forssk.) Schweinf.** – Life form: Herb. Habitat: Upland moist evergreen forest and edges of bamboo thicket, 1600–3250 m. Voucher: Agnew et al. 7118 (EA).

***Elatostema monticola* Hook.f.** – Life form: Herb. Habitat: Upland moist evergreen forest, 1600–2800 m. Voucher: Kerfoot 601 (EA).

***Girardinia bullosa* (Hochst. ex Steud.) Wedd.** – Life form: Herb. Habitat: Upland moist forest and forest margins, 1800–2600 m. Voucher: Mathenge 212 (EA).

***Girardinia diversifolia* (Link) Friis** – Life form: Herb. Habitat: Open sites in upland moist forest and margins, 1100–2500 m. Voucher: Agnew 7692 (EA).

***Laportea alatipes* Hook.f.** – Life form: Herb. Habitat: Undergrowth in upland moist forest, 1400–3500 m. Voucher: Verdcourt 2307 (EA).

***Myrianthus holstii* Engl.** – Life form: Tree. Habitat: Upland moist forest, 900–2100 m. Voucher: Hutchins (EA).

***Obetia radula* (Baker) Baker ex B.D.Jacks.** – Life form: Tree. Habitat: Dry forest margins, 700–2000 m. Voucher: Hansen 745 (EA).

***Parietaria debilis* G.Forst.** – Life form: Herb. Habitat: Upland moist forest and bamboo thickets, 1700–4200 m. Voucher: Kerfoot 655 (EA).

***Pilea rivularis* Wedd.** – Life form: Herb. Habitat: Upland moist forest, 1250–3100 m. Voucher: Kerfoot 468 (EA).

**Pilea usambarensis var. veronicifolia (Engl.) Friis** – Life form: Herb. Habitat: Upland moist forest, 2030–3160 m. Voucher: Agnew 7164 (EA).

**Pilea usambarensis var. engleri (Rendle) Friis** – Life form: Herb. Habitat: Upland rainforest, 1500–3160 m. Voucher: Fries and Fries 2785 (EA).

***Pilea johnstonii* Oliv.** – Life form: Herb. Habitat: Moist montane forest, 1450–2910 m. Voucher: SAJIT 006462 (EA).

***Urtica massaica* Mildbr.** – Life form: Herb. Habitat: Moist montane forest, 400–3320 m. Voucher: SK 0038 (EA, HIB).

***Urtica urens* L.** – Life form: Herb. Habitat: Disturbed sites in upland moist forest, 1800–2500 m. Voucher: Gillet 16765 (EA).

**F134. Verbenaceae**

*Glandularia aristigera (S.Moore) Tronc. – Life form: Exotic herb. Habitat: Roadsides in upland forest, 1650–2060 m. Voucher: Mungai 106 (EA).

*Lantana camara L. – Life form: Exotic shrub. Habitat: Open areas or clearings in forest and roadsides, 0–2040 m. Voucher: SK 0201 (EA, HIB).

*Lantana trifolia L. – Life form: Exotic shrub. Habitat: Grassland and bushland, 0–2400 m. Voucher: SK 0012 (EA, HIB).

**Lantana viburnoides subsp. viburnoides (Forssk.) Vahl** – Life form: Woody herb or shrub. Habitat: Bushland, 0–1950 m. Vouchers: Kamau 76 (EA), SK 0011 (EA, HIB).

***Lippia kituiensis* Vatke** – Life form: Woody herb or shrub. Habitat: Woodland and bushland, 405–2550 m. Voucher: Trapnell 2134 (EA).

***Verbena brasiliensis* Vell.** – Life form: Herb. Habitat: Woodland and bushland, 1050–2220 m. Voucher: Gillet 16285A (EA).

**F135. Violaceae**

***Viola abyssinica* Steud. ex Oliv.** – Life form: Herb. Habitat: Upland evergreen forest and bamboo thickets, 1200–3740 m. Voucher: Napier 723 (EA).

***Viola eminii* (Engl.) R.E.Fr.** – Life form: Herb. Habitat: Montane forest and moorlands, 2150–4050 m. Voucher: Napier 676 (EA).

***Viola nannae* R.E.Fr.** – Life form: Herb. Habitat: Montane grassland and heathland, 2550–3620 m. Voucher: SAJIT 006482 (EA, HIB).

**F136. Vitaceae**

***Cyphostemma kilimandscharicum* (Gilg) Desc. ex Wild & R.B.Drumm.** – Life form: Herbaceous climber. Habitat: Moist montane forest and bamboo thickets, 1590–3040 m. Vouchers: SK 0045, SK 0076 (EA, HIB).

***Cyphostemma maranguense* (Gilg) Desc.** – Life form: Herbaceous climber. Habitat: Dry upland forest and scattered-tree grasslands, 1500–2300 m. Voucher: Kirrika 498 (EA).

## Appendix I

**Table:** List of naturalised and exotic plants taxa in Aberdare Ranges Forest.

Family	Taxa	Native country	Life form
Apiaceae	*Torilis africana* Spreng.	Mediterranean region	Herb
Apocynaceae	*Asclepias physocarpa* (E. Mey.) Schltr.	Australia	Herb or shrublet
Asteraceae	*Acanthospermum glabratum* (DC.) Wild	South America	Herb
Asteraceae	*Erigeron bonariensis* L.	Central & South America	Herb
Asteraceae	*Schkuhria pinnata* (Lam.) Kuntze ex Thell.	Central & South America	Herb
Asteraceae	*Tagetes minuta* L.	South America	Herb
Asteraceae	*Tithonia diversifolia* A.Gray	Central America & South America	Woody herb
Bignoniaceae	*Jacaranda mimosifolia* D.Don	Central America & South America	Tree
Brassicaceae	*Brassica napus* L.	Europe	Herb
Brassicaceae	*Brassica rapa* L.	North Africa, Mediterranean, Middle East	Herb
Brassicaceae	*Lepidium bonariense* L	South America	Herb
Brassicaceae	*Raphanus raphanistrum* L.	Europe	Herb
Brassicaceae	*Raphanus sativus* L.	Europe	Herb
Cupressaceae	*Cupressus lusitanica* Mill.	North America	Tree
Dryopteridaceae	*Elaphoglossum piloselloides* (C.Presl) T.Moore	Central & South America	Herb
Euphorbiaceae	*Vernicia fordii* Airy Shaw	Southeast Asia	Tree
Fabaceae	*Caesalpinia decapetala* (Roth) Alston	India & Southeast Asia	Woody climber
Fabaceae	*Senna septemtrionalis* (Viv.) H.S.Irwin & Barneby	Mexico & Central America	Shrub
Fabaceae	*Vicia benghalensis* L.	South Europe & North Africa	Herb
Fabaceae	Vicia villosa subsp. varia (Host) Corb.	South Europe, North Africa, Middle East	Herb
Lythraceae	*Parsonsia micropetala* (Kunth) Standl.	Mexico	Shrub
Myrtaceae	*Corymbia calophylla* (Lindl.) K.D.Hill & L.A.S.Johnson	Australia	Tree
Myrtaceae	*Corymbia gummifera* (Gaertn.) K.D.Hill & L.A.S.Johnson	Australia	Tree
Myrtaceae	*Eucalyptus crebra* F.Muell.	Australia	Tree
Myrtaceae	Eucalyptus globulus subsp. maidenii (F.Muell.) J.B Kirkp.	Australia	Tree
Myrtaceae	*Eucalyptus longifolia* Link & Otto.	Australia	Tree
Myrtaceae	*Eucalyptus microcorys* F.Muell.	Australia	Tree
Myrtaceae	*Eucalyptus muelleriana* Howitt	Australia	Tree
Myrtaceae	*Eucalyptus obliqua* L’Hér.	Australia	Tree
Myrtaceae	*Eucalyptus paniculata* Sm.	Australia	Tree
Myrtaceae	*Eucalyptus pellita* F.Muell.	Australia	Tree
Myrtaceae	*Eucalyptus punctata* A. Cunn. ex DC.	Australia	Tree
Myrtaceae	*Eucalyptus siderophloia* Benth.	Australia	Tree
Myrtaceae	*Eucalyptus viminalis* Labill.	Australia	Tree
Oleaceae	*Fraxinus pennsylvanica* Marshall	North America	Tree
Onagraceae	*Fuchsia arborescens* Sims	South America	Shrub or tree
Passifloraceae	*Passiflora edulis* Sims	South America	Climbing herb
Passifloraceae	*Passiflora subpeltata* (Kunth) L.H.Bailey	Mexico & Central America	Climbing herb
Pinaceae	*Pinus patula* Schiede ex Schltdl & Cham.	Mexico	Tree
Poaceae	*Paspalum notatum* Flüggé	Mexico & South America	Herb
Polygonaceae	*Polygonum aviculare* L.	North Africa, Europe, Asia	Herb
Polygonaceae	*Rumex acetosella* L.	Europe & Asia	Herb
Solanaceae	*Brugmansia suaveolens* (Humb. & Bonpl. ex Willd.) Sweet	South America	Shrub
Solanaceae	*Cestrum aurantiacum* Lindl.	Mexico	Shrub
Solanaceae	*Cestrum elegans* (Brongn.) Schltdl.	North & South America	Shrub or tree
Solanaceae	*Physalis peruviana* L.	South America	Herb
Solanaceae	*Physalis philadelphica* Lam.	Mexico	Herb
Solanaceae	*Solanum americanum* Mill.	America	Herb
Solanaceae	*Solanum laxum* Spreng.	South America	Shrub
Solanaceae	*Solanum mauritianum* Scop.	South America	Shrub or tree
Solanaceae	*Solanum pseudocapsicum* L.	South America	Shrub
Verbenaceae	*Glandularia aristigera* (S.Moore) Tronc.	South America	Herb
Verbenaceae	*Lantana camara* L.	Central & South America	Shrub
Verbenaceae	*Lantana trifolia* L.	Central & South America	Shrub

## Appendix II

**Table:** List of endemic taxa in Aberdare Ranges Forest.

Family	Taxa	Life form	Other region of occurrence
Apiaceae	*Afrosciadium englerianum* (H.Wolff) P.J.D.Winter	Herb	Mt. Kenya
Apiaceae	Afrosciadium friesiorum (H.Wolff) P.J.D.Winter var. friesiorum	Herb	Mt. Kenya
Apiaceae	Afrosciadium friesiorum var. bipinnatum (C.C.Towns.) P.J.D.Winter	Herb	Mt. Kenya
Apocynaceae	*Brachystelma keniense* Schweinf.	Herb	Mt. Kenya
Asteraceae	*Carduus millefolius* R.E.Fr.	Herb	Mt. Kenya
Asteraceae	*Dendrosenecio brassiciformis* (R.E.Fr. & T.C.E.Fr.) Mabb.	Shrub	
Asteraceae	*Dendrosenecio keniensis* (Baker f.) Mabb.	Shrub	Mt. Kenya
Asteraceae	*Dendrosenecio keniodendron* (R.E.Fr. & T.C.E.Fr.) B.Nord.	Shrub	Mt. Kenya
Asteraceae	*Helichrysum brownei* S.Moore	Woody herb	Mt. Kenya
Asteraceae	*Helichrysum gloria-dei* Chiov.	Shrub	Mt. Kenya
Asteraceae	*Senecio amplificatus* C.Jeffrey	Herb	
Asteraceae	*Senecio margaritae* C.Jeffrey	Shrub	
Asteraceae	*Senecio roseiflorus* R.E.Fr.	Woody herb	Mt. Kenya
Cyperaceae	Carex runssoroensis var. aberdarensis Kük.	Herb	Mt. Kenya
Campanulaceae	Lobela gregoriana subsp. sattimae (R.E.Fr. & T.C.E.Fr.) E. B. Knox	Shrub	
Primulaceae	*Embelia keniensis* R.E.Fr.	Tree	Mt. Kenya
Poaceae	*Eragrostis amanda* Clayton	Herb	
Ranunculaceae	*Ranunculus aberdaricus* Ulbr.	Herb	

## Appendix III

**Table:** List of threatened taxa in Aberdare Ranges Forest according to the IUCN Red List.

Family	Taxa	Life form	IUCN
Acanthaceae	*Asystasia lorata* Ensermu Kelbessa	Herb	EN
Araliaceae	*Polyscias kikuyuensis* Summerh.	Tree	NT
Asteraceae	*Ethulia scheffleri* S.Moore	Herb	EN
Asteraceae	*Helichrysum ellipticifolium* Moeser	Herb	VU
Asteraceae	*Senecio amplificatus* C.Jeffrey	Herb	VU
Cyperaceae	*Carex monostachya* A.Rich.	Herb	VU
Cyperaceae	*Carex phragmitoides* Kük.	Herb	VU
Cyperaceae	*Cyperus afroalpinus* Lye	Herb	NT
Dryopteridaceae	Arachniodes webbiana var. foliosa (A.Braun) Schelpe	Herb	VU
Euphorbiaceae	*Croton alienus* Pax	Shrub	EN
Fabaceae	*Crotalaria jacksonii* Baker f.	Shrub	VU
Pinaceae	*Pinus radiata* D.Don	Tree	EN
Rosaceae	*Prunus africana* (Hook.f.) Kalkm.	Tree	VU
